# Masting by beech trees predicts the risk of Lyme disease

**DOI:** 10.1186/s13071-021-04646-0

**Published:** 2021-03-20

**Authors:** Cindy Bregnard, Olivier Rais, Maarten Jeroen Voordouw

**Affiliations:** 1 Laboratory of Ecology and Evolution of Parasites, Institute of Biology, University of Neuchâtel, Neuchâtel, Switzerland; 2 Laboratory of Ecology and Epidemiology of Parasites, Institute of Biology, University of Neuchâtel, Neuchâtel, Switzerland; 3 Department of Veterinary Microbiology, Western College of Veterinary Medicine, University of Saskatchewan, Saskatoon, Canada

**Keywords:** Beech tree, *Borrelia burgdorferi*, Climate, *Fagus sylvaticus*, *Ixodes ricinus*, Lyme borreliosis, Masting, Tick-borne disease, Tick population ecology

## Abstract

**Background:**

The incidence of Lyme borreliosis and other tick-borne diseases is increasing in Europe and North America. There is currently much interest in identifying the ecological factors that determine the density of infected ticks as this variable determines the risk of Lyme borreliosis to vertebrate hosts, including humans. Lyme borreliosis is caused by the bacterium *Borrelia burgdorferi* sensu lato (s.l.) and in western Europe, the hard tick *Ixodes ricinus* is the most important vector.

**Methods:**

Over a 15-year period (2004–2018), we monitored the monthly abundance of *I. ricinus* ticks (nymphs and adults) and their *B. burgdorferi* s.l. infection status at four different elevations on a mountain in western Switzerland. We collected climate variables in the field and from nearby weather stations. We obtained data on beech tree seed production (masting) from the literature, as the abundance of *Ixodes* nymphs can increase dramatically 2 years after a masting event. We used generalized linear mixed effects models and AIC-based model selection to identify the ecological factors that influence inter-annual variation in the nymphal infection prevalence (NIP) and the density of infected nymphs (DIN).

**Results:**

We found that the NIP decreased by 78% over the study period. Inter-annual variation in the NIP was explained by the mean precipitation in the present year, and the duration that the DNA extraction was stored in the freezer prior to pathogen detection. The DIN decreased over the study period at all four elevation sites, and the decrease was significant at the top elevation. Inter-annual variation in the DIN was best explained by elevation site, year, beech tree masting index 2 years prior and the mean relative humidity in the present year. This is the first study in Europe to demonstrate that seed production by deciduous trees influences the density of nymphs infected with *B. burgdorferi* s.l. and hence the risk of Lyme borreliosis.

**Conclusions:**

Public health officials in Europe should be aware that masting by deciduous trees is an important predictor of the risk of Lyme borreliosis.
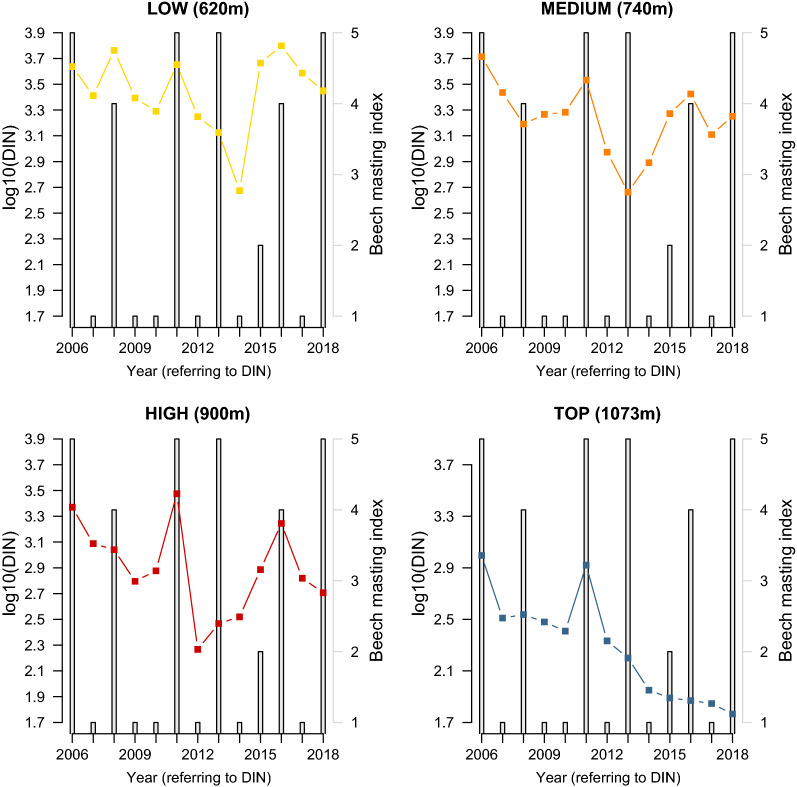

## Background

Ticks and tick-borne diseases represent a significant health problem for humans and their companion animals [1]. Tick-borne pathogens cause both morbidity and mortality in their vertebrate hosts. A recent report by the US Centers for Disease Control and Prevention (CDC) found that tick-borne diseases in the USA had more than doubled over a period of 13 years (> 22,000 cases in 2004 to > 48,000 cases in 2016) with Lyme borreliosis accounting for 82% of all tick-borne disease reports [2]. The incidence of Lyme borreliosis and other tick-borne diseases is also increasing in Europe and Canada [3–8]. The explanation for this increased incidence of tick-borne disease is multi-factorial [9] and includes climate change [7, 8], changes in human land use [10, 11] and even socio-economic changes [12–14]. To understand the epidemiology of tick-borne diseases, it is critical to identify the ecological factors that influence the density of infected ticks because this variable determines the risk of infection to vertebrate hosts, including humans [15].

Lyme borreliosis is the most common vector-borne disease in the northern hemisphere [16, 17]. The causative agents are spirochete bacteria belonging to the *B. burgdorferi* sensu lato (s.l.). complex, which are transmitted among vertebrate hosts by *Ixodes* ticks. In Europe, the sheep tick (*Ixodes ricinus*) is the main vector that maintains *B. burgdorferi* s.l. in nature [18]. The life cycle of *I. ricinus* involves three active stages: larva, nymph and adult. Blood meals are required for larvae and nymphs to develop to the next stage and for egg production in adult female ticks. Immature *I. ricinus* ticks feed on a large variety of vertebrate hosts [18–20]. Larvae acquire *B. burgdorferi* s.l. after feeding on an infected host, as transovarial transmission of *B. burgdorferi* s.l. is believed to be rare or non-existent [21–23]. These engorged infected larvae moult into and overwinter as infected nymphs, which search for new vertebrate hosts the following spring and can transmit the infection during the nymphal blood meal [24]. In contrast, adult female ticks usually feed on incompetent reservoir hosts, such as deer, and therefore do not contribute directly to the maintenance of Lyme borreliosis in nature [25–27].

The infection risk of Lyme borreliosis to vertebrate hosts is determined by the density of infected nymphs and infected adult female ticks. The density of infected nymphs (DIN) is the most important risk factor because nymphs are more numerous and less noticeable than adult female ticks [10, 15, 28–30]. The DIN describes the probability for a host to acquire the pathogen from an infected nymph in its habitat [31]. In practice, the DIN is often estimated as the product of two other variables, the density of nymphs (DON) and the nymphal infection prevalence (NIP), the latter being the percentage of nymphs infected with *B. burgdorferi* s.l. [15]. The phenomenon of the seasonal risk of Lyme borreliosis is well known because the DON and the DIN vary dramatically over the seasons. The seasonal phenology of *I. ricinus* nymphs differs among geographic locations, but in continental Europe it is bimodal and consists of a large spring peak followed by a smaller autumn peak [32–34]. In contrast, the ecological factors driving inter-annual variation in the risk of Lyme borreliosis are less well studied because it requires long-term studies with good information on the DIN [35, 36]. From a public health perspective, understanding the ecological factors that cause inter-annual variation in the DIN, and hence in the risk of infection, is important for developing control strategies to reduce the incidence of Lyme borreliosis.

The ecological drivers underlying spatiotemporal variation in the DIN are a combination of abiotic (e.g., climate) and biotic factors (e.g., abundance of vertebrate hosts and vegetation). *Ixodes* ticks spend more than 98% of their time off the host [37, 38], and they have to cope with seasonal changes in temperature and precipitation. Their life history traits (development, survival, and reproduction) are highly sensitive to different climate variables. For example, tick development rates and survival rates increase with temperature and relative humidity, respectively [37, 39–41], suggesting that warmer and wetter environments should increase nymphal density and the risk of Lyme borreliosis.

Tick population ecology is also highly sensitive to the abundance of vertebrate hosts because all motile tick stages must blood feed to graduate to the next stage in the life cycle [42, 43]. Immature ticks (larvae and nymphs) feed on small mammals, such as rodents, which often exhibit dramatic inter-annual fluctuations in population size [44, 45]. An important source of food for many rodent species is the fruit (also called mast) of forest trees, and the annual production of this fruit can also vary dramatically among years [46–49]. Studies on *Ixodes scapularis* ticks in North America have shown that masting events increase the abundance of rodents in the following year, which increases larval feeding success, which in turn increases the DON and the DIN 2 years after the masting event [35, 50–52]. In Europe, two recent long-term studies have shown that seed production by trees increased the DON 2 years later [53, 54]. However, direct evidence that natural fluctuations in tree seed production influence the DIN and hence the risk of Lyme borreliosis is still lacking in Europe.

The aim of this study was to better understand the ecological factors that influence the inter-annual variation in the DIN and hence the risk of Lyme borreliosis. We used a long-term study to test whether the NIP and the DIN have changed along an altitudinal gradient in Switzerland. Specifically, we used data from a 15-year study that monitored the monthly abundance of *I. ricinus* nymphs and adult ticks and their *B. burgdorferi* s.l. infection status at four different elevations on Chaumont Mountain, in the canton of Neuchâtel, Switzerland. We had previously analyzed this long-term data set and shown that the DON doubled over time at this study location and that seed production by European beech trees had a strong and positive effect on the DON 2 years later [54]. If the NIP remains constant over time, we expect that the DIN would double over the duration of the study and that beech masting would be important for explaining inter-annual variation in the DIN with the expected 2-year time lag.

## Methods

### Study location

The study was conducted on the south-facing slope of Chaumont Mountain, which is part of the Jura Mountains, and is in the canton of Neuchâtel, in western Switzerland. Four tick sampling sites, referred to as low, medium, high and top, were established at elevations of 620, 740, 900, and 1073 m above sea level (a.s.l.), respectively, and have been previously described [55–57]. There is logging in the area, and there are hiking trails and recreation areas for the public. The forest on Chaumont Mountain is mainly composed of European beech (*Fagus sylvatica*; 28.6%), Norway spruce (*Picea abies*; 28.5%), European silver fir (*Abies alba*; 20.4%), sycamore maple (*Acer pseudoplatanus*; 5.9%), European ash (*Fraxinus excelsior*; 3.7%), Scots pine (*Pinus sylvestris*; 2.3%), sessile oak (*Quercus petraea*; 2.3%), willow (*Salix* ssp.; 2.1%), common whitebeam (*Sorbus aria*; 1.6%), and European hornbeam (*Carpinus betulus*; 1.0%).

### Sampling *I. ricinus* ticks in the field

Questing *I. ricinus* nymphs and adult ticks were collected monthly over a period of 15 years (January 2004 to December 2018) at each of the four elevation sites. The sampling protocol has been described previously [57]. Briefly, a 1-m^2^ cotton flag was dragged across low vegetation over a transect distance of 120 m at the medium, high and top elevation sites; each transect of 120 m consisted of six drags of 20 m. At the low elevation site, the transect distance was 100 m and consisted of five drags of 20 m. The flag was inspected every 20 m, and nymphs and adult ticks were counted separately and placed in collection vials. This method of tick collection targets questing ticks and removes them from the environment, which means that they cannot be encountered on future sampling occasions and they cannot contribute to future tick population growth. The same person (Olivier Rais) conducted all of the 3427 drags (4 elevations  × 15 years × 12 months × 5 or 6 drags = 4140 drags). No dragging was performed on days when there was snow on the ground (hereafter referred to as snow days). Over the study period (15 years × 12 months = 180 sampling dates), a total of 34 snow days occurred, which resulted in missing data for 713 drags.

### Selection of ticks for testing of *B. burgdorferi* s.l. infection

Questing *I. ricinus* nymphs and adult ticks were placed in separate plastic vials (length: 9.5 cm; diameter: 1.6 cm) containing grass collected at the field sites to maintain a high relative humidity. Vials were brought to the laboratory and ticks were kept alive and at room temperature until DNA extraction. Over the 15 years of the study, we collected a total of 41,972 *I. ricinus* ticks at the four elevation sites: 32,823 nymphs and 9149 adult ticks (4658 males and 4491 females). For each combination of elevation site and sampling date (4 elevation sites × 15 years × 12 months = 720 combinations), a maximum of 40 questing *I. ricinus* ticks (20 nymphs, 10 adult females, 10 adult males) was selected for subsequent testing of *B. burgdorferi* s.l. infection.

### DNA extraction of whole ticks

Total DNA was extracted from live *I. ricinus* ticks within 10 days after field collection using ammonium hydroxide (NH_4_OH), as previously described [56]. Briefly, entire ticks were lysed in 100 µl of 0.7 M NH_4_OH solution and boiled at 100 °C for 15 min. After allowing the solution to cool, tubes were opened and boiled again for 15 min to allow the ammonia to evaporate [58]. DNA extractions were stored at − 20 °C until further analysis by reverse line blot (RLB).

### Detection and identification of *B. burgdorferi* s.l. species by PCR and RLB

The DNA extractions of the ticks were analyzed for infection with *B. burgdorferi* s.l. and the identity of the *B. burgdorferi* s.l. genospecies was determined using PCR and RLB, as previously described [56]. Briefly, the variable spacer region between two repeated copies of the *23S rRNA* and *5S rRNA* genes was amplified using a conventional PCR [59]. The RLB allows us to detect and identify the six *B. burgdorferi* s.l. genospecies present at our study site: *B. burgdorferi* sensu stricto (s.s.), *B. afzelii*, *B. garinii*, *B. valaisiana*, *B. bavariensis,* and *B. lusitaniae*, as well as the relapsing fever spirochete *Borrelia miyamotoi*. The *B. burgdorferi* s.l.-positive PCR products were allowed to hybridize to a Biodyne C membrane (Pall Corp., New York, NY, USA) that contained seven genospecies-specific oligonucleotide probes using a Miniblotter 45 (Immunetics Inc., Boston, MA, USA) [59]. Hybridization was visualized by incubating the membrane with enhanced chemiluminescence detection liquid and by exposing the membrane to X-ray film.

Each RLB can process 45 samples and we performed a total of 325 RLBs to process all the ticks. The RLB blots were validated with DNA from cultures of the six *B. burgdorferi* s.l. genospecies to confirm that the genospecies-specific probes were working properly. These cultures of the six *B. burgdorferi* s.l. genospecies were grown fresh whenever they were needed over the duration of the study. We defined the RLB time lag as the time interval between the date of tick sampling and the RLB (this time lag is similar to the time lag between the DNA extraction and the RLB). There was considerable variation in the RLB time lag among the RLBs (nymphs: mean = 859 days, range = 4–5025 days; adult ticks: mean = 914 days, range = 4–3487 days).

The ability of the RLB to detect *B. burgdorferi* s.l. is comparable to that of other molecular detection methods. In a previous study, we used a sample of field-captured *I. ricinus* nymphs (*n* = 788) to show that there was a strong correlation (*r* = 0.883, *P* < 0.001) between the RLB method and a quantitative PCR that targets the *flagellin* gene of *B. burgdorferi* s.l. [60]. In another study, we used Sanger sequencing of nymph-derived *B. burgdorferi* s.l. isolates (*n* = 110) to show that the RLB identifies the correct genospecies for > 95.0% of the samples [61].

### Field-collected climate variables

Temperature (T; in °C) and relative humidity (RH; in %) were recorded at 60 cm above the ground at 1 moment in time on the day of tick collection (usually between 10:00 a.m. and 2:00 p.m.) at each tick sampling site using a thermohygrometer (model 615; Testo SA, Lonay, Switzerland). Thus, for each combination of elevation and year, we had a total of 12 field-collected measurements of temperature and relative humidity. The saturation deficit (SD) is a measure of the drying power of the atmosphere (in mmHg) and is calculated using temperature and relative humidity as follows: SD =  (1 − RH/100) × 4.9463 ×  *e*^0.0621T^ [62, 63]. The accuracy of our field-collected climate data was confirmed by comparing them to the weather station data [54].

### Weather station climate variables

We also obtained climate data from the Climap-net database of the Federal Office of Meteorology and Climatology MeteoSwiss. Two weather stations close to our study site are in Neuchâtel at 485 m a.s.l. and in Chaumont at 1136 m a.s.l. These weather stations sample, at 200 cm above the ground, the temperature and relative humidity every hour, and the total precipitation each day. We used the data on the daily mean temperature (average of the 24 hourly measurements), the daily mean relative humidity (average of the 24 hourly measurements) and the daily total precipitation. Thus, for each year, we had a total of 365 weather station measurements of these three climate variables. The SD was calculated as previously described. For each of the four elevation sites, we calculated site-specific climate variables by interpolating the data between the two weather stations (Additional file 1: Section 1).

### Data on inter-annual variation in tree masting

We previously demonstrated that the abundance of *I. ricinus* ticks depends on the seed production of deciduous trees [54]. The seeds or fruit of forest trees, such as the acorns of oak trees and the beech nuts of beech trees, are often referred to as mast. The annual production of mast by a population of trees in an area is highly variable among years [64]. The MASTREE database contains data on masting (or seed production) for many locations in Europe from 1982 to 2016 for two tree species, European beech (*Fagus sylvatica*) and Norway spruce (*Picea abies*) [65]. In this database, the mast intensity is classified into five classes, namely 1, 2, 3, 4, and 5, which refer to very poor mast, poor mast, moderate mast, good mast, and full mast, respectively [65]. We used the MASTREE database [65] to obtain masting data for the European beech and Norway spruce for the canton of Neuchâtel for the years of our study. These two species account for 57.1% of the trees at our study location.

### Statistical methods

The years 2004 and 2005 were excluded from the statistical analysis because they had missing data for the RLB time lag and for the field-collected climate variables with a time lag of 1 and 2 years prior to tick collection. For this reason, the statistical analyses in the main manuscript are restricted to a 13-year period (2006–2018).

#### Annual cumulative nymph density (CND) is the annual density of nymphs (DON)

The cumulative nymph density (CND) is an estimate of the total annual abundance of questing nymphs per 100 m^2^ and was estimated by integrating the area under the curve (AUC) of the monthly questing nymph densities for each year [55, 66]. We used this AUC approach because it is less likely to be biased by missing data (i.e., for the snow days) compared to calculating a simple average for each year. The interpretation of the CND is the theoretical number of questing nymphs per 100 m^2^ that would have been collected if we had sampled ticks daily over the course of a year (as estimated from 12 monthly sampling occasions). If the CND is divided by 365, we obtain the mean daily number of nymphs collected per 100 m^2^ (for details, see Additional file 1: Section 2). We assume that the CND represents a small unknown fraction of the density of nymphs that was present in the area. In summary, tick abundance data from 740 monthly transects (and 3,427 individual drags) were collapsed into 60 estimates of CND (15 years × 4 elevations = 60 annual estimates of abundance). The same approach was used to calculate the cumulative adult density (CAD). We previously analyzed the climate variables and ecological variables that influence inter-annual variation in the CND and the CAD at our study location [54]. For consistency with other studies, we will hereafter refer to the CND and the CAD as the DON and the density of adult ticks (DOA), respectively.

#### Annual nymphal infection prevalence (NIP)

Nymphs that tested negative or positive on the RLB were defined as being uninfected or infected with *B. burgdorferi* s.l., respectively. The nymph infection status is a binomial variable (uninfected and infected nymphs were coded as 0 and 1, respectively) that was used to calculate the annual NIP, which is the percentage of nymphs infected with *B. burgdorferi* s.l. for a given combination of elevation site and year. The same approach was used to calculate the adult infection prevalence (AIP).

#### Annual density of infected nymphs (DIN)

The annual DIN is a measure of the total annual abundance of questing infected nymphs per 100 m^2^ and was estimated by multiplying our annual estimates of the DON by our annual estimates of the NIP (separately for each of the four elevation sites). The interpretation of the DIN is the theoretical number of questing infected nymphs per 100 m^2^ that would have been collected if we had sampled ticks daily over the course of a year. The same approach was used to calculate the annual density of infected adults (DIA); we multiplied the DOA by the AIP (separately for each of the four elevation sites).

#### Annual mean climate variables

To investigate the relationship between climate and the NIP and DIN, we collapsed our monthly or daily weather data into a set of annual means. For the field-collected data, the annual means were calculated over the 12 measurements (i.e., a single measurement for each month). For the weather station data, the annual means were calculated over 365 daily means (i.e., a total of 365 days × 24 measurements/day = 8760 hourly measurements). Thus, the weather station annual means were based on 730 times more data than the field-collected annual means. However, an important advantage of the field-collected data was that they were specific for each of the four elevation sites. In contrast, the Climap-net data came from two weather stations that were located at some distance from the four elevation sites. To facilitate comparison between the slopes of the climate variables, we standardized the climate variables to *Z* scores (mean of zero and a standard deviation of 1).

#### Annual tree masting variables

Previous studies [35, 53, 54] have shown that there is a 2-year time lag between masting events and the DON and a 3-year time lag between masting events and the DOA. Our recent analysis of tick abundance at our study location showed that inter-annual variation in the DON and the DOA was strongly associated with the mast scores of European beech trees but not Norway spruce [54]. Taken together, these studies validate our decision to model the DIN and DIA as a function of the European beech mast scores 2 years previously (year *y* − 2) and 3 years previously (year *y* − 3), respectively. For example, we expect that beech mast scores from the year 2001 predict the DIN in year 2003 (2 years later) and the DIA in year 2004 (3 years later). The same approach was used to model the NIP and the AIP.

#### RLB time lag

As mentioned in the molecular methods, there was considerable variation in the RLB time lag (range: 4–5025 days), which is the time interval between the date of tick collection (and tick DNA extraction)* versus* the date of the RLB. The ammonium hydroxide solution used to extract the whole tick DNA is not optimal for long-term DNA storage, and we were concerned that the DNA would degrade over time and that our ability to detect *B. burgdorferi* s.l. would decrease with the duration of the RLB time lag. We therefore included the RLB time lag as an explanatory variable (standardized to a *Z* score) in our statistical analyses. As information on the RLB time lag was missing for the first 2 years of our study (2004 and 2005), we excluded these years from our statistical analyses.

#### Analysis of NIP

The NIP was modeled using generalized linear mixed effects models (GLMMs) with binomial errors. The fixed effects structure included elevation site (4 levels: low, medium, high, top), the covariate year (rescaled as 1, 2, 3, … 15), the covariate beech mast score 2 years prior (range: 1–5), the covariate DIN in the previous year, the covariate RLB time lag, and the mean annual climate variables of temperature, relative humidity, SD and precipitation (standardized to *Z* scores). As time lags are important in tick ecology, we modeled the NIP as a function of the mean climate variables in the present year, the previous year or 2 years prior. As we did not measure the field-collected climate variables in the 2 years prior to the start of our study (e.g., 2002 and 2003), we had to exclude the years 2004 and 2005 from our statistical analysis. The unique identification number for the 720 transects was included as a random factor. We analyzed the NIP at the transect level (*n* = 4 sites*180 transects = 720 transects) rather than at the year level (*n* = 4 sites*15 years = 60 years) because this approach avoids overdispersion (i.e., by including the transect as a random effect). Overdispersion can be handled by introducing a quasibinomial error function, but this solution cannot be combined with Akaike information criterion (AIC)-based model selection, which is our preferred method for identifying the best model. The same approach was used to model the AIP. All the acronyms of the variables can be found in Table 1.

**Table 1:** Acronyms and definitions of the variables used in the present study

Acronym	Description
DIN	Annual density of infected *I. ricinus* nymphs per 100 m^2^
NIP	Annual nymphal infection prevalence (% of *I. ricinus* nymphs infected with *B. burgdorferi* s.l.)
S	Site name (categorical factor with 4 levels: low, medium, high, top)
Y	Year of the study (covariate: 1, 2, …, 15)
B	Beech mast score in year *y* − 2 (covariate: 1, 2, 3, 4, 5)
RLB	Time lag between tick sampling and the RLB procedure (days)
DIN_*y*−1_	Annual density of infected nymphs in year *y* − 1 per 100 m^2^
T1	Mean temperature in year *y* from the weather station data (°C)
T1_*y*−1_	Mean temperature in year *y* − 1 from the weather station data (°C)
T1_*y*−2_	Mean temperature in year *y* − 2 from the weather station data (°C)
RH1	Mean relative humidity in year *y* from the weather station data (%)
RH1_*y*−1_	Mean relative humidity in year *y* − 1 from the weather station data (%)
RH1_*y*−2_	Mean relative humidity in year *y* − 2 from the weather station data (%)
SD1	Mean saturation deficit in year *y* from the weather station data (mmHg)
SD1_*y*−1_	Mean saturation deficit in year *y* − 1 from the weather station data (mmHg)
SD1_*y*−2_	Mean saturation deficit in year *y* − 2 from the weather station data (mmHg)
PR1	Mean precipitation in year *y* from the weather station data (mm)
PR1_*y*−1_	Mean precipitation in year *y* − 1 from the weather station data (mm)
PR1_*y*−2_	Mean precipitation in year *y* − 2 from the weather station data (mm)
T2	Mean temperature in year *y* from the field-collected data (°C)
T2_*y*−1_	Mean temperature in year *y* − 1 from the field-collected data (°C)
T2_*y*−2_	Mean temperature in year *y* − 2 from the field-collected data (°C)
RH2	Mean relative humidity in year *y* from the field-collected data (%)
RH2_*y*−1_	Mean relative humidity in year *y* − 1 from the field-collected data (%)
RH2_*y*−2_	Mean relative humidity in year *y* − 2 from the field-collected data (%)
SD2	Mean saturation deficit in year *y* from the field-collected data (mmHg)
SD2_*y*−1_	Mean saturation deficit in year *y* − 1 from the field-collected data (mmHg)
SD2_*y*−2_	Mean saturation deficit in year *y* − 2 from the field-collected data (mmHg)

#### Analysis of the DIN

Count data follow a Poisson distribution and aggregated count data follow a negative binomial distribution. In our previous analysis of the DON, we found that generalized linear models with negative binomial errors gave the same results as linear models with normal errors [54]. The reason for this is because our estimates of the DON and the DIN (or DOA and DIA) are summary statistics of counts (integrals based on the counts of 12 monthly transects), which follow a normal distribution according to the central limit theorem of statistics. For simplicity, we therefore assumed that the residuals of our DIN values follow a normal distribution; these values were log10-transformed to further improve their fit to the normal distribution. In summary, the log10-transformed DIN values were analyzed using linear models (LMs) using the same explanatory variables as the NIP. The same approach was used to model the DIA. All the acronyms of the variables can be found in Table 1.

#### AIC-based model selection approach

To identify the best model, we used a model selection approach based on the AIC. Models were ranked according to their AIC values and the Akaike weights, which indicate the percentage support, were calculated for each model. We used the Akaike weights to calculate the model-averaged parameter estimates and their 95% confidence intervals (CIs). For the GLMMs that analyzed the NIP and AIP, we assessed the goodness of fit of the binomial distribution for the best model from the model selection table. For the LMs that analyzed the DIN and DIA, the assumptions of normally distributed residuals and equal variances were assessed for the best model from the model selection table (Additional file 1: Section 3).

We used R version 4.0.3 for all statistical analyses [67]. We used the *lm()* function in the base package to run the LMs with normal errors. We used the *glmer()* function in the lme4 package to run the GLMMs with binomial errors. We used the *mod.sel()* function and the *model.av()* function in the MuMIn package to create the model selection tables and the model-averaged parameter estimates. The raw data used for these statistical analyses can be found in Additional file 2: Table S1.

## Results

### Overview of the statistical analyses

For brevity, we present the analyses of the NIP and DIN. The analyses of the AIP and the DIA are presented in Additional file 1: Section 4.

### Prevalence of *B. burgdorferi *s.l. infection in *I. ricinus*

Over the 15 years of the study and at the four elevation sites, we tested a total of 13,076 *I. ricinus* ticks for infection with *B. burgdorferi* s.l.: 7940 nymphs and 5136 adult ticks (2572 males and 2564 females). The infection prevalence of *B. burgdorferi* s.l. was 15.1% for all ticks (1975/13,076), 12.8% for nymphs (1014/7940) and 18.8% for adult ticks (964/5136), with a similar infection prevalence between males (18.3%; 471/2572) and females (19.1%; 490/2564). The *B. burgdorferi* s.l. genospecies detected in this study (ranked from most common to least common) were: *B. afzelii* (35.6%; 703/1975), *B. garinii* (26.0%; 513/1975), *B. valaisiana* (13.5%; 267/1975), *B. burgdorferi* s.s. (11.0%; 218/1975), *B. bavariensis* (1.2%; 23/1975) and *B. lusitaniae* (0.2%; 3/1975). Another 108 ticks were infected with *B. miyamotoi* (5.5%; 108/1975) while 109 *Borrelia* infections (5.5%; 109/1975) could not be identified to genospecies.

Mixed infections with two or three *Borrelia* genospecies were detected in 6.8% and 0.2% of the infected ticks tested in this study, respectively. Ranked from most common to least common, the *Borrelia* genospecies mixed infections were: *B. garinii* and *B. valaisiana* (3.3%; 65/1975), *B. afzelii* and *B. burgdorferi* s.s. (2.3%; 46/1975), *B. afzelii* and *B. garinii* (0.7%; 13/1975), *B. garinii* and *B. burgdorferi* s.s. (0.2%; 4/1975), *B. afzelii* and *B. valaisiana* (0.1%; 2/1975), *B. afzelii*, *B. burgdorferi* s.s., and *B. miyamotoi* (0.1%; 2/1975), *B. afzelii* and undefined genospecies (< 0.1%; 1/1975), *B. afzelii*, *B. burgdorferi* s.s. and *B. garinii* (< 0.1%; 1/1975), *B. afzelii*, *B. burgdorferi* s.s. and *B. valaisiana* (< 0.1%; 1/1975), *B. bavariensis* and *B. valaisiana* (< 0.1%; 1/1975), *B. bavariensis* and *B. lusitaniae* (< 0.1%; 1/1975) and *B. burgdorferi* s.s. and *B. valaisiana* (< 0.1%; 1/1975).

### Mean NIP at each of the four elevation sites

**Fig. 1:**
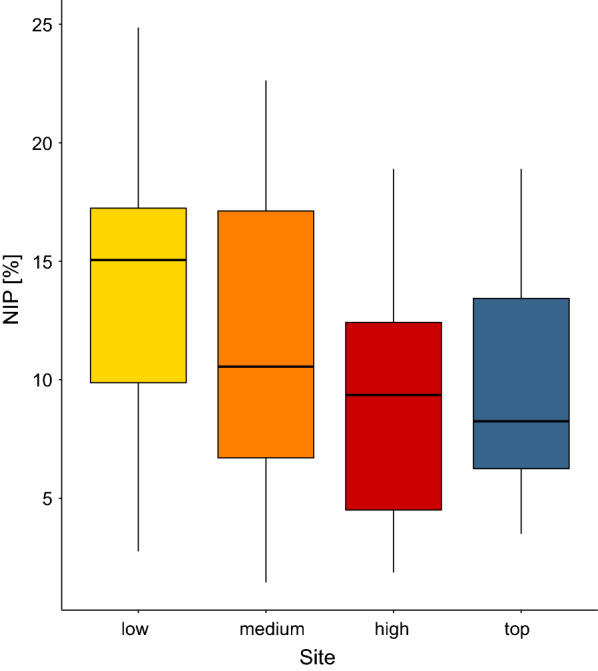
Boxplot of the effect of elevation site on the nymphal infection prevalence (*NIP*), which is the percentage of *I. ricinus* nymphs infected with *B. burgdorferi* s.l.. For each of the four elevation sites, the NIP values are shown for the 15 years of the study (2004–2018). Compared to the low elevation site, the NIP was 13.4%, 23.6%, and 9.4% lower at the medium, high, and top elevation sites, respectively. The boxplots show the median (black line), 25th and 75th percentiles (edges of the box), and minimum and maximum values (whiskers)

The mean NIP for the four elevation sites ranged from 10.8% to 15.3% and are shown in Fig. 1 and Table 2. Importantly, these mean estimates of the NIP do not consider the effects of any other explanatory variables.

**Table 2:** Mean NIP and mean DIN for the four elevation sites on Chaumont Mountain

Site	NIP^a^ (%)	NIP 95% CI	DIN1^b^	DIN1 95% CI	DIN2^b^	DIN2 95% CI
Low	15.3	15.0–15.6	2969	1882–4684	8.1	5.2–12.8
Medium	11.9	11.6–12.2	1775	1125–2800	4.9	3.1–7.7
High	10.8	10.4–11.1	983	623–1552	2.7	1.7–4.3
Top	12.7	12.3–13.1	246	156–388	0.7	0.4–1.1

Values in Table 2 are presented as the means and their 95% confidence intervals (CIs). These means and 95% CIs were calculated from linear models that only contained the explanatory factor of elevation site.

^a^The NIP is the percentage of *I. ricinus* nymphs infected with *B. burgdorferi* s.l..

^b^ The DIN is the number of questing *B. burgdorferi* s.l.-infected *I. ricinus* nymphs per 100 m^2^. The DIN is given as the total for the whole year (DIN1) or the daily average (DIN2). DIN1 is calculated by multiplying the DON by the NIP. DIN2 is calculated by dividing the DIN1 by 365 days.

### Model selection analysis of the NIP

The full model selection table for all 232 models is presented in Additional file 1: Section 5. For the NIP, the best two models had a combined support of 94.0% (Table 3). These two models each had 47.0% of the support (Weight1 in Table 3) and contained the explanatory variables of year (Y), RLB time lag (RLB), and weather station mean annual precipitation in the present year (PR). The only difference between these two models was that the second model contained elevation site (S), whereas the first model did not (Table 3).

**Table 3:** Model selection results for the generalized linear mixed effects models of the NIP

Rank	Model structure	*df*	logLik	AIC	ΔAIC	Weight1	Weight2
1	NIP ~ *Y* + RLB + PR	5	− 2363.1	4736.2	0.0	47.0	47.0
2	NIP ~ *Y* + RLB + PR + S	8	− 2360.1	4736.2	0.0	47.0	94.0
3	NIP ~ *Y* + RLB + RH1	5	− 2365.4	4740.8	4.6	5.0	99.0

Model selection results are shown for the generalized linear mixed effects models (GLMMs) with binomial errors of the NIP response variable. The explanatory variables were site, year, beech masting index 2 years prior, DIN from the previous year, RLB time lag, and the climate variables obtained from the weather stations and the field. The models are ranked according to their Akaike information criterion (AIC). Of the 232 models in the set, only the 3 top models are shown for which the cumulative support (Weight2) > 99.0%. Shown for each model are the model rank (Rank), model structure (see Table 1 for the acronyms of the explanatory variables), model degrees of freedom (*df*), log-likelihood (logLik), AIC, difference in the AIC value from the top model (ΔAIC), model weight (Weight1), and cumulative model weight (Weight2). The results from the full model selection are shown in Additional file 1: Section 5

For the individual explanatory variables, there was strong support for RLB time lag (100.0%), year (100.0%) and weather station mean annual precipitation in the present year (PR; 93.8%), moderate support for site (48.2%), and low support for weather station mean annual relative humidity in the present year (RH1; 5.7%; Table 4). None of the other explanatory variables had a support > 1.0% (Additional file 1: Section 5).

**Table 4:** Support for the five most important explanatory variables of the NIP

Rank	Explanatory variable of interest	Support (%)
1	RLB	100.0
2	Year	100.0
3	PR	93.8
4	Site	48.2
5	RH1	5.7

The support for the 5 most important explanatory variables is shown from the AIC-based model selection table of the NIP. This support is calculated as the sum of the Akaike weights for all the models in the set that include that particular explanatory variable (see Table 1 for the acronyms of the explanatory variables). Additional file 1: Section 5 shows the results for all the explanatory variables.

### Model-averaged parameter estimates for the NIP

To determine the direction and statistical significance of the explanatory variables on the NIP, we present the model-averaged parameter estimates (and their 95% CIs) on the logit scale (Additional file 1: Section 5). We also back-calculated the effect sizes of the explanatory variables on the NIP on the original scale with respect to the following reference conditions: the site was low elevation, the year was 2006, and the covariates of RLB time lag and weather station precipitation in the present year were set to 0 (i.e., the mean values on the *Z* score scale).

**Fig. 2:**
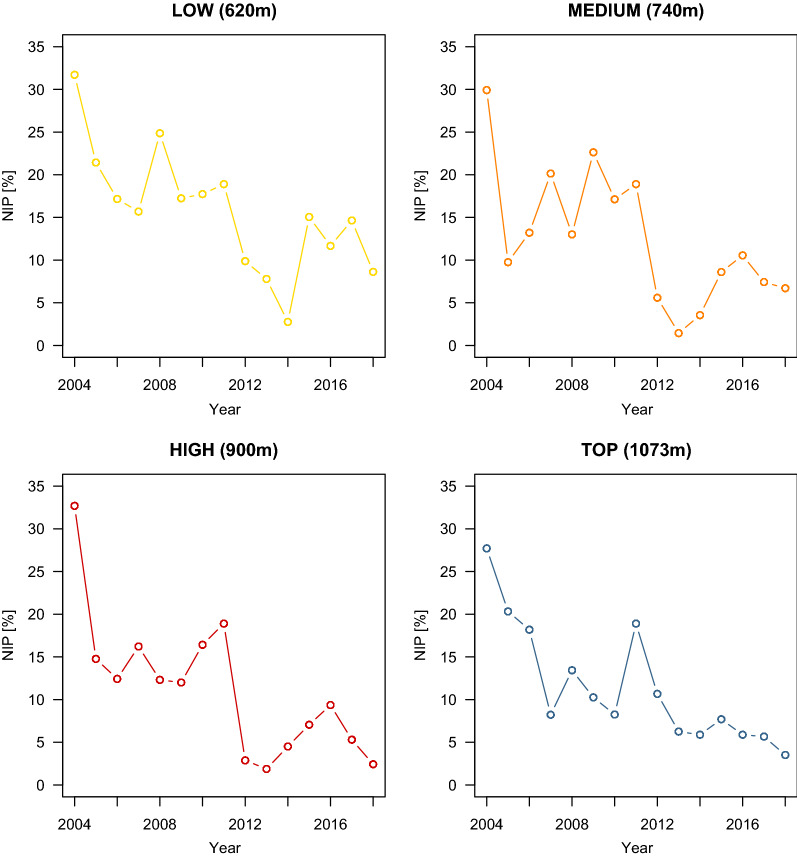
Decrease in the NIP over the 15 years of the study (2004–2018) for each of the four elevation sites on Chaumont Mountain. Each estimate of the NIP is based on a maximum of 240 nymphs (12 months × 20 nymphs per month)

**Fig. 3:**
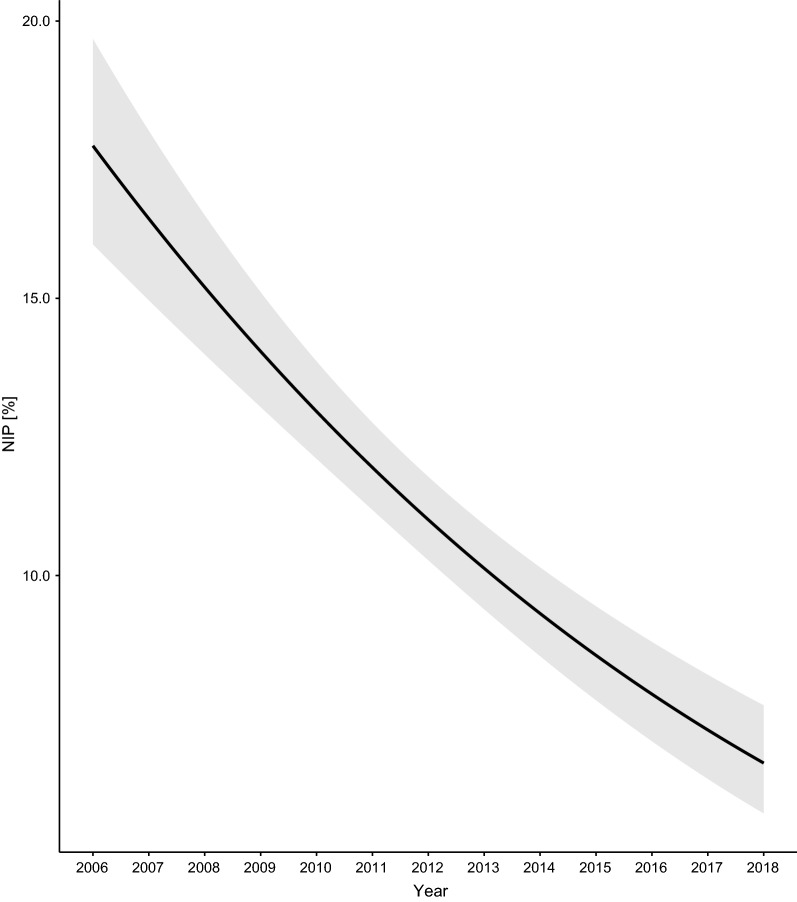
Effect of year on the NIP. The parameter estimates used to calculate the effect sizes were taken from the model-averaged parameter estimates in Additional file 1: Section 5. The NIP decreased by an average of 77.8% at each of the four elevation sites over the 13-year study period

**Fig. 4:**
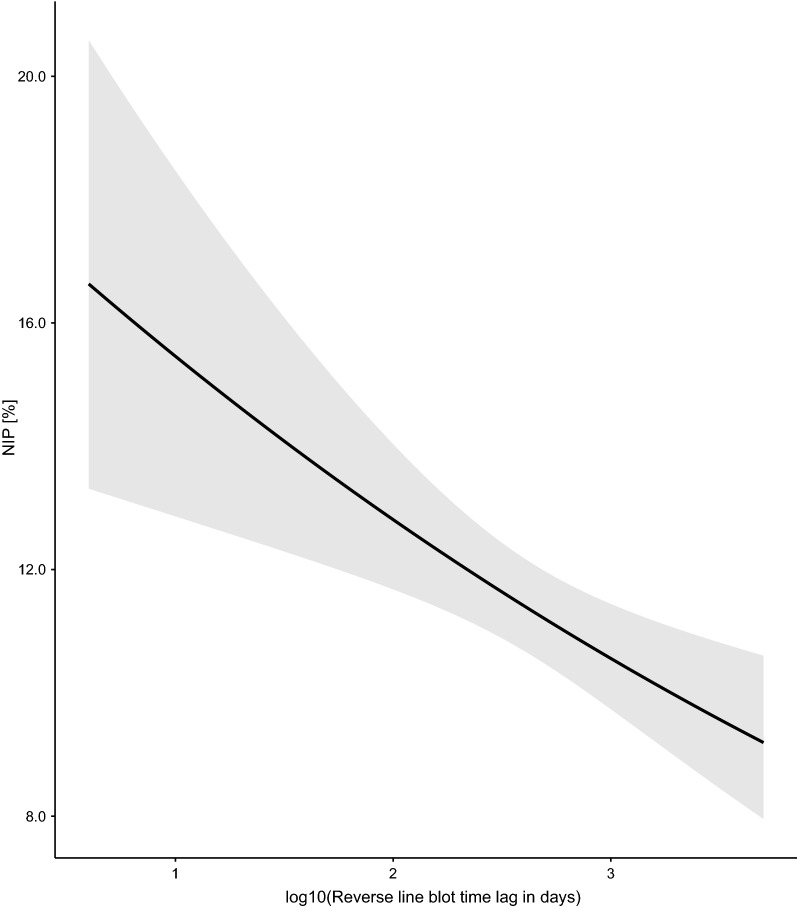
Effect of the reverse line blot (RLB) time lag on the NIP. The RLB time lag is the time interval between the date of collecting the ticks in the field and the date of testing the tick infection status using the RLB. The parameter estimates used to calculate the effect sizes were taken from the model-averaged parameter estimates in Additional file 1: Section 5. Increasing the RLB time lag by one standard deviation (e.g., 881 days) decreased the NIP by an average of 13.1% at each of the four elevation sites

**Fig. 5:**
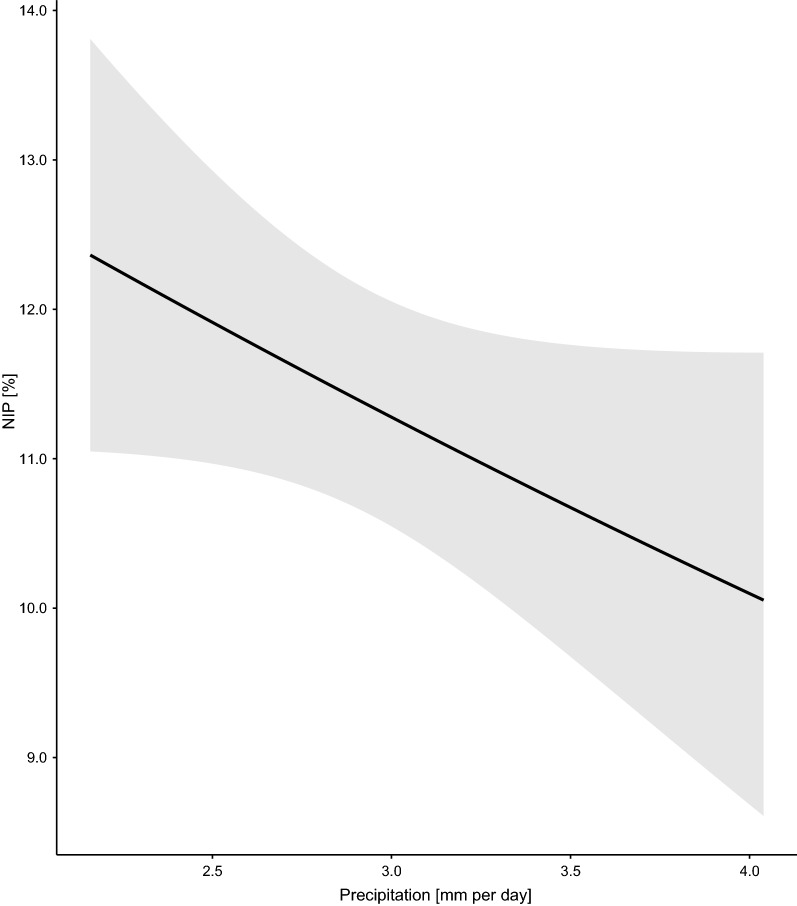
Effect of the mean annual precipitation in the present year on the NIP. The mean annual precipitation (in mm of rainfall per day) was measured by two weather stations near the field site. The parameter estimates used to calculate the effect sizes were taken from the model-averaged parameter estimates in Additional file 1: Section 5. Increasing the precipitation of the present year by one standard deviation (e.g., 0.5 mm of precipitation) decreased the NIP by an average of 23.2% at each of the four elevation sites

The NIP was significantly different between the four elevation sites (Fig. 1; Additional file 1: Section 5). Compared to the low elevation site, the NIP was 13.4% lower at the medium (Medium–Low contrast = −0.195, 95% CI = −0.467 to 0.077), 23.6% lower at the high (High–Low contrast = −0.358, 95% CI = −0.653 to −0.063) and 9.4% lower at the top (Top–Low contrast = −0.135, 95% CI = −0.481 to 0.211) elevation site. Year had a negative and significant effect (slope = −0.145 per year, 95% CI = −0.181 to −0.108), indicating that the NIP was decreasing over time at Chaumont Mountain (Figs. 2, 3). Over the 13-year period of the study (2006–2018), the NIP on the original scale decreased by 77.1% to 78.6% at the four elevation sites (Figs. 2, 3). The RLB time lag had a negative and significant effect on the NIP (Fig. 4; slope = −0.184 per standard deviation, 95% CI = −0.285 to −0.083). Increasing the RLB time lag by one standard deviation (e.g., 881 days) decreased the NIP on the original scale by 12.6%–13.6% at the four elevation sites (Fig. 4). The weather station mean annual precipitation in the present year had a negative and significant effect on the NIP (Fig. 5; slope = −0.311 per standard deviation, 95% CI = −0.447 to −0.174). Increasing the weather station mean annual precipitation in the present year by one standard deviation (e.g., 0.5 mm of precipitation) decreased the NIP on the original scale by 22.4%–24.0% at the four elevation sites (Fig. 5). In summary, the explanatory variables of year, RLB time lag, and precipitation in the same year all had significant negative effects on the NIP.

### Mean DIN at each of the four elevation sites

**Fig. 6:**
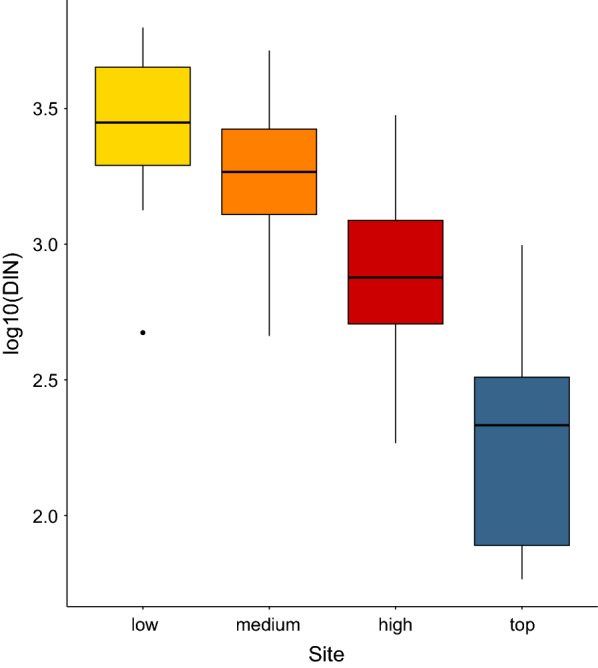
Boxplot of the effect of elevation on the density of infected nymphs (*DIN*). The DIN is the annual estimate of the number of infected questing *I. ricinus* nymphs per 100 m^2^ sampled by the dragging method. For each of the four elevation sites, the DIN values are shown for the 15 years of the study (2004–2018). Compared to the low elevation site, the mean DIN at the medium, high, and top elevation sites were 40.2%, 66.9%, and 91.7% lower, respectively. The boxplots show the median (black line), 25th and 75th percentiles (edges of the box), minimum and maximum values (whiskers), and outliers (solid circles)

The mean DIN was inversely related to the altitudinal gradient; it was highest at the low elevation site and lowest at the top elevation site (Table 2; Fig. 6). If the low elevation site is set as the reference, the mean DIN at the medium, high, and top elevation sites were 40.2%, 66.9%, and 91.7% lower, respectively (Fig. 6). Importantly, these mean estimates of the DIN do not consider the effects of any other explanatory variables.

### Model selection analysis of the DIN

The full model selection analysis with 314 models is presented in Additional file 1: Section 6. For the DIN, the top three models had a combined support of 80.0% (Weight 2 in Table 5). The best model had 47.0% of the support (Table 5), explained 82.5% of the variation in the DIN and contained the explanatory variables of elevation site (S; partial *r*^2^ = 28.3%), year (Y; partial *r*^2^ = 14.8%), site:year interaction (S:Y; partial *r*^2^ = 4.2%), beech mast score 2 years prior (B; partial *r*^2^ = 5.8%), and weather station mean annual relative humidity in the present year (RH1; partial *r*^2^ = 6.1%).

**Table 5:** Model selection results for the linear models of the DIN

Rank	Model structure	*df*	logLik	AIC	ΔAIC	Weight1	Weight2	*r*^2^
1	DIN ~ S + *Y* + *B* + RH1 + S:*Y*	11	8.1	12.3	0.0	47.0	47.0	82.5
2	DIN ~ S + *Y* + RLB + PR_*y*−1_ + S:*Y*	11	7.2	14.2	1.8	19.0	66.0	81.9
3	DIN ~ S + *Y* + *B* + SD1 + S:*Y*	11	6.9	14.8	2.5	14.0	80.0	81.7
4	DIN ~ S + *Y* + *B* + RH1	8	0.7	17.9	5.6	3.0	83.0	78.3
5	DIN ~ S + *Y* + *B* + S:*Y*	11	4.9	18.8	6.5	2.0	85.0	80.2
6	DIN ~ *Y* + *B* + SD1	8	0.2	19.0	6.7	2.0	87.0	77.9
7	DIN ~ S + *Y* + RLB + T1_*y*−1_ + S:*Y*	11	4.6	19.3	7.0	1.0	88.0	80.0
8	DIN ~ S + *Y* + RLB + PR + S:*Y*	11	4.1	20.3	8.0	1.0	89.0	79.6
9	DIN ~ S + *Y* + RLB + RH1_*y*−1_ + S:*Y*	11	4.1	20.4	8.1	1.0	90.0	79.6
10	DIN ~ S + *Y* + RLB + S:*Y*	10	2.3	20.7	8.4	1.0	91.0	78.7
11	DIN ~ S+*Y* + RLB + SD1 + S:*Y*	11	3.9	20.8	8.4	1.0	92.0	79.5
12	DIN ~ S + *Y* + RLB + T1_*y*−2_	11	3.8	21.0	8.7	1.0	93.0	79.4
13	DIN ~ S + *Y* + RLB + S:*Y* + S:RLB	13	7.2	21.1	8.8	1.0	94.0	81.0
14	DIN ~ S + *Y* + RLB + RH1 + S:*Y*	11	3.6	21.3	9.0	1.0	95.0	79.2

Model selection results are shown for the linear models (LMs) with normal errors of the log10-transformed DIN response variable. The explanatory variables were site, year, beech masting index 2 years prior, RLB time lag, and the climate variables obtained from the weather stations and the field. The models are ranked according to their AIC. Of the 314 models in the set, only the 14 top models are shown for which the cumulative support (Weight2) is 95%. Shown for each model are the model rank (Rank), model structure (see Table 1 for the acronyms of the explanatory variables), model degrees of freedom (df), log-likelihood (logLik), AIC, difference in the AIC value from the top model (ΔAIC), model weight (Weight1), cumulative model weight (Weight2), and adjusted *r*-squared value (*r*^2^). The results from the full model selection are shown in Additional file 1: Section 6

The support for the individual explanatory variables was as follows (Table 6): site (100.0%), year (100.0%), site:year interaction (92.0%), beech mast score 2 years prior (69.1%), weather station mean annual relative humidity in the present year (RH1; 50.8%), RLB time lag (31.2%), weather station mean annual precipitation in the previous year (PR_y-1_; 20.0%), and weather station mean annual saturation deficit in the present year (SD1; 16.1%). None of the other explanatory variables had a support > 2.5% (Additional file 1: Section 6).

**Table 6:** Support for the 11 most important explanatory variables of the DIN

Rank	Explanatory variable of interest	Support (%)
1	Site	100.0
2	Year	100.0
3	Site:year	92.0
4	Beech mast score 2 years prior	69.1
5	RH1	50.8
6	RLB	31.2
7	PR_*y*−1_	20.0
8	SD1	16.1
9	T2	2.5
10	T1_*y*−1_	1.7
11	PR	1.2

The support for the 11 most important explanatory variables is shown from the AIC-based model selection table of the DIN. This support is calculated as the sum of the Akaike weights for all the models in the set that include that particular explanatory variable. Additional file 1: Section 6 shows the results for all the explanatory variables

### Model-averaged parameter estimates for the DIN

To determine the effects of the explanatory variables on the DIN, we present the model-averaged parameter estimates on the log10-transformed scale (Additional file 1: Section 6). We also back-calculated the effect sizes of the explanatory variables on the DIN on the original scale with respect to the following reference conditions: the site was low elevation, the year was 2006, the beech mast score 2 years prior was set to 1 and the other covariates were set to 0 (i.e., the mean values on the *Z* score scale).

**Fig. 7:**
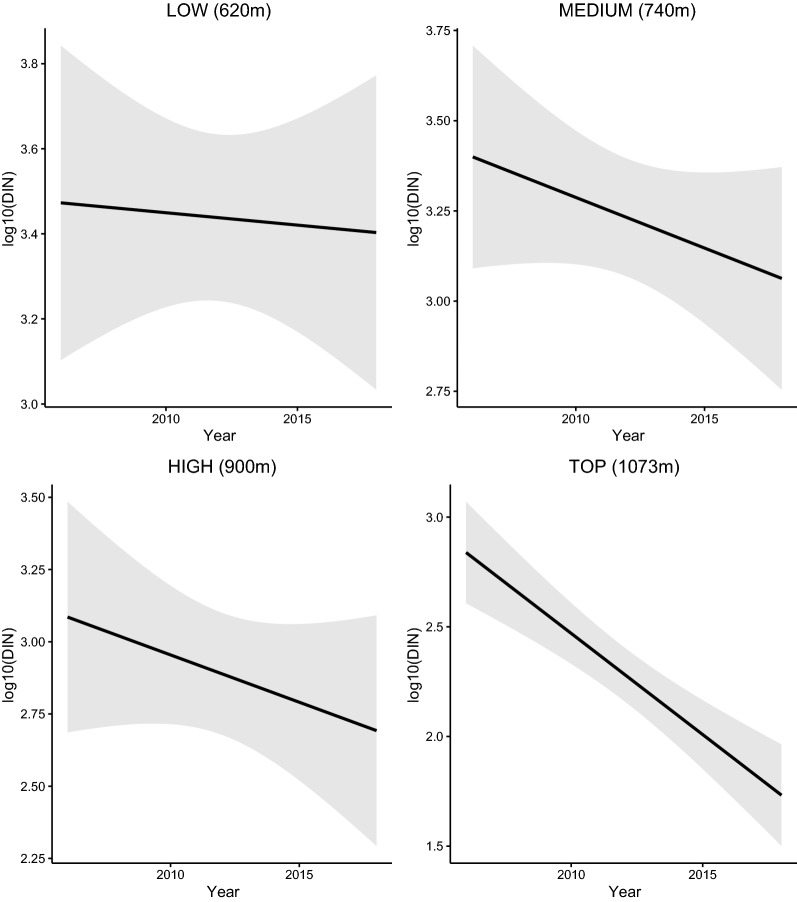
Effect of year on the DIN. The parameter estimates used to calculate the effect sizes were taken from the model-averaged parameter estimates in Additional file 1: Section 6. The DIN decreased by 38.7%, 67.2%, 70.7%, and 94.6% at the low, medium, high, and top elevation sites, respectively, over the 13-year study period

The interaction between site and year indicated that the change in the DIN over time differed between the four elevation sites (Fig. 7; Additional file 1: Section 6). Over the 13-year period (2006–2018), the DIN decreased at the low (slope = −0.018 per year, 95% CI = −0.059 to 0.023), medium (Medium–Low contrast of the slope = −0.023, 95% CI = −0.072 to 0.027), high (High–Low contrast of the slope = −0.027, 95% CI = −0.076 to 0.023) and top (Top–Low contrast of the slope = −0.088, 95% CI = −0.138 to −0.037) elevation site. Over the 13-year period (2006–2018), the DIN decreased by 38.7%, 67.2%, 70.7%, and 94.6% at the low, medium, high, and top elevation sites, respectively (Fig. 7). Due to the significant interaction between site and year, it does not make sense to interpret the differences in intercept between the four elevation sites (Fig. 7; Additional file 1: Section 6).

**Fig. 8:**
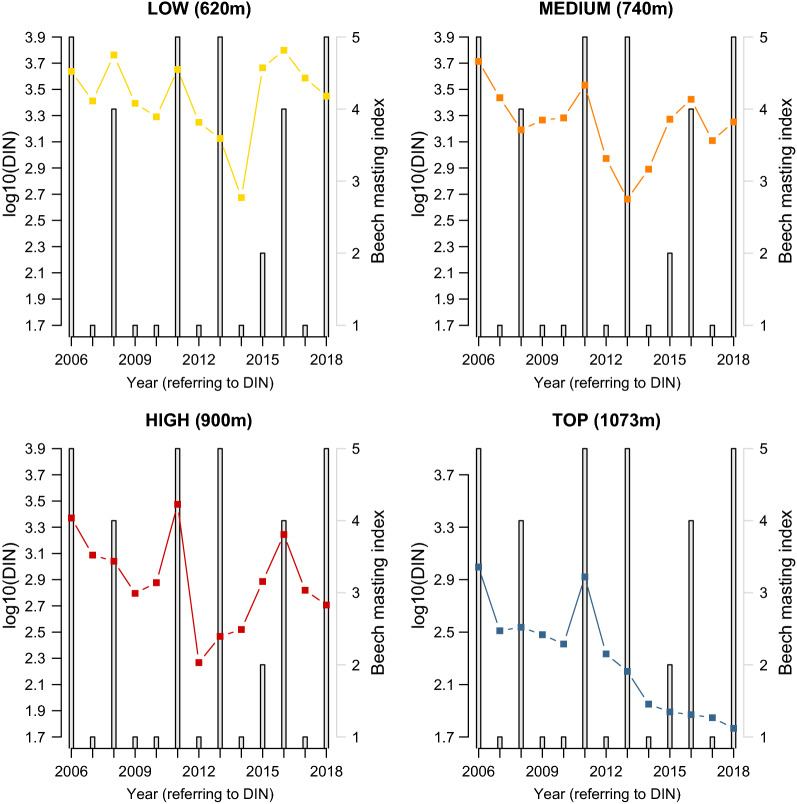
The log10-transformed DIN (square points and solid lines) and the beech tree mast score 2 years prior (open bars) over the 13-year study period for each of the four elevation sites on Chaumont Mountain. The DIN values range from years 2006 to 2018, whereas the beech tree mast scores 2 years prior range from years 2004 to 2016. Years of high seed production by beech trees 2 years prior were associated with high DIN values. Beech tree mast scores of 1, 2, 3, 4, and 5 refer to very poor mast, poor mast, moderate mast, good mast, and full mast, respectively

**Fig. 9:**
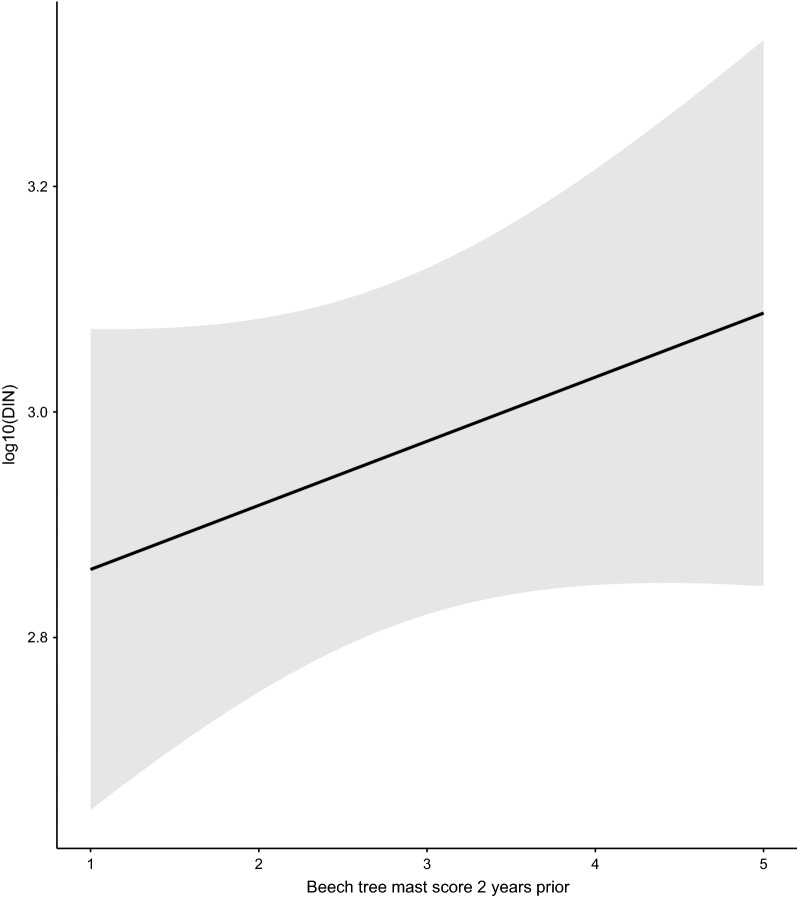
Effect of beech mast score 2 years prior on the DIN. Beech tree mast scores of 1, 2, 3, 4, and 5 refer to very poor mast, poor mast, moderate mast, good mast, and full mast, respectively. The parameter estimates used to calculate the effect sizes were taken from the model-averaged parameter estimates in Additional file 1: Section 6. Increasing the beech tree mast score 2 years prior from 1 (poor mast) to 5 (full mast) increased the DIN by 85.5% at each of the four elevation sites

**Fig. 10:**
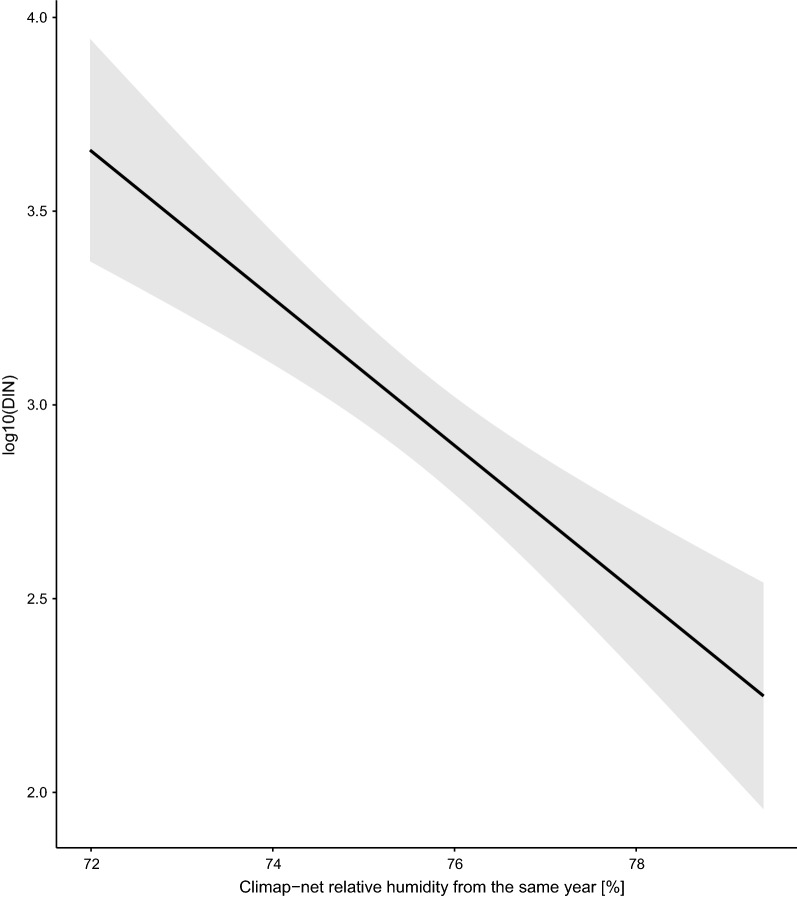
Effect of the mean annual relative humidity in the present year on the DIN. The relative humidity (%) was measured by two weather stations near the field site. The parameter estimates used to calculate the effect sizes were taken from the model-averaged parameter estimates in Additional file 1: Section 6. Increasing the mean annual relative humidity in the present year (i.e., no time lag) by one standard deviation (1.8% of relative humidity) decreased the DIN by 31.8% at each of the four elevation sites

The beech mast score 2 years prior had a positive and significant effect on the DIN (slope = 0.067 per class; 95%CI = 0.029 to 0.105; Figs. 8, 9). Increasing the beech mast score 2 years prior from 1 (poor mast) to 5 (full mast) increased the DIN by 85.5% at each of the four elevation sites on Chaumont Mountain (Fig. 9). The weather station mean annual relative humidity in the present year had a negative and significant effect on the DIN (Fig. 10; slope = −0.166 per standard deviation, 95% CI = − 0.253 to − 0.079). Increasing the weather station mean annual relative humidity in the present year by one standard deviation (1.8% of relative humidity) decreased the DIN by 31.8% at each of the four elevation sites on Chaumont Mountain (Fig. 10).

Other models contained other explanatory variables that had the following effects on the DIN. The RLB time lag had a negative and significant effect on the DIN (slope = −0.131 per standard deviation, 95% CI = −0.215 to −0.047; Additional file 1: Section 6). The weather station mean annual precipitation in the previous year had a negative and significant effect on the DIN (slope = −0.108 per standard deviation, 95% CI = −0.183 to −0.033; Additional file 1: Section 6). The weather station mean annual saturation deficit in the present year had a positive and significant effect on the DIN (slope = 0.205 per standard deviation, 95% CI = 0.079 to 0.330; Additional file 1: Section 6).

In summary, the DIN decreased over time at the four elevation sites and significantly so at the top elevation site. The DIN increased significantly with tree seed production 2 years prior and decreased significantly with the relative humidity in the present year.

## Discussion

Forecasting exposure risk is an important strategy for preventing the spread of tick-borne diseases. In Europe and North America, there is much interest to determine which ecological factors are influencing the abundance of *Ixodes* ticks and their associated pathogens. To address this question, we measured the abundance of *I. ricinus* ticks infected with *B. burgdorferi* s.l. over a period of 15 years in an area of Switzerland where Lyme borreliosis is endemic. The NIP decreased on average by 77.8% over the 13-year study period at the four elevation sites. The inter-annual variation in the NIP was best explained by year, time lag between tick sampling and pathogen detection (the RLB time lag), and the mean annual precipitation in the present year. The DIN decreased over the 13-year study period at all four elevation sites, but the decrease was only significant at the top elevation. The inter-annual variation in the DIN was best explained by site, year, site:year interaction, abundance of beech tree seeds 2 years prior, and the mean annual relative humidity in the present year.

As the DIN is the product of the DON and the NIP, we expect that the factors driving the DON and the NIP should also drive the DIN [31, 35]. Using the data from the present study, we had previously shown that elevation site, year, beech masting 2 years prior, and mean annual relative humidity in the present year were the ecological factors that influenced inter-annual variation in the DON [54], and these same four factors also influenced inter-annual variation in the DIN in the present study. Similarly, year, RLB time lag, and mean annual precipitation in the present year influenced both the NIP and the DIN (although these explanatory variables were not in the top model for the DIN). The explanatory variable of year had a positive effect on the DON, but a negative effect on the NIP, and these two opposing effects resulted in a net negative effect of year on the DIN at the low, medium, and high elevation sites that was not significant. At the top elevation site, the DON, NIP, and DIN all decreased dramatically over the study period. Other explanatory variables, such as beech masting 2 years prior and relative humidity, are important for the DON but not for the NIP, and* vice versa* for the RLB time lag and precipitation. The effects of these explanatory variables on the DIN were therefore reduced.

One of the most important results of this study is our demonstration of a strong and positive association between seed production by beech trees 2 years prior and the DIN. We had previously shown using the same data from the present study that the beech masting index 2 years prior was highly significantly associated with inter-annual variation in the DON [54]. The discovery that masting events can drive inter-annual variation in the DON and DIN of *Ixodes* nymphs with a 2-year time lag was first made in North America [35, 50, 51]. The chain of causality is as follows: mast seeding in year *y* increases the abundance of small mammals, deer, and larval ticks in year *y* + 1 [35, 44, 51, 68–74]. Higher densities of larval ticks feed on higher densities of small mammals in year *y* + 1, which in turn increases the abundance of nymphs in year *y* + 2 [35, 36, 50–52, 75, 76].

In Europe, there is a growing body of evidence that masting events of deciduous trees influence the DIN of *I. ricinus* ticks. A 9-year study in Germany found a 2-year time lag between the masting of European beech trees (*Fagus sylvatica*) and the DON, but did not measure the DIN [53]. A 7-year study in Poland found a 2-year time lag between the masting of oak trees and the incidence of Lyme borreliosis in human patients, but this study did not measure tick abundance [77]. An 18-year study in central Europe found the expected 1-year time lag between rodent densities and the incidence of tick-borne diseases in human patients, but this study did not measure tick abundance or tree seed production [78]. A 3-year study in the Netherlands found a strong correlation between masting in year* y* and rodent densities in year *y* + 1, and between rodent densities in year *y* + 1 and the DIN in year *y* + 2, but this study did not find any correlation between mast seeding in year *y* and the DIN in year *y* + 2 [36]. Thus, our long-term study is the first demonstration in Europe that masting events of deciduous trees are strongly associated with the inter-annual variation of the DIN of *I. ricinus* with the expected 2-year time lag. In summary, seed production by beech trees determines the human risk of Lyme disease 2 years later.

Masting by beech trees did not influence the NIP, and this result is both in agreement and in conflict with previous studies [35, 51]. The effects of masting and rodent density on the NIP are complex and counterintuitive. Theoretical models have shown that the *R*_0_ of tick-borne diseases depends on the ratio of nymphs to hosts [43] and on the aggregation of immature ticks on the same host, which ensures horizontal transmission of *B. burgdorferi* s.l. from infected nymphs to uninfected larvae [42]. In the year following a masting event, the rodent host population is expected to increase dramatically, but the density of infected nymphs that will feed on those rodents was determined by the conditions in the previous year (i.e., the year of the masting event when the rodent density was ‘normal’). Field studies have shown that when the rodent density increases relative to the density of immature ticks, the mean burden of ticks on rodent hosts decreases [75, 79, 80], which reduces horizontal transmission of *B. burgdorferi* s.l. between nymphs and larvae. Thus, masting in year *y* will decrease the ratio of infected nymphs to hosts in year *y* +1 and it will decrease the aggregation of immature ticks on infected hosts in year *y* + 1, and both effects are expected to reduce the NIP in year *y* + 2 when the DON is expected to increase dramatically. However, if the proportional increase in the DON is larger than the proportional decrease in the NIP, the DIN is still expected to increase 2 years after a masting event.

An interesting result is that the mean annual relative humidity had a negative effect on the DIN, which is mediated by the negative effect of this variable on the DON [54]. This result contradicts the general wisdom that survival of immature *Ixodes* ticks increases with relative humidity [17, 37, 81]. However, this result is not without precedent, and other studies in Europe have found a negative relationship between moisture and the abundance of *I. ricinus* nymphs [82–86]. One explanation is that humid environments are favourable for the development of entomopathogenic fungi, which can cause high mortality in *Ixodes* ticks [87, 88]. An alternative explanation is that high levels of rainfall inhibit host-seeking activity or cause flooding that reduces tick survival [17, 35].

An unexpected result was that the mean annual precipitation in the same year had a negative effect on the NIP. Increasing the mean annual precipitation in the present year by one standard deviation (e.g., 0.5 mm of precipitation) decreased the NIP on the original scale by 22.4%–24.0%. Encounters between larval ticks and vertebrate reservoir hosts and acquisition of *B. burgdorferi* s.l. during the larval blood meal are the events that determine whether a questing nymph is infected with *B. burgdorferi* s.l. in the following year. For this reason, it is difficult to imagine how precipitation in the year when the nymphs are captured could influence the NIP. Studies on *Ixodes* nymphs collected in the field have suggested that infection with *B. burgdorferi* s.l. can influence their questing behaviour and survival [89–94]. Thus, possible explanations are that precipitation increases the survival and/or the capture success* via* dragging of uninfected nymphs relative to infected nymphs.

The DIN differed among the four elevation sites (620, 740, 900 and 1073 m a.s.l.) and was inversely related to the altitudinal gradient, which agrees with previous studies [55, 57]. A mechanistic explanation is that the duration of development from one stage to the next is inversely proportional to temperature [40, 95]. At higher and colder elevations, eggs and larvae have much slower development rates than at lower and warmer elevations, which ultimately reduces the number of eggs that reach the nymphal stage [39, 96, 97]. Thus, differences in climate between the four elevation sites are expected to drive variation in the vital rates (development, survival, reproduction), which ultimately determines the observed altitudinal differences in tick density, with the low elevation site having a much higher DIN and DON compared to the top elevation site.

At the top elevation site, the decrease in tick population size was very dramatic. One explanation is the construction of recreation facilities (i.e., mountain bike trails and an adventure park) at approximately  25 m from the top elevation site mid-way through the study. This destruction of the forest habitat and the subsequent increase in the number of human visitors to the top of Chaumont Mountain, as well as the associated disturbance to the wildlife reservoir hosts, may have caused the dramatic decline over time of the *I. ricinus* tick population at the top elevation site. An alternative explanation is that repeated tick sampling over a period of 15 years decreased the tick population at the top site [63]. Field studies typically assume that dragging removes a small fraction of the available tick population, but this assumption may not be true in habitats where tick density is already low.

The RLB time lag had a large negative effect on the NIP and a moderately negative effect on the DIN. We believe that the negative relationship between the RLB time lag and the NIP was caused by experimental error. DNA was extracted by boiling ticks in water containing NH_4_OH, and the resultant DNA extractions were stored in this poor DNA storage solution for an average of 2.4 years (some for as long as 13.8 years) prior to detection of *B. burgdorferi* s.l. using RLB. We believe that the DNA in these crude DNA extractions degraded over time, which decreased the ability of the RLB to detect *B. burgdorferi* s.l. infection in the ticks. The sensitivity of the RLB blots was repeatedly tested over the course of the study by using the DNA from isolates of the six *B. burgdorferi* s.l. genospecies cultured in BSK media as positive controls. However, these isolates were grown fresh from frozen stocks when needed, and the RLB blots were therefore unable to detect the DNA degradation over time in the whole-tick DNA extractions. One solution to check for DNA degradation over time would be to repeatedly test known positive controls that are stored in the same freezers as the study samples over the duration of the study. Another solution is to process all the tick DNA extractions with respect to pathogen detection in a timely manner; for example, within a 1-year window.

One limitation of our study is that we did not collect any data on the density of vertebrate hosts at our study sites. Host blood meal analyses of unfed *I. ricinus* nymphs at our field site have shown that they obtain their larval blood meal from a variety of vertebrate hosts, including rodents, birds, carnivores and ungulates [56]. The community of vertebrate hosts plays a critical role in the ecology of *Ixodes* tick populations and their tick-borne pathogens [20, 98]. The three *Ixodes* tick stages feed on different types of vertebrate hosts; larvae and nymphs feed on small mammals and birds, whereas adult female ticks feed on ungulates [19, 20, 37, 99]. Vertebrate hosts can also differ extensively in their ability to harbor and transmit *B. burgdorferi* s.l. infections to feeding *Ixodes* larval ticks [19, 99–102]. As mentioned previously, field studies on *I. ricinus* in Europe and on *I. scapularis* in North America have shown that the density of rodent reservoir hosts plays a critical role in determining larval feeding success, and hence the DON and the DIN in the following year [35, 36, 50–52, 75, 76]. In the present study, we used the beech masting index as an indirect estimate of the abundance of small vertebrate hosts available to feed the larval ticks the following year. However, data on the vertebrate host community are the missing link in this study and would have undoubtedly enhanced the ability of our models to explain inter-annual variation in the DON, NIP and DIN.

## Conclusions

In conclusion, we found that the NIP decreased by 78% over the study period at Chaumont Mountain. We also found that the DIN decreased over the study period at all four elevation sites, but the decrease was only significant at the top elevation. The RLB time lag had a large negative effect on the NIP and a moderately negative effect on the DIN. Beech masting 2 years prior was strongly and positively correlated with inter-annual variation in the DIN. This is the first long-term study in Europe to provide evidence that seed production by deciduous trees influences the density of nymphs infected with *B. burgdorferi* s.l.. Public health officials in Europe should be aware that masting by deciduous trees is an important predictor of the risk of Lyme borreliosis.

## Supplementary Information

**Additional file 1: Section 1.** Interpolation of the Climap-net climate data. **Section 2.** Correlation between two measures of nymph density. **Section 3.** Assumptions of the statistical methods. **Section 4.** Full statistical analysis of the adult infection prevalence (AIP) and density of infected adults (DIA) for the restricted 13-year period of the study (2006–2018). **Section 5.** Full statistical analysis of the nymphal infection prevalence (NIP) for the restricted 13-year period of the study (2006–2018). **Section 6.** Full statistical analysis of the density of infected nymphs (DIN) for the restricted 13-year period of the study (2006–2018).

**Additional file 2:** **Table S1.** Raw data used for all statistical analyses.

## Data Availability

The raw data for this study are stored in the Additional file 2. The climate data are available from the Climap-net database of the Federal Office for Meteorology and Climatology (http://www.meteosuisse.admin.ch/home/service-et-publications/conseil-et-service/portail-de-donnees-dedie-aux-specialistes.html). The MASTREE database is available in the Ecology - Ecological Society of America repository (http://onlinelibrary.wiley.com/doi/10.1002/ecy.1785/suppinfo).

## Abbreviations

AIC: Akaike information criterion
AIP: Adult infection prevalence
CAD: Cumulative adult density
CND: Cumulative nymph density
DIA: Density of infected adults
DIN: Density of infected nymphs
DOA: Density of adults
DON: Density of nymphs
GLMM: Generalized linear mixed effects model
LM: Linear model
NIP: Nymphal infection prevalence
RH: Relative humidity
RLB: Reverse line blot
SD: Saturation deficit
