# Supplementary material for: Masting by beech trees predicts the risk of Lyme disease
Source: Parasit Vectors. 2021 Mar 20;14:168. doi: 10.1186/s13071-021-04646-0 (PMC7980658; doi:10.1186/s13071-021-04646-0)
Supplement: Supplementary file 1 — Additional file 1: Section 1. Interpolation of the Climap-net climate data. Section 2. Correlation between two measures of nymph density. Section 3. Assumptions of the statistical methods. Section 4. Full statistical analysis of the adult infection prevalence (AIP) and density of infected adults (DIA) for the restricted 13-year period of the study (2006–2018). Section 5. Full statistical analysis of the nymphal infection prevalence (NIP) for the restricted 13-year period of the study (2006–2018). Section 6. Full statistical analysis of the density of infected nymphs (DIN) for the restricted 13-year period of the study (2006–2018). [file 13071_2021_4646_MOESM1_ESM.docx]

**ADDITIONAL FILE 1**

Manuscript title: Masting by beech trees predicts the risk of Lyme disease

Authors: Cindy Bregnard, Olivier Rais, and Maarten J. Voordouw

**Table of Contents**

[Section 1 – Interpolation of the Climap-net climate data 2](#_Toc63931944)

[Section 2 – Correlation between two measures of nymph density 3](#_Toc63931945)

[Section 3 – Assumptions of the statistical methods 5](#_Toc63931946)

[Section 4 – Full statistical analysis of the adult infection prevalence (AIP) and density of infected adults (DIA) for the restricted 13-year period of the study (2006 – 2018) 6](#_Toc63931947)

[Section 5 – Full statistical analysis of the nymphal infection prevalence (NIP) for the restricted 13-year period of the study (2006 – 2018) 31](#_Toc63931948)

[Section 6 – Full statistical analysis of the density of infected nymphs (DIN) for the restricted 13-year period of the study (2006 – 2018) 39](#_Toc63931949)

# Section 1 – Interpolation of the Climap-net climate data

**Methods:** Climap-net data were obtained from two weather stations that are located at 485 m ASL in Neuchâtel and at 1136 m ASL in Chaumont and that are close to our four elevation sites. To create a climate profile that was specific for each of the four elevation sites, we interpolated the values between the two weather stations using the relative elevation distance of each elevation site to the two weather stations. For example, the total elevation distance between the Neuchâtel and Chaumont weather stations is 651 meters, and the elevation distance between the top site and the Neuchâtel weather station is 620 meters, which represents 95.2% of the elevation distance. Thus, the climate at the top site is expected to be more similar to the Chaumont weather station (95.2%) compared to the Neuchâtel weather station (4.8%), whereas the reverse would be true for the low site. For each elevation site, the mean daily temperature, relative humidity, saturation deficit, and precipitation were calculated based on the interpolating percentages (Table S1).

Table S1. Climap-net data interpolation. Shown are the site, elevation, elevation distance with the Neuchâtel weather station (Dist 1), elevation distance between Neuchâtel and Chaumont weather station (Dist 2), the interpolating percentage from the Neuchâtel weather station (Neuchâtel), and the interpolating percentage from the Chaumont weather station (Chaumont).

| **Site** | **Elevation**  **(m ASL)** | **Dist 1**  **(m)** | **Dist 2**  **(m)** | **Neuchâtel** | **Chaumont** |
| --- | --- | --- | --- | --- | --- |
| Top | 1073 | 620 | 651 | 4.80% | 95.20% |
| High | 900 | 447 | 651 | 31.30% | 68.70% |
| Medium | 740 | 287 | 651 | 55.90% | 44.10% |
| Low | 620 | 167 | 651 | 74.30% | 25.70% |

# Section 2 – Correlation between two measures of nymph density

Methods: We can estimate the mean daily density of nymphs (DON) per 100 m^2^ by taking the arithmetic average of the ~12 annual sampling occasions, hereafter referred to as mean daily DON1. Over a 15-year study with 12 sampling occasions per year at 4 elevation sites, it is inevitable that some sampling occasions are missed due to bad weather or other reasons. Depending on whether these missing values are from months with low or high nymphal density, the estimate of the DON will be biased high or low, respectively. To lessen the bias caused by missing data, we estimated the cumulative nymph density (CND), which is an estimate of the total annual abundance of questing nymphs per 100 m^2^. The CND was estimated by integrating the area under the curve (AUC) of the monthly questing nymph densities for each year (Perret et al. 2000, Eisen et al. 2003). The interpretation of the CND is the theoretical number of questing nymphs per 100 m^2^ that would have been collected if we had sampled ticks daily over the course of a year (this assumes that the removal sampling has no effect on the tick density at the site). If the CND is divided by 365 or 366, we obtain an estimate of the mean DON collected per 100 m^2^, hereafter referred to as the mean daily DON2. To validate our use of the mean daily DON2, we compared this value to the mean daily DON1. We used Pearson’s correlation test to show that the mean daily DON1 and the mean daily DON2 were highly correlated (which was expected). We also used a paired samples t-test to determine the difference between the mean daily DON1 and the mean daily DON2.

**Results:** Across all four elevation sites, the mean daily DON1 and the mean daily DON2 were positively correlated (Pearson’s r = 0.971, n = 60, p < 0.001; Figure S1). The mean daily DON2 was 20.9% lower than the mean daily DON1 (mean difference ± standard error: 8.4 ± 3.8 nymphs per 100 m^2^), and this difference was significant (Paired sample t-test: df = 58, t = 6.611, p < 0.001). This result was expected; the mean daily DON1 is biased high because most of the missing data were from the winter months when the nymphal density is lowest at our field site. In contrast, the DON2 integrates the area under the curve over the entire calendar year and therefore includes these winter months with low nymphal density.


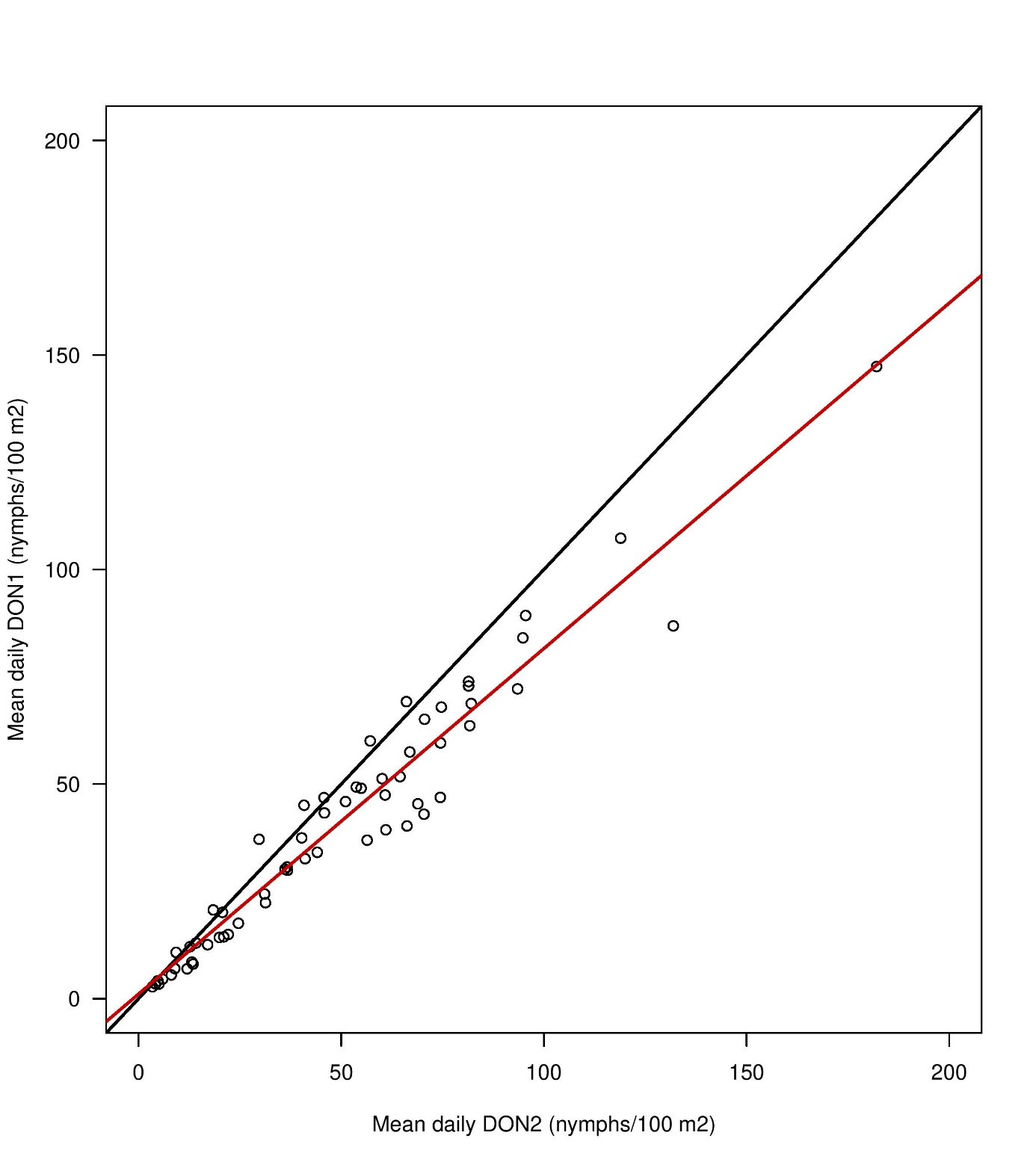
Figure S1. Relationship between the mean daily DON2 (i.e., CND divided by 365 or 366) and the mean daily DON1 (i.e., arithmetic average of the ~12 sampling occasions per year) is shown. The black line represents the 1:1 slope, whereas the red line represents the line of best fit from the linear regression.

# Section 3 – Assumptions of the statistical methods

**Assumptions of the generalized linear mixed effects models (GLMMs):** Generalized linear mixed effects models (GLMMs) with binomial errors assume that the ratio between the residual variance and the residual degree of freedom should be close to 1. For the nymphal infection prevalence (NIP) and the adult infection prevalence (AIP), we examined this ratio for the best model in the AIC-based model selection table.

**Results for the NIP:** For the NIP, the ratio between the residual deviance (4726.2) and the residual degree of freedom (6918.0) of the best model (model 1 in Table S9) was 0.683, indicating that the data were not overdispersed.

**Results for the AIP:** For the AIP, the ratio between the residual deviance (4208.0) and the residual degree of freedom (4565.0) of the best model (model 1 in Table S3) was 0.922, indicating that the data were not overdispersed.

**Assumptions of the linear mixed effects models (LMs):** Linear models assume that the residuals follow a normal distribution and that the variance of the residuals is the same over the range of predicted values (or between groups). We used the Shapiro-Wilk normality test to test the assumption of normality for the residuals of the log10-transformed DIN (or DIA). We used a Bartlett’s K-squared test to test whether the variance of the residuals was the same between the four elevation sites. For the density of infected nymphs (DIN) and the density of infected adults (DIA), we examined these two assumptions for the best model in the AIC-based model selection table.

**Results for the DIN:** For the DIN, the residuals of the best model (model 1 in Table S9) followed a normal distribution (Shapiro-Wilk normality test: W = 0.963, p = 0.110) and the residual variance was not different between the four elevation sites (Bartlett’s K-squared = 1.925, df = 3, p = 0.588).

**Results for the DIA:** For the DIA, the residuals of the best model (model 1 in Table S6) followed a normal distribution (Shapiro-Wilk normality test: W = 0.962, p = 0.096) and the residual variance was not different between the four elevation sites (Bartlett’s K-squared = 2.943, df = 3, p = 0.401).

# Section 4 – Full statistical analysis of the adult infection prevalence (AIP) and density of infected adults (DIA) for the restricted 13-year period of the study (2006 – 2018)

**Adult infection prevalence:** Adults that tested negative or positive on the RLB were defined as being uninfected or infected with *B. burgdorferi* sl, respectively. The adult infection status is a binomial variable (uninfected and infected adults were coded as 0 and 1, respectively) that was used to calculate the annual infection prevalence (AIP), which is the percentage of adults infected with *B. burgdorferi* sl for a given combination of elevation site and year.

Annual density of infected adults: The annual density of infected adults (DIA) is a measure of the total annual abundance of questing infected adults per 100 m^2^ and was estimated by multiplying our annual estimates of the DOA by our annual estimates of the AIP (separately for each of the four elevation sites). The interpretation of the DIA is the theoretical number of questing infected adults per 100 m^2^ that would have been collected if we had sampled ticks daily over the course of a year.

**Annual mean climate variables:** To investigate the relationship between climate and the AIP and DIA, we collapsed our monthly or daily weather data into a set of annual means. For the field-collected data, the annual means were calculated over the 12 measurements (a single measurement for each month). For the weather station data, the annual means were calculated over 365 daily means (i.e., a total of 365 days*24 measurements/day = 8760 hourly measurements). Thus, the weather station annual means were based on 730 times more data than the field-collected annual means. However, an important advantage of the field-collected data was that they were specific for each of the four elevation sites. In contrast, the Climap-net data came from two weather stations that were located at some distance from the four elevation sites. To facilitate comparison between the slopes of the climate variables, we standardized the climate variables to z-scores (mean of 0 and a standard deviation of 1).

**Annual tree masting variables:** Previous studies (Ostfeld et al. 2006, Brugger et al. 2018, Bregnard et al. 2020) have shown that there is a two-year time lag between masting events and the DON and a three-year time lag between masting events and the DOA. Our recent analysis of tick abundance at our study location showed that annual variation in the DON and the DOA was strongly associated with the mast index of European beech trees but not with the mast index of Norway spruce (Bregnard et al. 2020). These studies validate our decision to model the DIN and the DIA as a function of the European beech mast index two years previously (year y-2) and three years previously (year y-3), respectively. For example, we expect that beech mast index from the year 2001 predict the DIN in year 2003 (2 years later) and the DIA in year 2004 (three years later). The same approach was used to model the NIP and the AIP.

**RLB time lag:** As mentioned in the molecular methods of the main manuscript, there was considerable variation in the RLB time lag (range = 4 to 5,025 days), which is the time interval between the date of tick collection (and tick DNA extraction) versus the date of the RLB. The ammonium hydroxide solution used to extract the whole tick DNA is not optimal for long-term DNA storage, and we were concerned that the DNA would degrade over time and that our ability to detect *B. burgdorferi* sl would decrease with the duration of the RBL time lag. We therefore included the RLB time lag as an explanatory variable (standardized to z-score) in our statistical analyses. As the RLB time lag information was missing for the first two years of our study (2004 and 2005), we excluded these years from our statistical analysis.

**Analysis of the AIP:** The AIP was modelled using generalized linear mixed effects models (GLMMs) with binomial errors. The fixed effects structure included elevation site (4 levels: low, medium, high, top), the covariate year (rescaled as 1, 2, 3, … 15), the covariate beech mast index 3 years prior (range: 1 to 5), the covariate RLB time lag, and the mean annual climate variables of temperature, relative humidity, SD, and precipitation. As time lags are important in tick ecology, we modelled the AIP as a function of the mean climate variables in the present year, the previous year, or two years prior. As we did not measure the field-collected climate variables in the two years prior to the start of our study (e.g., 2002 and 2003), we had to exclude the years 2004 and 2005 from our statistical analysis. The unique drag identification number for the 720 combinations of site, year, and month (4*15*12) was included as the random factor. All variable acronyms can be found in Table S2.

**Analysis of the DIA:** Count data follow a Poisson distribution and aggregated count data follow a negative binomial distribution. However, our estimates of the DOA, and by extent the DIA, are summary statistics (sums or integrals) that are based on the counts of ~60 drags (12 dates*5 drags per date). According to the central limit theorem of statistics, summary statistics will follow a normal distribution even if the observations on which they are based are sampled from a non-normal distribution. We therefore assumed that the residuals of our DIA values follow a normal distribution. The annual DIA values were log10-transformed to further improve their fit to the normal distribution. The log10-transformed DIA values (n = 60) were analysed using linear models (LM) with normal errors. The DIA was analysed as a function of the same explanatory variables as the AIP.

**Model selection approach****:** To identify the best model, we used a model selection approach based on the Akaike information criterion (AIC). A big conceptual advantage of model selection is that it reminds the user that there are competing models with different parameter estimates and that some of these models have more support (i.e., are better at explaining the data) than other models. Another advantage is that you can compare non-nested models containing different explanatory variables, which is not possible with a more traditional approach like stepwise multiple regression. Models were ranked according to their AIC values and the Akaike weights, which indicate the percent support, were calculated for each model. We used the Akaike weights to calculate the model-averaged parameter estimates and their 95% confidence intervals (CIs). For the generalized linear mixed effects models that analysed the NIP and AIP, we assessed the goodness of fit of the binomial distribution for the best model from the model selection table. Similarly, for the linear models that analysed the DIN and DIA, the assumptions of normally distributed residuals and equal variances were assessed for the best model from the model selection table (Additional file 1: Section 3). We used R version 4.0.3 for all statistical analyses (Team 2013). We used the lm() function in the base package to run the LMs with normal errors. We used the glmer() functions in the lme4 package to run the GLMMs with binomial errors. We used the mod.sel() function and the model.av() function in the MuMIn package to create the model selection tables and the model-averaged parameter estimates.

**Model selection analysis of the AIP**

The model selection table for the 30 best out of 182 models is presented in Table S3. For the AIP, the best two models had a combined support of 96.0% (Table S3). The first- and second-best models had 76.0% and 20.0% of the support (Table S3), respectively, and contained the explanatory variables of beech mast score 3 years prior, RLB time lag, and field-collected mean annual relative humidity from 2 years prior. The only difference between these two models was that the second model contained site, whereas the first model did not (Table S3).

For the individual explanatory variables, there was strong support for RLB time lag (99.8%), beech mast score 3 years prior year (99.3%), and field-collected mean annual relative humidity from 2 years prior (95.7%), and moderate support for site (20.5%; Table S4). None of the other explanatory variables had a support > 1.4% (Table S4).

**Model-averaged parameter estimates for the AIP**

To determine the direction and statistical significance of the explanatory variables on the AIP, we present the model-averaged parameter estimates (and their 95% confidence intervals) on the logit scale (Table S5). We also back-calculated the effect sizes of the explanatory variables on the AIP on the original scale with respect to the following baseline: the site was low elevation, the beech mast score 3 years prior was set to 1, and the covariates of RLB time lag and field-collected mean annual relative humidity from 2 years prior were set to 0 (i.e., the mean values on the z-score scale).

The AIP was not significantly different between the four elevation sites (Figure S2, Table S5).

The beech mast score 3 years prior had a negative and significant effect on the AIP (Figures S3; slope = -0.202 per class; 95% CI = -0.280 – -0.123). Increasing the beech mast score 3 years prior from 1 (poor mast) to 5 (full mast) decreased the AIP by 34.9% to 48.9% at the four elevation sites on Chaumont Mountain (Figure S3).

The RLB time lag had a positive effect on the NIP (Figure S4; slope = 0.054 per standard deviation, 95% CI = -0.063 – 0.170). Increasing the RLB time lag by one standard deviation (e.g., 925 days) increased the AIP on the original scale by 4.2% to 28.1% at the four elevation sites (Figure S4).

The field-collected mean annual relative humidity 2 years prior had a negative and significant effect on the AIP (Figure S5; slope = -0.311 per standard deviation, 95% CI = -0.457 – -0.165). Increasing the field-collected mean annual relative humidity 2 years prior by one standard deviation (e.g., 7.1% of relative humidity) decreased the AIP on the original scale by 2.4% to 22.0% at the four elevation sites (Figure S5).

In summary, the explanatory variables of beech mast score 3 years prior and relative humidity 2 years prior both had significant negative effects on the AIP.

**Model selection analysis of the DIA**

The model selection table for the 30 best out of 314 models is presented in Table S6. For the annual DIA, the top eleven models had a combined support of 80.0% (Table S6). The best model had 35.0% of the support (Table S6), explained 62.7% of the variation in the annual DIA, and contained the explanatory variables of elevation site (partial r^2^ = 31.3%), year (partial r^2^ = 8.0%), site:year interaction (partial r^2^ = 8.6%), RLB time lag (partial r^2^ = 0.8%), and field-collected mean annual relative humidity 2 years prior (partial r^2^ = 29.8%).

The support for the individual explanatory variables was as follows: site (99.9%), year (85.4%), field-collected mean annual relative humidity 2 years prior (74.0%), site:year interaction (69.6%), RLB time lag (63.8%), and beech mast score 3 years prior (37.3%; Table S7). None of the other explanatory variables had a support > 7.0% (Table S7).

**Model-averaged parameter estimates for the DIA**

To determine the effects of the explanatory variables on the DIA, we present the model-averaged parameter estimates on the log10-transformed scale. We also back-calculated the effect sizes of the explanatory variables on the DIA on the original scale with respect to the following baseline: the site was low elevation, the year was 2006, the beech mast score was set to 1, and the other covariates were set to 0 (i.e., the mean values on the z-score scale).

The interaction between site and year indicated that the change in the DIA over time differed between the four elevation sites (Figure S7, Table S8). Over the 13-year period (2006 – 2018), the DIA increased at the low (slope = 0.017 per year, 95% CI = -0.038 – 0.072), but decreased at the medium (Medium – Low contrast of the slope = -0.011, 95% CI = -0.064 – 0.042), high (High – Low contrast of the slope = -0.058, 95% CI = -0.111 – -0.004), and top (Top – Low contrast of the slope = -0.079, 95% CI = -0.133 – -0.026) elevation sites. Over the 13-year period (2006 – 2018), the DIA increased by 60.1% at the low elevation site, but decreased by 17.1%, 67.5%, and 82.1% at the medium, high, and top elevation sites, respectively (Figure S7). Due to the significant interaction between site and year, it does not make sense to interpret the differences in intercept between the four elevation sites (Table S8).

The RLB time lag had a positive effect on the DIA (slope = 0.047 95% CI = -0.054 – 0.148). Increasing the RLB time lag by one standard deviation (e.g., 925 days) increased the DIA on the original scale by 11.4% at each of the four elevation sites (Figure S8).

The field-collected mean annual relative humidity 2 years prior had a negative and significant effect on the DIA (slope = -0.173 per standard deviation, 95% CI = -0.289 – -0.057). Increasing the field-collected mean annual relative humidity 2 years prior by one standard deviation (e.g., 7.0% of relative humidity) decreased the DIA on the original scale by 32.9% at each of the four elevation sites (Figure S9).

In summary, the DIA remained stable over time at the two lower elevations but decreased significantly at the high and top elevations. The DIA increased with RLB time lag and decreased significantly with the field-collected relative humidity 2 years prior.

Table S2. Acronyms and definitions of the variables used in the main manuscript and additional file 1

| **Acronym** | **Description** |
| --- | --- |
| DIN | Annual density of infected nymphs per 100 m^2^ |
| NIP | Annual nymphal infection prevalence |
| DIA | Annual density of infected adults per 100 m^2^ |
| AIP | Annual adult infection prevalence |
| S | Site name (factor with 4 levels: low, medium, high, top) |
| Y | Year of the study (covariate: 1, 2, …, 15) |
| B | Beech mast score in year y-2 (covariate: 1, 2, 3, 4, 5) |
| B3 | Beech mast score in year y-3 (covariate: 1, 2, 3, 4, 5) |
| RLB | Time lag between tick sampling and RLB procedure (days) |
| DIN_y-1_ | Annual density of infected nymphs in year y-1 per 100 m^2^ |
| T1 | Mean temperature in year y from the weather station data (°C) |
| T1_y-1_ | Mean temperature in year y-1 from the weather station data (°C) |
| T1_y-2_ | Mean temperature in year y-2 from the weather station data (°C) |
| RH1 | Mean relative humidity in year y from the weather station data (%) |
| RH1_y-1_ | Mean relative humidity in year y-1 from the weather station data (%) |
| RH1_y-2_ | Mean relative humidity in year y-2 from the weather station data (%) |
| SD1 | Mean saturation deficit in year y from the weather station data (mmHg) |
| SD1_y-1_ | Mean saturation deficit in year y-1 from the weather station data (mmHg) |
| SD1_y-2_ | Mean saturation deficit in year y-2 from the weather station data (mmHg) |
| PR1 | Mean precipitation in year y from the weather station data (mm) |
| PR1_y-1_ | Mean precipitation in year y-1 from the weather station data (mm) |
| PR1_y-2_ | Mean precipitation in year y-2 from the weather station data (mm) |
| T2 | Mean temperature in year y from the field-collected data (°C) |
| T2_y-1_ | Mean temperature in year y-1 from the field-collected data (°C) |
| T2_y-2_ | Mean temperature in year y-2 from the field-collected data (°C) |
| RH2 | Mean relative humidity in year y from the field-collected data (%) |
| RH2_y-1_ | Mean relative humidity in year y-1 from the field-collected data (%) |
| RH2_y-2_ | Mean relative humidity in year y-2 from the field-collected data (%) |
| SD2 | Mean saturation deficit in year y from the field-collected data (mmHg) |
| SD2_y-1_ | Mean saturation deficit in year y-1 from the field-collected data (mmHg) |
| SD2_y-2_ | Mean saturation deficit in year y-2 from the field-collected data (mmHg) |

Table S3. Model selection results are shown for the generalized linear mixed effects model with binomial errors of the adult infection prevalence (AIP) response variable. The explanatory variables were site, year, beech masting index 3 years prior, RLB time lag, and the climate variables obtained from the weather stations and collected from the field. The models are ranked according to their Akaike Information Criterion (AIC). Shown for each model are the model rank (Rank), model structure (see below for explanation of explanatory variables), model degrees of freedom (Df), log-likelihood (logLik), Akaike information criterion (AIC), difference in the AIC value from the top model (ΔAIC), model weight (Weight1), and cumulative weight (Weight2).

| **Rank** | **Model structure** | **Df** | **logLik** | **AIC** | **ΔAIC** | **Weight1** | **Weight2** |
| --- | --- | --- | --- | --- | --- | --- | --- |
| 1 | AIP ~ B3+RLB+RH2_y-2_ | 5 | -2104.0 | 4218.0 | 0.0 | 76.0 | 76.0 |
| 2 | AIP ~ S+B3+RLB+RH2_y-2_ | 8 | -2102.3 | 4220.7 | 2.7 | 20.0 | 96.0 |
| 3 | AIP ~ B3+RLB+SD2_y-2_ | 5 | -2108.3 | 4226.7 | 8.7 | 1.0 | 97.0 |
| 4 | AIP ~ Y+B3+RLB | 5 | -2108.5 | 4227.1 | 9.1 | 1.0 | 98.0 |
| 5 | AIP ~ Y+RLB+PR | 5 | -2109.1 | 4228.3 | 10.3 | 0.0 | 100.0 |
| 6 | AIP ~ B3+RLB+SD2_y-2_ | 8 | -2106.3 | 4228.7 | 10.7 | 0.0 | 100.0 |
| 7 | AIP ~ B3+RLB+PR_y-2_ | 5 | -2109.7 | 4229.4 | 11.4 | 0.0 | 100.0 |
| 8 | AIP ~ B3 | 3 | -2112.2 | 4230.4 | 12.4 | 0.0 | 100.0 |
| 9 | AIP ~ B3+RLB+T2 | 5 | -2110.6 | 4231.3 | 13.3 | 0.0 | 100.0 |
| 10 | AIP ~ S+Y+B3+RLB | 8 | -2107.8 | 4231.6 | 13.6 | 0.0 | 100.0 |
| 11 | AIP ~ B3+RLB+RH1 | 5 | -2110.8 | 4231.7 | 13.7 | 0.0 | 100.0 |
| 12 | AIP ~ B3+RLB | 4 | -2112.1 | 4232.2 | 14.2 | 0.0 | 100.0 |
| 13 | AIP ~ B3+RLB+PR_y-1_ | 5 | -2111.1 | 4232.3 | 14.3 | 0.0 | 100.0 |
| 14 | AIP ~ B3+RLB+PR | 5 | -2111.4 | 4232.9 | 14.9 | 0.0 | 100.0 |
| 15 | AIP ~ B3+RLB+RH2 | 5 | -2111.4 | 4232.9 | 14.9 | 0.0 | 100.0 |
| 16 | AIP ~ S+Y+RLB+PR | 8 | -2108.4 | 4232.9 | 14.9 | 0.0 | 100.0 |
| 17 | AIP ~ B3+RLB+T2_y-2_ | 5 | -2111.5 | 4232.9 | 14.9 | 0.0 | 100.0 |
| 18 | AIP ~ B3+RLB+RH1_y-1_ | 5 | -2111.5 | 4232.9 | 14.9 | 0.0 | 100.0 |
| 19 | AIP ~ S+B+PR_y-2_ | 8 | -2108.5 | 4233.0 | 15.0 | 0.0 | 100.0 |
| 20 | AIP ~ B3+RLB+T2_y-1_ | 5 | -2111.7 | 4233.5 | 15.5 | 0.0 | 100.0 |
| 21 | AIP ~ B3+RLB+RH2_y-1_ | 5 | -2111.7 | 4233.5 | 15.5 | 0.0 | 100.0 |
| 22 | AIP ~ B3+RLB+SD2_y-1_ | 5 | -2111.8 | 4233.6 | 15.7 | 0.0 | 100.0 |
| 23 | AIP ~ B3+RLB+SD1 | 5 | -2111.8 | 4233.7 | 15.7 | 0.0 | 100.0 |
| 24 | AIP ~ B3+RLB+RH1_y-2_ | 5 | -2111.9 | 4233.7 | 15.7 | 0.0 | 100.0 |
| 25 | AIP ~ B3+RLB+SD1_y-2_ | 5 | -2111.9 | 4233.9 | 15.9 | 0.0 | 100.0 |
| 26 | AIP ~ B3+RLB+T1 | 5 | -2112.0 | 4234.1 | 16.1 | 0.0 | 100.0 |
| 27 | AIP ~ B3+RLB+T1_y-2_ | 5 | -2112.0 | 4234.1 | 16.1 | 0.0 | 100.0 |
| 28 | AIP ~ B3+RLB+T1_y-1_ | 5 | -2112.0 | 4234.1 | 16.1 | 0.0 | 100.0 |
| 29 | AIP ~ B3+RLB+SD1_y-1_ | 5 | -2112.1 | 4234.2 | 16.2 | 0.0 | 100.0 |
| 30 | AIP ~ B3+RLB+SD2 | 5 | -2112.1 | 4234.2 | 16.2 | 0.0 | 100.0 |

Table S4. The support for each explanatory variable is shown from the AIC-based model selection table of the adult infection prevalence. This support is calculated as the sum of the Akaike weights for all the models in the set that include that explanatory variable.

| **Rank** | **Explanatory variable of interest** | **Support (%)** |
| --- | --- | --- |
| 1 | RLB | 99.8 |
| 2 | Beech_y-3_ | 99.3 |
| 3 | RH2_y-2_ | 95.7 |
| 4 | Site | 20.5 |
| 5 | Year | 1.4 |
| 6 | SD2_y-2_ | 1.3 |
| 7 | T1 | < 1.0 |
| 8 | RH1 | < 1.0 |
| 9 | SD1 | < 1.0 |
| 10 | PR | < 1.0 |
| 11 | T1_y-1_ | < 1.0 |
| 12 | RH1_y-1_ | < 1.0 |
| 13 | SD1_y-1_ | < 1.0 |
| 14 | PR_y-1_ | < 1.0 |
| 15 | T1_y-2_ | < 1.0 |
| 16 | RH1_y-2_ | < 1.0 |
| 17 | SD1_y-2_ | < 1.0 |
| 18 | PR_y-2_ | < 1.0 |
| 19 | T2 | < 1.0 |
| 20 | RH2 | < 1.0 |
| 21 | SD2 | < 1.0 |
| 22 | T2_y-1_ | < 1.0 |
| 23 | RH2_y-1_ | < 1.0 |
| 24 | SD2_y-1_ | < 1.0 |
| 25 | T2_y-2_ | < 1.0 |
| 26 | Site:Year | < 1.0 |
| 27 | Site:Beech_y-3_ | < 1.0 |
| 28 | Site:RLB | < 1.0 |
| 29 | Site:T1 | < 1.0 |
| 30 | Site:RH1 | < 1.0 |
| 31 | Site:SD1 | < 1.0 |
| 32 | Site:PR | < 1.0 |
| 33 | Site:T1_y-1_ | < 1.0 |
| 34 | Site:RH1_y-1_ | < 1.0 |
| 35 | Site:SD1_y-1_ | < 1.0 |
| 36 | Site:PR_y-1_ | < 1.0 |
| 37 | Site:T1_y-2_ | < 1.0 |
| 38 | Site:RH1_y-2_ | < 1.0 |
| 39 | Site:SD1_y-2_ | < 1.0 |
| 40 | Site:PR_y-2_ | < 1.0 |
| 41 | Site:T2 | < 1.0 |
| 42 | Site:RH2 | < 1.0 |
| 43 | Site:SD2 | < 1.0 |
| 44 | Site:T2_y-1_ | < 1.0 |
| 45 | Site:RH2_y-1_ | < 1.0 |
| 46 | Site:SD2_y-1_ | < 1.0 |
| 47 | Site:T2_y-2_ | < 1.0 |
| 48 | Site:RH2_y-2_ | < 1.0 |
| 49 | Site:SD2_y-2_ | < 1.0 |

Table S5. Model-averaged parameter estimates are shown for the generalized linear mixed effects model with binomial errors of the adult infection prevalence (AIP) response variable. Shown are the parameter types, the parameter names, the parameter estimates, and the 95% confidence limits (LL = lower limit and UL = upper limit). Estimate 1 is averaged over all the models in the set. Estimate 2 is averaged over the subset of models with a cumulative support of 95%. The 95% confidence limits are for estimate 2.

| **Type** | **Name** | **Estimate 1** | **Estimate 2** | **95% LL** | **95% UL** |
| --- | --- | --- | --- | --- | --- |
| **Intercept** | **Low site** | **-1.021** | **-1.021** | **-1.307** | **-0.736** |
| Contrast 1 | Medium site | 0.007 | 0.033 | -0.296 | 0.362 |
| Contrast 2 | High site | 0.052 | 0.255 | -0.093 | 0.604 |
| Contrast 3 | Top site | 0.058 | 0.280 | -0.147 | 0.708 |
| **Slope 1** | **Year** | **-0.001** | **-0.062** | **-0.111** | **-0.013** |
| **Slope 2** | **Beech_y-3_** | **-0.200** | **-0.202** | **-0.280** | **-0.123** |
| Slope 3 | RLB | 0.053 | 0.054 | -0.063 | 0.170 |
| Contrast 4 | T1 | 0.000 | 0.017 | -0.143 | 0.176 |
| Contrast 5 | RH1 | 0.000 | -0.130 | -0.291 | 0.031 |
| Contrast 6 | SD1 | 0.000 | 0.073 | -0.120 | 0.266 |
| Contrast 7 | PR | -0.002 | -0.299 | -0.510 | -0.089 |
| Contrast 8 | T1_y-1_ | 0.000 | -0.046 | -0.260 | 0.168 |
| Contrast 9 | RH1_y-1_ | 0.000 | -0.088 | -0.241 | 0.064 |
| Contrast 10 | SD1_y-1_ | 0.000 | 0.000 | -0.158 | 0.158 |
| Contrast 11 | PR_y-1_ | 0.000 | -0.129 | -0.303 | 0.045 |
| Contrast 12 | T1_y-2_ | 0.000 | 0.024 | -0.162 | 0.209 |
| Contrast 13 | RH1_y-2_ | 0.000 | 0.081 | -0.106 | 0.267 |
| Contrast 14 | SD1_y-2_ | 0.000 | -0.073 | -0.277 | 0.131 |
| Contrast 15 | PR_y-2_ | 0.001 | 0.165 | 0.023 | 0.307 |
| Contrast 16 | Medium site:Year | 0.000 | 0.027 | -0.088 | 0.142 |
| Contrast 17 | High site:Year | 0.000 | -0.007 | -0.122 | 0.109 |
| Contrast 18 | Top site:Year | 0.000 | 0.006 | -0.125 | 0.136 |
| Contrast 19 | Medium site:Beech_y-3_ | 0.000 | -0.009 | -0.244 | 0.225 |
| Contrast 20 | High site:Beech_y-3_ | 0.000 | 0.049 | -0.188 | 0.286 |
| Contrast 21 | Top site:Beech_y-3_ | 0.000 | -0.017 | -0.271 | 0.238 |
| Contrast 22 | Medium site:RLB | 0.000 | 0.075 | -0.270 | 0.420 |
| Contrast 23 | High site: RLB | 0.000 | 0.169 | -0.145 | 0.482 |
| Contrast 24 | Top site: RLB | 0.000 | 0.134 | -0.280 | 0.547 |
| Contrast 25 | Medium site:T1 | 0.000 | 0.240 | -0.601 | 1.081 |
| Contrast 26 | High site:T1 | 0.000 | 0.045 | -0.786 | 0.877 |
| Contrast 27 | Top site:T1 | 0.000 | -0.131 | -0.988 | 0.726 |
| Contrast 28 | Medium site:RH1 | 0.000 | 0.011 | -0.534 | 0.557 |
| Contrast 29 | High site:RH1 | 0.000 | 0.026 | -0.524 | 0.575 |
| Contrast 30 | Top site:RH1 | 0.000 | -0.209 | -0.789 | 0.370 |
| Contrast 31 | Medium site:SD1 | 0.000 | 0.037 | -0.555 | 0.628 |
| Contrast 32 | High site:SD1 | 0.000 | -0.025 | -0.650 | 0.600 |
| Contrast 33 | Top site:SD1 | 0.000 | 0.239 | -0.500 | 0.979 |
| Contrast 34 | Medium site:PR | 0.000 | 0.047 | -0.424 | 0.518 |
| Contrast 35 | High site:PR | 0.000 | 0.271 | -0.197 | 0.739 |
| Contrast 36 | Top site:PR | 0.000 | -0.032 | -0.526 | 0.462 |
| Contrast 37 | Medium site:T_y-1_ | 0.000 | 0.160 | -0.712 | 1.032 |
| Contrast 38 | High site:T_y-1_ | 0.000 | -0.045 | -0.907 | 0.816 |
| Contrast 39 | Top site:T_y-1_ | 0.000 | -0.074 | -0.974 | 0.825 |
| Contrast 40 | Medium site:RH1_y-1_ | 0.000 | 0.034 | -0.512 | 0.581 |
| Contrast 41 | High site:RH1_y-1_ | 0.000 | -0.041 | -0.597 | 0.515 |
| Contrast 42 | Top site:RH1_y-1_ | 0.000 | 0.098 | -0.514 | 0.709 |
| Contrast 43 | Medium site:SD1_y-1_ | 0.000 | 0.036 | -0.587 | 0.660 |
| Contrast 44 | High site:SD1_y-1_ | 0.000 | 0.148 | -0.517 | 0.813 |
| Contrast 45 | Top site:SD1_y-1_ | 0.000 | -0.096 | -0.930 | 0.739 |
| Contrast 46 | Medium site:PR_y-1_ | 0.000 | 0.102 | -0.348 | 0.551 |
| Contrast 47 | High site:PR_y-1_ | 0.000 | -0.031 | -0.484 | 0.423 |
| Contrast 48 | Top site:PR_y-1_ | 0.000 | -0.179 | -0.676 | 0.319 |
| Contrast 49 | Medium site:T1_y-2_ | 0.000 | 0.042 | -0.871 | 0.955 |
| Contrast 50 | High site:T1_y-2_ | 0.000 | -0.071 | -0.965 | 0.824 |
| Contrast 51 | Top site:T1_y-2_ | 0.000 | -0.056 | -0.987 | 0.874 |
| Contrast 52 | Medium site:RH1_y-2_ | 0.000 | 0.184 | -0.363 | 0.731 |
| Contrast 53 | High site:RH1_y-2_ | 0.000 | 0.027 | -0.515 | 0.570 |
| Contrast 54 | Top site:RH1_y-2_ | 0.000 | -0.054 | -0.633 | 0.524 |
| Contrast 55 | Medium site:SD1_y-2_ | 0.000 | -0.137 | -0.809 | 0.535 |
| Contrast 56 | High site:SD1_y-2_ | 0.000 | -0.064 | -0.752 | 0.623 |
| Contrast 57 | Top site:SD1_y-2_ | 0.000 | -0.035 | -0.810 | 0.740 |
| Contrast 58 | Medium site:PR_y-2_ | 0.000 | -0.028 | -0.453 | 0.396 |
| Contrast 59 | High site:PR_y-2_ | 0.000 | -0.276 | -0.706 | 0.154 |
| Contrast 60 | Top site:PR_y-2_ | 0.000 | -0.360 | -0.834 | 0.114 |
| Contrast 61 | T2 | 0.000 | 0.140 | -0.037 | 0.317 |
| Contrast 62 | RH2 | 0.000 | 0.113 | -0.062 | 0.289 |
| Contrast 63 | SD2 | 0.000 | -0.011 | -0.182 | 0.159 |
| Contrast 64 | T2_y-1_ | 0.000 | 0.058 | -0.092 | 0.208 |
| Contrast 65 | RH2_y-1_ | 0.000 | -0.062 | -0.222 | 0.098 |
| Contrast 66 | SD2_y-1_ | 0.000 | 0.051 | -0.102 | 0.204 |
| Contrast 67 | T2_y-2_ | 0.000 | 0.080 | -0.068 | 0.227 |
| **Contrast 68** | **RH2_y-2_** | **-0.298** | **-0.311** | **-0.457** | **-0.165** |
| Contrast 69 | SD2_y-2_ | 0.003 | 0.231 | 0.052 | 0.410 |
| Contrast 70 | Medium site:T2 | 0.000 | -0.111 | -0.821 | 0.599 |
| Contrast 71 | High site:T2 | 0.000 | -0.033 | -0.752 | 0.686 |
| Contrast 72 | Top site:T2 | 0.000 | -0.099 | -0.837 | 0.638 |
| Contrast 73 | Medium site:RH2 | 0.000 | -0.040 | -0.497 | 0.417 |
| Contrast 74 | High site:RH2 | 0.000 | -0.286 | -0.793 | 0.221 |
| Contrast 75 | Top site:RH2 | 0.000 | 0.125 | -0.444 | 0.694 |
| Contrast 76 | Medium site:SD2 | 0.000 | 0.007 | -0.517 | 0.531 |
| Contrast 77 | High site:SD2 | 0.000 | 0.350 | -0.286 | 0.987 |
| Contrast 78 | Top site:SD2 | 0.000 | -0.039 | -0.915 | 0.836 |
| Contrast 79 | Medium site:T2_y-1_ | 0.000 | -0.059 | -0.741 | 0.623 |
| Contrast 80 | High site:T2_y-1_ | 0.000 | 0.093 | -0.586 | 0.772 |
| Contrast 81 | Top site:T2_y-1_ | 0.000 | -0.053 | -0.763 | 0.658 |
| Contrast 82 | Medium site:RH2_y-1_ | 0.000 | -0.004 | -0.489 | 0.482 |
| Contrast 83 | High site:RH2_y-1_ | 0.000 | -0.112 | -0.631 | 0.408 |
| Contrast 84 | Top site:RH2_y-1_ | 0.000 | 0.300 | -0.284 | 0.885 |
| Contrast 85 | Medium site:SD2_y-1_ | 0.000 | -0.016 | -0.538 | 0.505 |
| Contrast 86 | High site:SD2_y-1_ | 0.000 | 0.187 | -0.392 | 0.766 |
| Contrast 87 | Top site:SD2_y-1_ | 0.000 | -0.208 | -0.962 | 0.547 |
| Contrast 88 | Medium site:T2_y-2_ | 0.000 | 0.101 | -0.611 | 0.813 |
| Contrast 89 | High site:T2_y-2_ | 0.000 | 0.025 | -0.684 | 0.734 |
| Contrast 90 | Top site:T2_y-2_ | 0.000 | -0.112 | -0.857 | 0.634 |
| Contrast 91 | Medium site:RH2_y-2_ | 0.000 | 0.064 | -0.394 | 0.522 |
| Contrast 92 | High site:RH2_y-2_ | 0.000 | 0.049 | -0.439 | 0.537 |
| Contrast 93 | Top site:RH2_y-2_ | 0.000 | 0.275 | -0.248 | 0.798 |
| Contrast 94 | Medium site:SD2_y-2_ | 0.000 | 0.116 | -0.390 | 0.622 |
| Contrast 95 | High site:SD2_y-2_ | 0.000 | 0.096 | -0.466 | 0.658 |
| Contrast 96 | Top site:SD2_y-2_ | 0.000 | -0.178 | -0.855 | 0.500 |

Table S6. Model selection results are shown for the linear models with normal errors of the log10-trasnformed density of infected adults (DIA) response variable. The explanatory variables were site, year, beech masting index 3 years prior, RLB time lag, and the climate variables obtained from the weather stations and collected from the field. The models are ranked according to their Akaike Information Criterion (AIC). Shown for each model are the model rank (Rank), model structure (see below for explanation of explanatory variables), model degrees of freedom (Df), log-likelihood (logLik), Akaike information criterion (AIC), difference in the AIC value from the top model (ΔAIC), model weight (Weight1), and cumulative weight (Weight2), and adjusted r-squared (r^2^).

| **Rank** | **Model structure** | **Df** | **logLik** | **AIC** | **ΔAIC** | **Weight1** | **Weight2** | **r^2^** |
| --- | --- | --- | --- | --- | --- | --- | --- | --- |
| 1 | DIA ~ S+Y+RLB+RH2_y-2_+S:Y | 11 | 5.1 | 18.5 | 0.0 | 35.0 | 35.0 | 62.7 |
| 2 | DIA ~ S+Y+B3+RH2_y-2_+S:Y | 11 | 4.4 | 19.8 | 1.3 | 18.0 | 53.0 | 61.8 |
| 3 | DIA ~ S+RLB+RH2_y-2_ | 7 | -2.3 | 21.2 | 2.7 | 9.0 | 62.0 | 54.7 |
| 4 | DIA ~ S+Y+B3+T2+S:Y | 11 | 2.6 | 23.4 | 5.0 | 3.0 | 65.0 | 58.9 |
| 5 | DIA ~ S+Y+RLB+RH2_y-2_ | 8 | -2.1 | 23.5 | 5.1 | 3.0 | 68.0 | 54.1 |
| 6 | DIA ~ S+Y+RLB+RH2_y-2_ | 8 | -2.1 | 23.5 | 5.1 | 3.0 | 71.0 | 54.1 |
| 7 | DIA ~ S+Y+RLB+T2+S:Y | 11 | 2.5 | 23.6 | 5.1 | 3.0 | 74.0 | 58.8 |
| 8 | DIA ~ S+B3+RLB+RH2_y-2_ | 8 | -2.2 | 23.7 | 5.3 | 2.0 | 76.0 | 53.9 |
| 9 | DIA ~ S+Y+B3+RH2_y-2_ | 8 | -2.2 | 23.8 | 5.4 | 2.0 | 78.0 | 53.9 |
| 10 | DIA ~ S+Y+B3+SD2_y-2_+S:Y | 11 | 1.9 | 24.8 | 6.3 | 1.0 | 79.0 | 57.9 |
| 11 | DIA ~ S+Y+RLB+SD2_y-2_+S:Y | 11 | 1.5 | 25.6 | 7.1 | 1.0 | 80.0 | 57.2 |
| 12 | DIA ~ S+Y+B3+RH1+S:Y | 11 | 1.5 | 25.7 | 7.2 | 1.0 | 81.0 | 57.1 |
| 13 | DIA ~ S+RH2_y-2_ | 9 | -1.8 | 25.8 | 7.4 | 1.0 | 82.0 | 53.7 |
| 14 | DIA ~ S+Y+S:Y | 9 | -1.9 | 26.1 | 7.7 | 1.0 | 83.0 | 53.4 |
| 15 | DIA ~ S+Y+B3+SD2+S:Y | 11 | 1.1 | 26.3 | 7.9 | 1.0 | 84.0 | 56.6 |
| 16 | DIA ~ S+RLB+RH2_y-2_ | 10 | -0.6 | 26.5 | 8.0 | 1.0 | 85.0 | 54.8 |
| 17 | DIA ~ S+Y+B3+RH1 | 8 | -3.7 | 26.8 | 8.3 | 1.0 | 86.0 | 51.2 |
| 18 | DIA ~ S+Y+B3+T2 | 8 | -3.7 | 26.8 | 8.3 | 1.0 | 87.0 | 51.2 |
| 19 | DIA ~ S+Y+B3+S:Y | 10 | -0.8 | 27.1 | 8.6 | 0.0 | 100.0 | 54.3 |
| 20 | DIA ~ S+RLB+SD2_y-2_ | 10 | -0.8 | 27.1 | 8.6 | 0.0 | 100.0 | 54.3 |
| 21 | DIA ~ S+Y+RLB+T2 | 7 | -5.3 | 27.2 | 8.8 | 0.0 | 100.0 | 49.2 |
| 22 | DIA ~ S+Y+RLB+SD1_y-2_+S:Y | 8 | -3.9 | 27.2 | 8.8 | 0.0 | 100.0 | 50.8 |
| 23 | DIA ~ S+B3+RH2_y-2_+S:RH2_y-2_ | 11 | 0.6 | 27.3 | 8.9 | 0.0 | 100.0 | 55.8 |
| 24 | DIA ~ S+Y+B3+S:Y+RH2_y-1_ | 10 | -1.1 | 27.5 | 9.1 | 0.0 | 100.0 | 53.8 |
| 25 | DIA ~ S+Y+RLB+SD2+S:Y | 11 | 0.5 | 27.5 | 9.1 | 0.0 | 100.0 | 55.6 |
| 26 | DIA ~ S+Y+B3+SD1_y-2_+S:Y | 11 | 0.5 | 27.6 | 9.1 | 0.0 | 100.0 | 55.6 |
| 27 | DIA ~ S+Y+B3+SD1+S:Y | 11 | 0.5 | 27.6 | 9.1 | 0.0 | 100.0 | 55.5 |
| 28 | DIA ~ S+Y+RLB+RH1_y-2_+S:Y | 11 | 0.5 | 27.6 | 9.2 | 0.0 | 100.0 | 55.5 |
| 29 | DIA ~ S+Y+B3+SD2 | 11 | 0.4 | 27.8 | 9.4 | 0.0 | 100.0 | 55.3 |
| 30 | DIA ~ S+Y+B3+S:Y+SD2_y-1_ | 8 | -4.3 | 28.0 | 9.5 | 0.0 | 100.0 | 50.0 |

Table S7. The support for each explanatory variable is shown from the AIC-based model selection table of the density of infected adults. This support is calculated as the sum of the Akaike weights for all the models in the set that include that explanatory variable.

| **Rank** | **Explanatory variable of interest** | **Support (%)** |
| --- | --- | --- |
| 1 | Site | 99.9 |
| 2 | Year | 85.4 |
| 3 | RH2_y-2_ | 74.0 |
| 4 | Site:Year | 69.6 |
| 5 | RLB | 63.8 |
| 6 | Beech_y-3_ | 37.3 |
| 7 | T2 | 7.0 |
| 8 | SD2_y-2_ | 3.7 |
| 9 | Site:RH2_y-2_ | 1.9 |
| 10 | RH1 | 1.9 |
| 11 | SD2 | 1.7 |
| 12 | SD1_y-2_ | 1.3 |
| 13 | PR_y-1_ | < 1.0 |
| 14 | SD1 | < 1.0 |
| 15 | T1_y-1_ | < 1.0 |
| 16 | PR | < 1.0 |
| 17 | T1 | < 1.0 |
| 18 | RH1_y-1_ | < 1.0 |
| 19 | SD1_y-1_ | < 1.0 |
| 20 | T1_y-2_ | < 1.0 |
| 21 | RH1_y-2_ | < 1.0 |
| 22 | PR_y-2_ | < 1.0 |
| 23 | RH2 | < 1.0 |
| 24 | T2_y-1_ | < 1.0 |
| 25 | RH2_y-1_ | < 1.0 |
| 26 | SD2_y-1_ | < 1.0 |
| 27 | T2_y-2_ | < 1.0 |
| 28 | Site:Beech_y-3_ | < 1.0 |
| 29 | Site:RLB | < 1.0 |
| 30 | Site:T1 | < 1.0 |
| 31 | Site:RH1 | < 1.0 |
| 32 | Site:SD1 | < 1.0 |
| 33 | Site:PR | < 1.0 |
| 34 | Site:T1_y-1_ | < 1.0 |
| 35 | Site:RH1_y-1_ | < 1.0 |
| 36 | Site:SD1_y-1_ | < 1.0 |
| 37 | Site:PR_y-1_ | < 1.0 |
| 38 | Site:T1_y-2_ | < 1.0 |
| 39 | Site:RH1_y-2_ | < 1.0 |
| 40 | Site:SD1_y-2_ | < 1.0 |
| 41 | Site:PR_y-2_ | < 1.0 |
| 42 | Site:T2 | < 1.0 |
| 43 | Site:RH2 | < 1.0 |
| 44 | Site:SD2 | < 1.0 |
| 45 | Site:T2_y-1_ | < 1.0 |
| 46 | Site:RH2_y-1_ | < 1.0 |
| 47 | Site:SD2_y-1_ | < 1.0 |
| 48 | Site:T2_y-2_ | < 1.0 |
| 49 | Site:SD2_y-2_ | < 1.0 |

Table S8. Model-averaged parameter estimates are shown for the linear model with normal errors of the density of infected adults (DIA) response variable. Shown are the parameter types, the parameter names, the parameter estimates, and the 95% confidence limits (LL = lower limit and UL = upper limit). Estimate 1 is averaged over all the models in the set. Estimate 2 is averaged over the subset of models with a cumulative support of 95%. The 95% confidence limits are for estimate 2.

| **Type** | **Name** | **Estimate 1** | **Estimate 2** | **95% LL** | **95% UL** |
| --- | --- | --- | --- | --- | --- |
| **Intercept** | **Low site** | **2.474** | **2.474** | **1.964** | **2.983** |
| Contrast 1 | Medium site | 0.381 | 0.381 | -0.078 | 0.841 |
| Contrast 2 | High site | 0.544 | 0.544 | -0.122 | 1.210 |
| Contrast 3 | Top site | 0.243 | 0.243 | -0.594 | 1.080 |
| Slope 1 | Year | 0.015 | 0.017 | -0.038 | 0.072 |
| Slope 2 | Beech_y-3_ | 0.008 | 0.021 | -0.025 | 0.068 |
| Slope 3 | RLB | 0.030 | 0.047 | -0.054 | 0.148 |
| Contrast 4 | T1 | 0.000 | -0.032 | -0.274 | 0.209 |
| Contrast 5 | RH1 | -0.002 | -0.092 | -0.196 | 0.012 |
| Contrast 6 | SD1 | 0.001 | 0.094 | -0.054 | 0.243 |
| Contrast 7 | PR | 0.000 | -0.034 | -0.139 | 0.070 |
| Contrast 8 | T1_y-1_ | 0.000 | -0.005 | -0.203 | 0.193 |
| Contrast 9 | RH1_y-1_ | 0.000 | -0.017 | -0.122 | 0.087 |
| Contrast 10 | SD1_y-1_ | 0.000 | 0.037 | -0.100 | 0.174 |
| Contrast 11 | PR_y-1_ | 0.000 | -0.026 | -0.117 | 0.066 |
| Contrast 12 | T1_y-2_ | 0.000 | 0.077 | -0.114 | 0.267 |
| Contrast 13 | RH1_y-2_ | -0.001 | -0.073 | -0.196 | 0.050 |
| Contrast 14 | SD1_y-2_ | 0.001 | 0.109 | -0.031 | 0.248 |
| Contrast 15 | PR_y-2_ | 0.000 | -0.030 | -0.119 | 0.060 |
| Contrast 16 | Medium site:Year | -0.008 | -0.011 | -0.064 | 0.042 |
| **Contrast 17** | **High site:Year** | **-0.040** | **-0.058** | **-0.111** | **-0.004** |
| **Contrast 18** | **Top site:Year** | **-0.055** | **-0.079** | **-0.133** | **-0.026** |
| Contrast 19 | Medium site:Beech_y-3_ | 0.000 | 0.005 | -0.129 | 0.139 |
| Contrast 20 | High site:Beech_y-3_ | 0.000 | -0.055 | -0.191 | 0.080 |
| Contrast 21 | Top site:Beech_y-3_ | 0.000 | -0.052 | -0.189 | 0.085 |
| Contrast 22 | Medium site:RLB | 0.000 | -0.102 | -0.322 | 0.118 |
| Contrast 23 | High site: RLB | 0.000 | -0.112 | -0.331 | 0.106 |
| Contrast 24 | Top site: RLB | 0.000 | 0.142 | -0.078 | 0.362 |
| Contrast 25 | Medium site:T1 | 0.000 | 0.125 | -0.331 | 0.581 |
| Contrast 26 | High site:T1 | 0.000 | -0.066 | -0.514 | 0.381 |
| Contrast 27 | Top site:T1 | 0.000 | -0.428 | -0.867 | 0.011 |
| Contrast 28 | Medium site:RH1 | 0.000 | 0.052 | -0.266 | 0.371 |
| Contrast 29 | High site:RH1 | 0.000 | -0.043 | -0.359 | 0.274 |
| Contrast 30 | Top site:RH1 | 0.000 | 0.080 | -0.228 | 0.387 |
| Contrast 31 | Medium site:SD1 | 0.000 | -0.018 | -0.368 | 0.332 |
| Contrast 32 | High site:SD1 | 0.000 | 0.003 | -0.361 | 0.368 |
| Contrast 33 | Top site:SD1 | 0.000 | -0.200 | -0.580 | 0.180 |
| Contrast 34 | Medium site:PR | 0.000 | 0.074 | -0.191 | 0.338 |
| Contrast 35 | High site:PR | 0.000 | 0.143 | -0.120 | 0.406 |
| Contrast 36 | Top site:PR | 0.000 | 0.157 | -0.103 | 0.417 |
| Contrast 37 | Medium site:T_y-1_ | 0.000 | -0.045 | -0.552 | 0.463 |
| Contrast 38 | High site:T_y-1_ | 0.000 | -0.186 | -0.682 | 0.310 |
| Contrast 39 | Top site:T_y-1_ | 0.000 | -0.307 | -0.791 | 0.178 |
| Contrast 40 | Medium site:RH1_y-1_ | 0.000 | 0.036 | -0.293 | 0.366 |
| Contrast 41 | High site:RH1_y-1_ | 0.000 | -0.003 | -0.331 | 0.324 |
| Contrast 42 | Top site:RH1_y-1_ | 0.000 | -0.002 | -0.321 | 0.316 |
| Contrast 43 | Medium site:SD1_y-1_ | 0.000 | -0.031 | -0.403 | 0.341 |
| Contrast 44 | High site:SD1_y-1_ | 0.000 | -0.088 | -0.474 | 0.297 |
| Contrast 45 | Top site:SD1_y-1_ | 0.000 | -0.169 | -0.568 | 0.229 |
| Contrast 46 | Medium site:PR_y-1_ | 0.000 | -0.011 | -0.280 | 0.258 |
| Contrast 47 | High site:PR_y-1_ | 0.000 | -0.063 | -0.332 | 0.205 |
| Contrast 48 | Top site:PR_y-1_ | 0.000 | -0.040 | -0.306 | 0.225 |
| Contrast 49 | Medium site:T1_y-2_ | 0.000 | -0.203 | -0.708 | 0.302 |
| Contrast 50 | High site:T1_y-2_ | 0.000 | -0.317 | -0.808 | 0.175 |
| Contrast 51 | Top site:T1_y-2_ | 0.000 | -0.440 | -0.919 | 0.039 |
| Contrast 52 | Medium site:RH1_y-2_ | 0.000 | 0.106 | -0.243 | 0.455 |
| Contrast 53 | High site:RH1_y-2_ | 0.000 | 0.112 | -0.229 | 0.454 |
| Contrast 54 | Top site:RH1_y-2_ | 0.000 | 0.033 | -0.293 | 0.360 |
| Contrast 55 | Medium site:SD1_y-2_ | 0.000 | -0.138 | -0.535 | 0.260 |
| Contrast 56 | High site:SD1_y-2_ | 0.000 | -0.190 | -0.597 | 0.216 |
| Contrast 57 | Top site:SD1_y-2_ | 0.000 | -0.255 | -0.665 | 0.156 |
| Contrast 58 | Medium site:PR_y-2_ | 0.000 | 0.055 | -0.211 | 0.321 |
| Contrast 59 | High site:PR_y-2_ | 0.000 | -0.012 | -0.280 | 0.255 |
| Contrast 60 | Top site:PR_y-2_ | 0.000 | -0.054 | -0.322 | 0.214 |
| Contrast 61 | T2 | 0.011 | 0.152 | 0.020 | 0.284 |
| Contrast 62 | RH2 | 0.000 | -0.052 | -0.158 | 0.054 |
| Contrast 63 | SD2 | 0.002 | 0.101 | -0.021 | 0.224 |
| Contrast 64 | T2_y-1_ | 0.000 | 0.063 | -0.059 | 0.184 |
| Contrast 65 | RH2_y-1_ | -0.001 | -0.059 | -0.172 | 0.054 |
| Contrast 66 | SD2_y-1_ | 0.000 | 0.064 | -0.063 | 0.190 |
| Contrast 67 | T2_y-2_ | 0.000 | 0.056 | -0.080 | 0.193 |
| **Contrast 68** | **RH2_y-2_** | **-0.128** | **-0.173** | **-0.289** | **-0.057** |
| Contrast 69 | SD2_y-2_ | 0.006 | 0.147 | 0.002 | 0.292 |
| Contrast 70 | Medium site:T2 | 0.000 | 0.006 | -0.366 | 0.377 |
| Contrast 71 | High site:T2 | 0.000 | -0.038 | -0.414 | 0.338 |
| Contrast 72 | Top site:T2 | 0.000 | -0.379 | -0.754 | -0.005 |
| Contrast 73 | Medium site:RH2 | 0.000 | -0.087 | -0.338 | 0.163 |
| Contrast 74 | High site:RH2 | 0.000 | -0.140 | -0.422 | 0.141 |
| Contrast 75 | Top site:RH2 | 0.000 | 0.205 | -0.079 | 0.489 |
| Contrast 76 | Medium site:SD2 | 0.000 | 0.096 | -0.191 | 0.383 |
| Contrast 77 | High site:SD2 | 0.000 | 0.232 | -0.120 | 0.584 |
| Contrast 78 | Top site:SD2 | 0.000 | -0.238 | -0.657 | 0.182 |
| Contrast 79 | Medium site:T2_y-1_ | 0.000 | 0.117 | -0.274 | 0.508 |
| Contrast 80 | High site:T2_y-1_ | 0.000 | 0.177 | -0.211 | 0.564 |
| Contrast 81 | Top site:T2_y-1_ | 0.000 | 0.093 | -0.297 | 0.483 |
| Contrast 82 | Medium site:RH2_y-1_ | 0.000 | -0.115 | -0.362 | 0.132 |
| Contrast 83 | High site:RH2_y-1_ | 0.000 | -0.158 | -0.424 | 0.108 |
| Contrast 84 | Top site:RH2_y-1_ | 0.000 | 0.180 | -0.094 | 0.454 |
| Contrast 85 | Medium site:SD2_y-1_ | 0.000 | 0.157 | -0.125 | 0.439 |
| Contrast 86 | High site:SD2_y-1_ | 0.000 | 0.263 | -0.054 | 0.581 |
| Contrast 87 | Top site:SD2_y-1_ | 0.000 | -0.030 | -0.406 | 0.347 |
| Contrast 88 | Medium site:T2_y-2_ | 0.000 | -0.100 | -0.512 | 0.311 |
| Contrast 89 | High site:T2_y-2_ | 0.000 | -0.064 | -0.471 | 0.343 |
| Contrast 90 | Top site:T2_y-2_ | 0.000 | -0.032 | -0.441 | 0.377 |
| Contrast 91 | Medium site:RH2_y-2_ | 0.000 | -0.011 | -0.245 | 0.223 |
| Contrast 92 | High site:RH2_y-2_ | -0.002 | -0.117 | -0.366 | 0.132 |
| Contrast 93 | Top site:RH2_y-2_ | 0.002 | 0.103 | -0.152 | 0.358 |
| Contrast 94 | Medium site:SD2_y-2_ | 0.000 | 0.014 | -0.263 | 0.291 |
| Contrast 95 | High site:SD2_y-2_ | 0.000 | 0.167 | -0.139 | 0.474 |
| Contrast 96 | Top site:SD2_y-2_ | 0.000 | 0.034 | -0.317 | 0.385 |


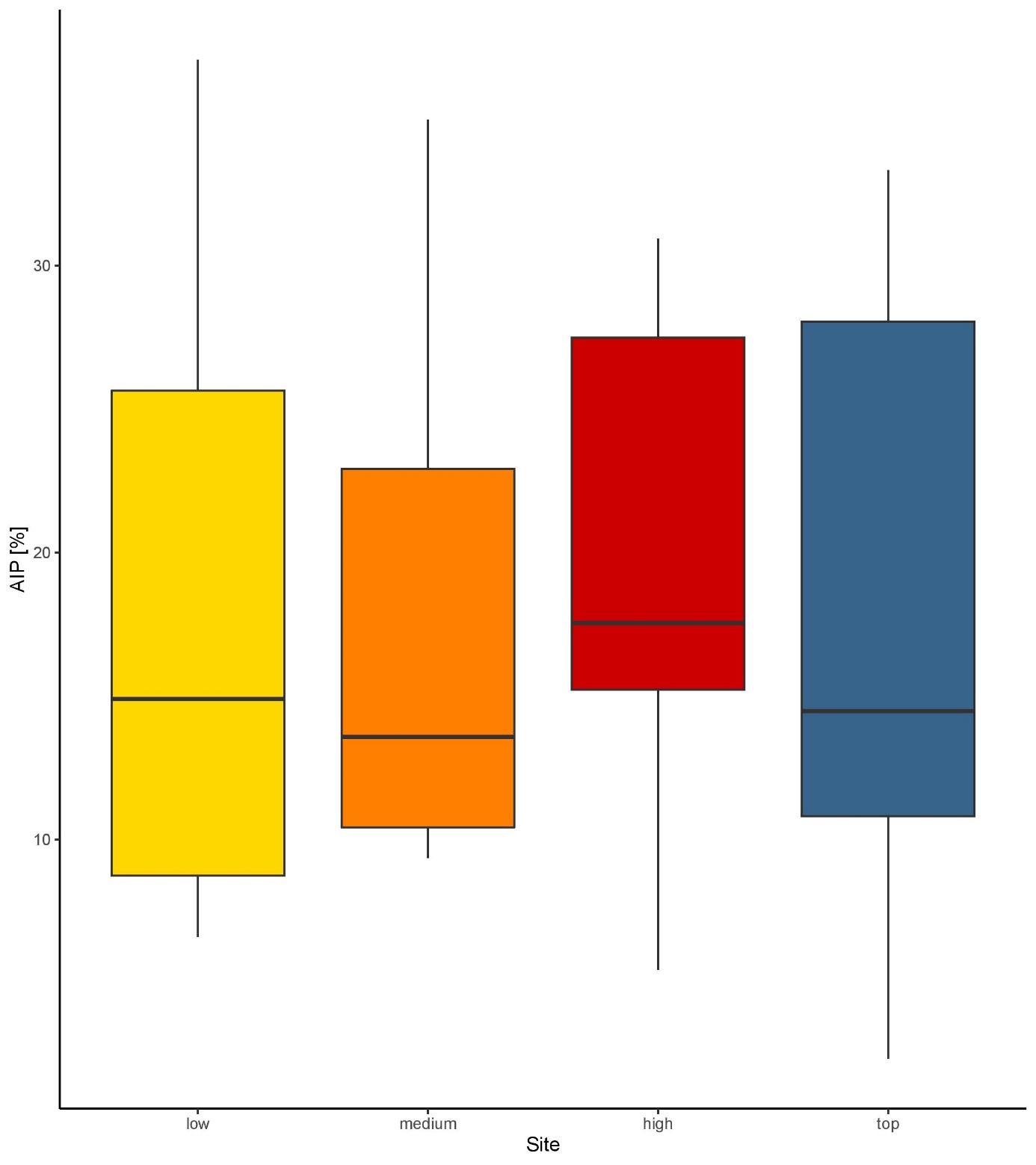
Figure S2. Effect of elevation on the adult infection prevalence (AIP), which is the percentage of *I. ricinus* nymphs infected with *B. burgdorferi* sl. For each of the four elevation sites, the mean NIP for the 15 years of the study is shown (2004–2018). Compared to the low elevation site, the AIP was 2.6% and 23.3% lower at the medium and top elevation sites, but 21.1% higher at the high elevation site. The boxplots show the median (black line), 25th and 75th percentiles (edges of the box), minimum and maximum values (whiskers), and outliers (solid circles).


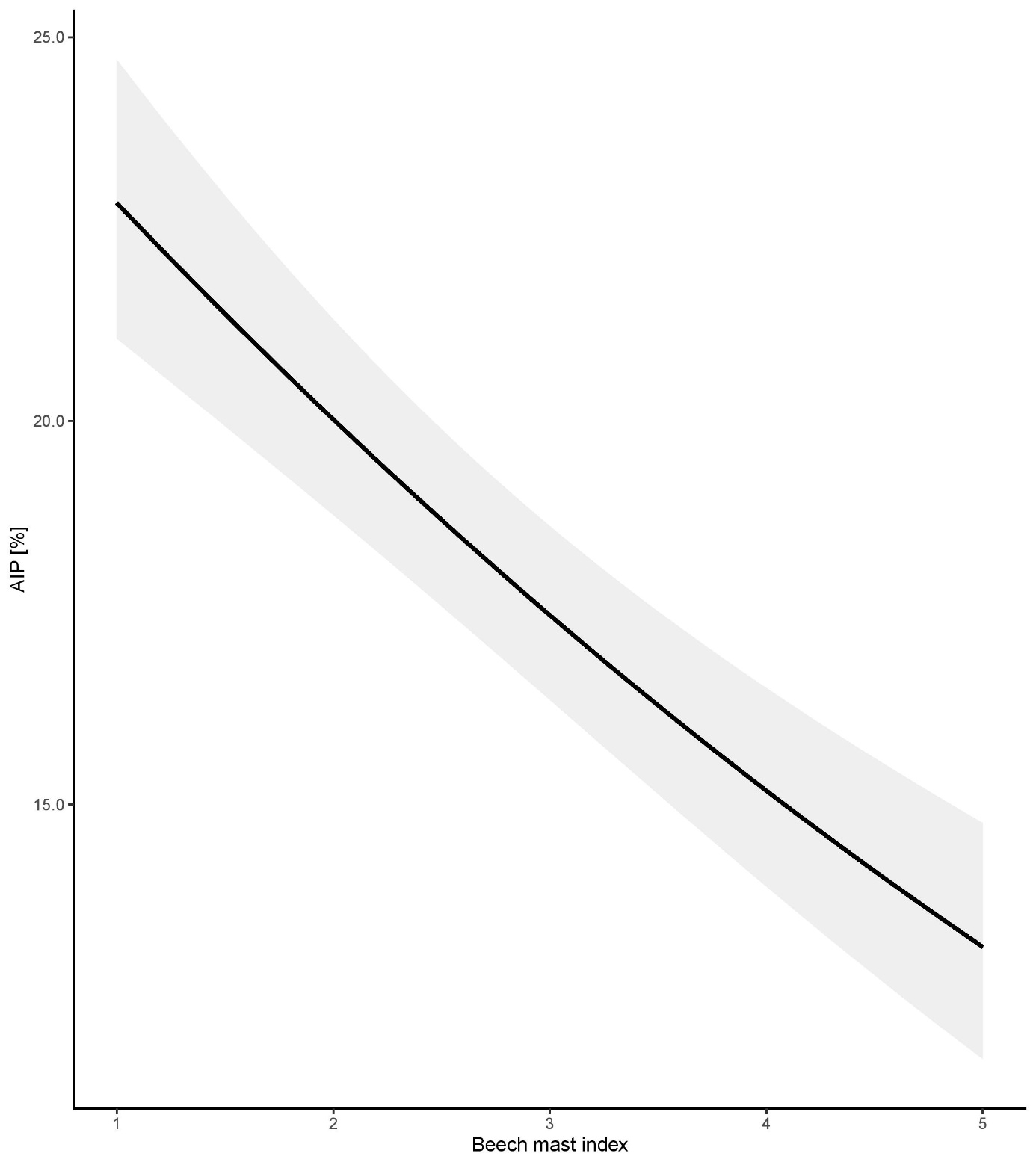
Figure S3. Effect of beech mast score 3 years prior on the adult infection prevalence (AIP), which is the the percentage of *I. ricinus* adults infected with *B. burgdorferi* sl. The beech tree mast score 3 years prior ranges from 1 (poor mast) to 5 (full mast) and was obtained from the MASTREE database.The parameter estimates used to calculate the effect sizes were taken from the model-averaged parameter estimates in Table S5 Increasing the beech mast score from 1 (poor mast) to 5 (full mast) decreased the AIP by 34.9% to 48.9% at the four elevation sites.


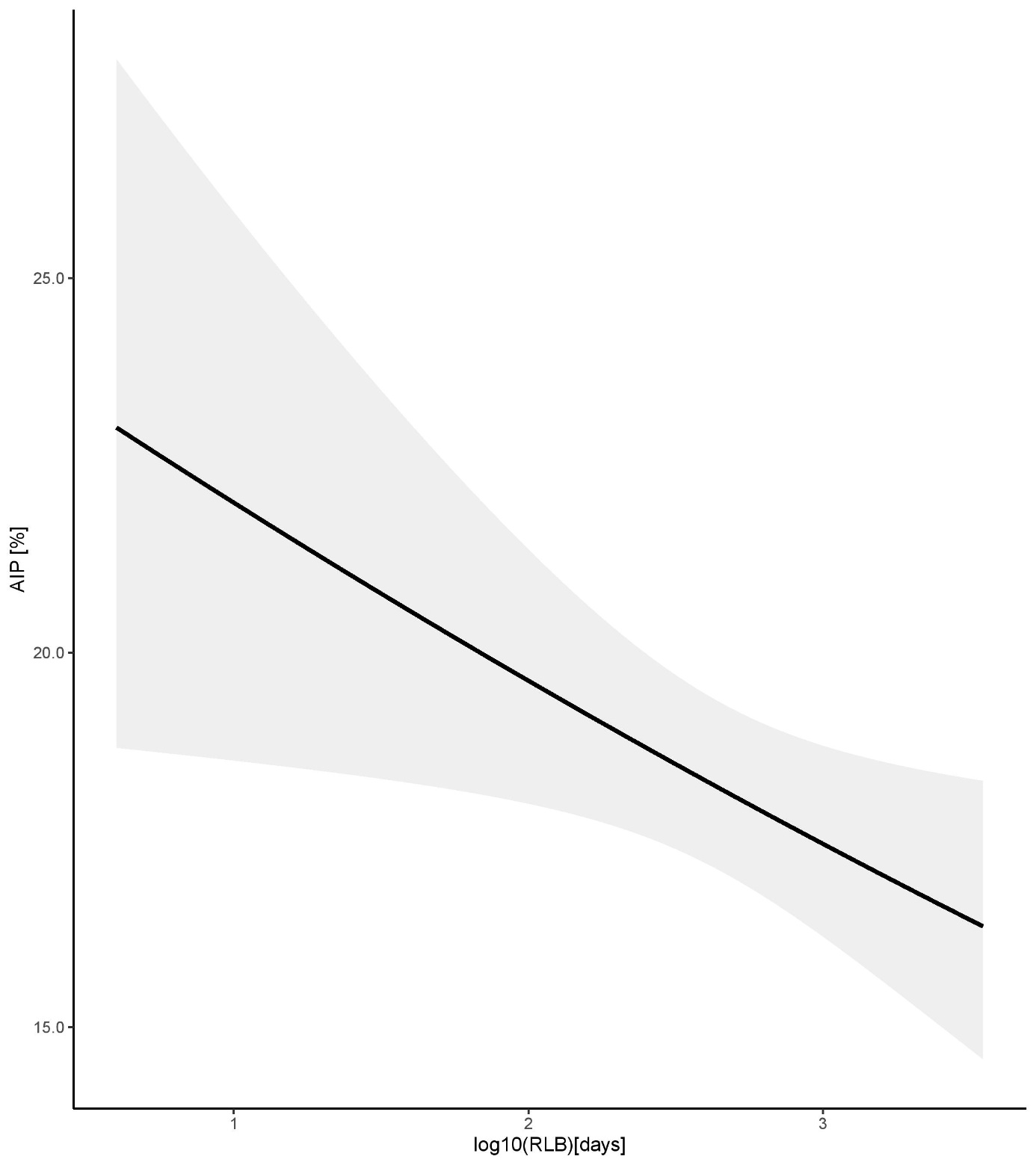
Figure S4. Effect of the reverse line blot (RLB) time lag on the adult infection prevalence (AIP), which is the percentage of *I. ricinus* adults infected with *B. burgdorferi* sl. The RLB time lag is the time interval between the date of collecting the ticks in the field and the date of testing the tick infection status using the RLB.. The parameter estimates used to calculate the effect sizes were taken from the model-averaged parameter estimates in Table S5. Increasing the RLB time lag by one standard deviation (e.g. 925 days) increased the AIP by 4.2% to 28.1% at the four elevation sites.


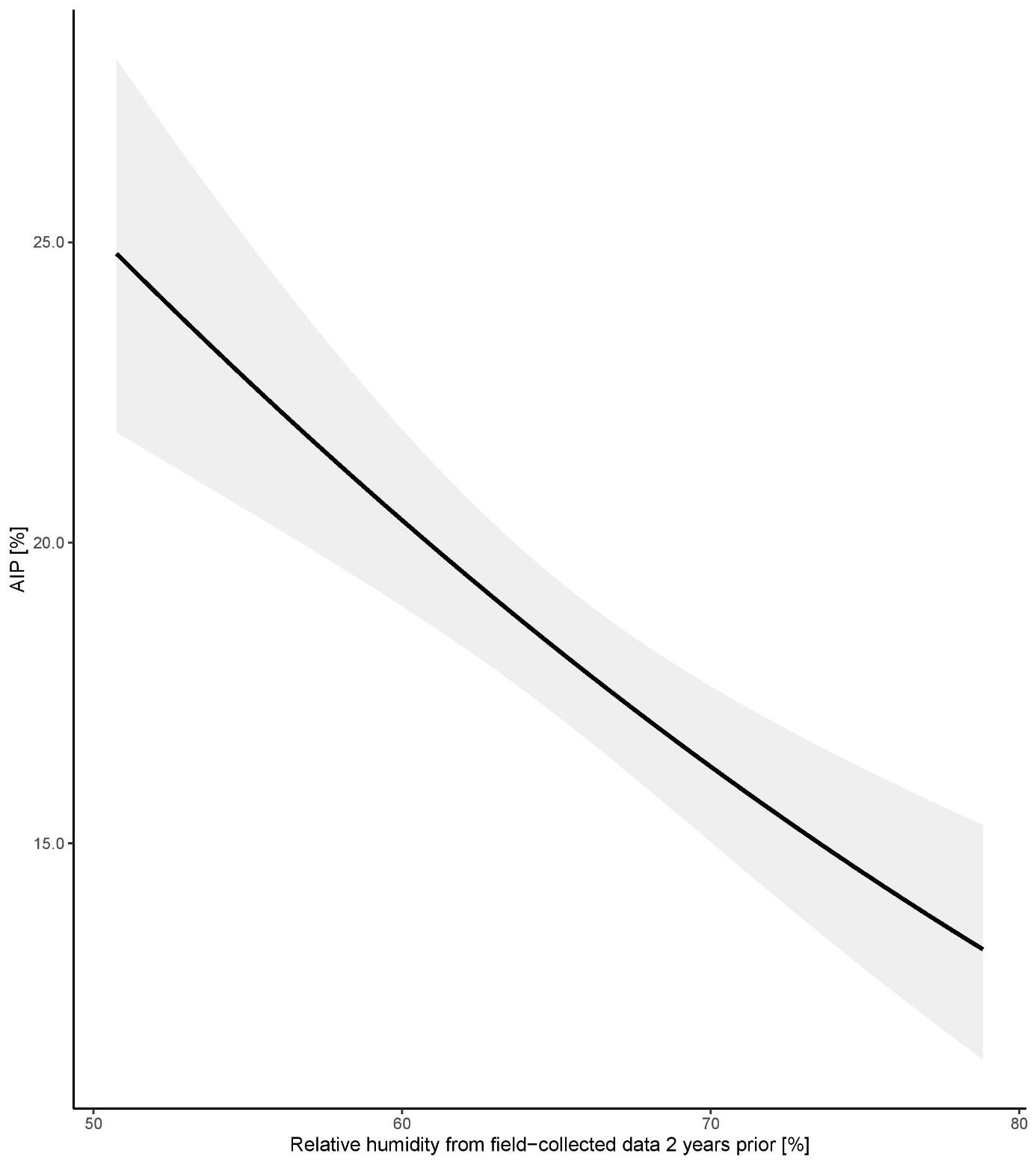
Figure S5. Effect of the mean annual field-collected relative humidity 2 years prior on the adult infection prevalence (AIP), which is the percentage of *I. ricinus* adults infected with *B. burgdorferi* sl. The mean annual relative humidity has units of % and was measured at 50 cm above ground on the day of sampling at the field site. The parameter estimates used to calculate the effect sizes were taken from the model-averaged parameter estimates in Table S5. Increasing the relative humidity 2 years prior by one standard deviation (e.g. 7.1% of relative humidity) decreased AIP by 2.4% to 22.0% at the four elevation sites.
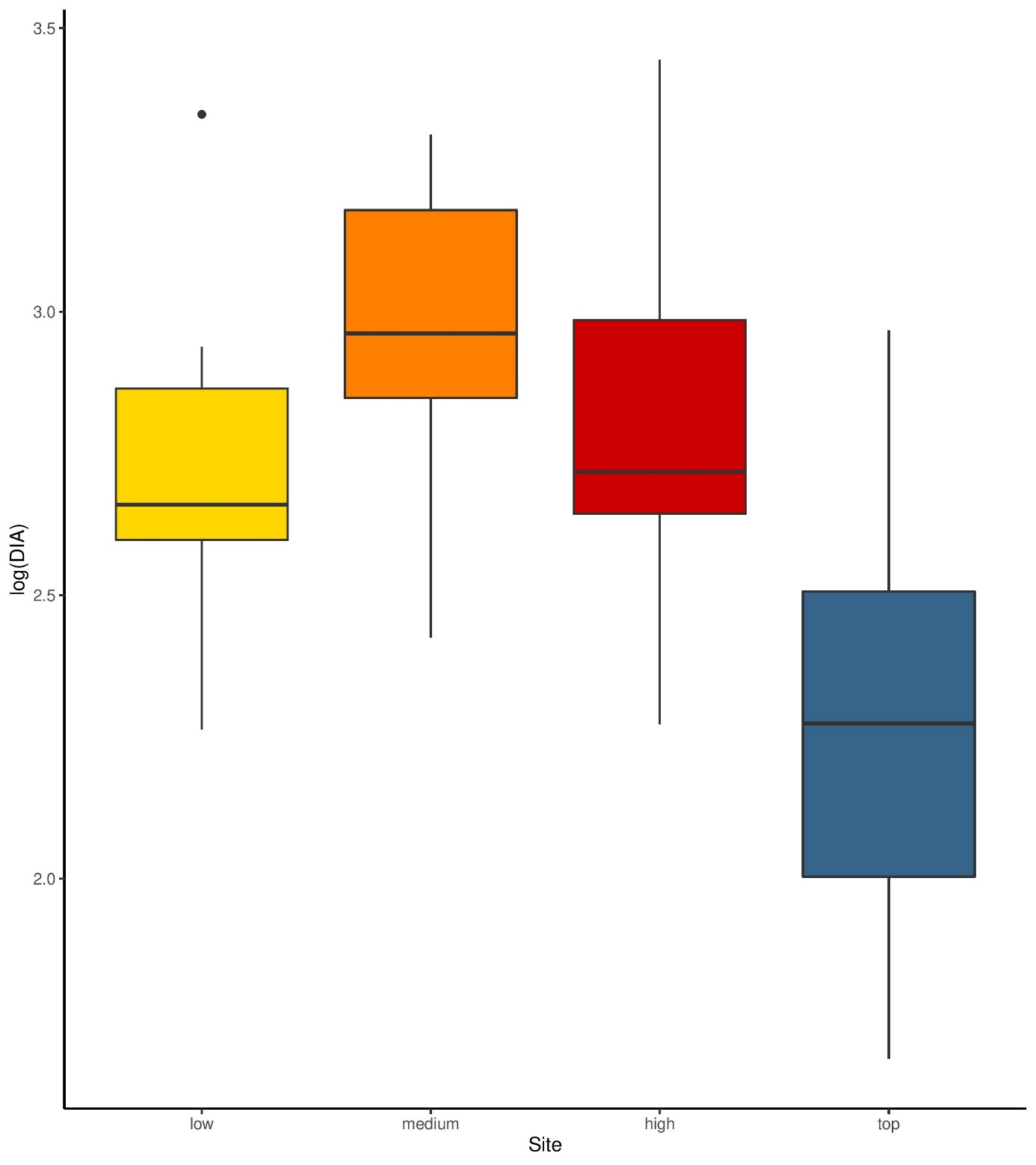
Figure S6. Effect of elevation on the density of infected adult (DIA). The DIA is an estimate of the number of infected questing *I. ricinus* nymphs per 100 m^2^ sampled by the dragging method each year. For each of the four elevation sites, the mean DIA for the 15 years of the study is shown (2004–2018). Compared to the low elevation site, the mean DIA at the medium, high, and top elevation sites were 134.5%, 206.6%, and 45.8% lower, respectively. The boxplots show the median (black line), 25th and 75th percentiles (edges of the box), minimum and maximum values (whiskers), and outliers (solid circles).


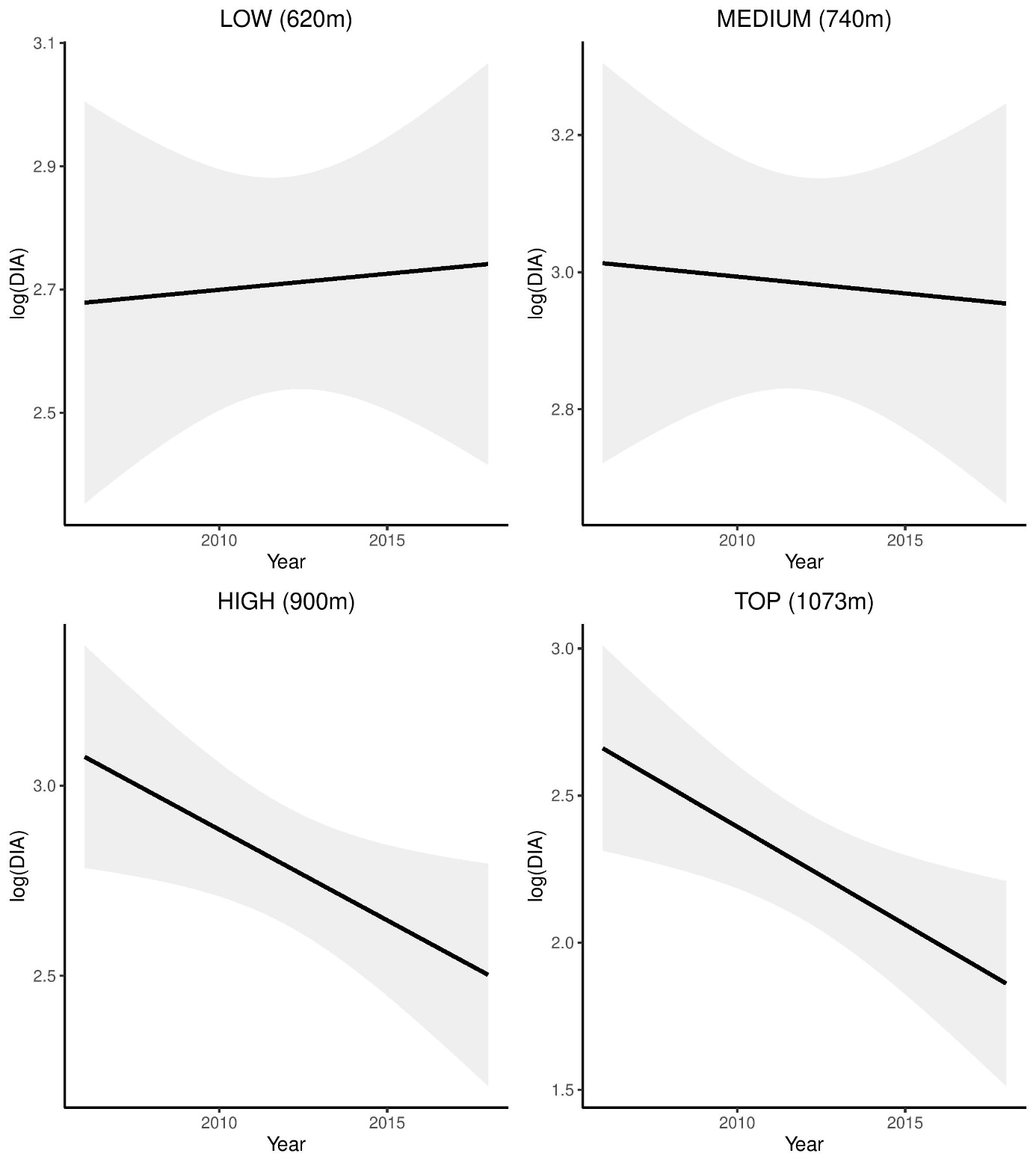
Figure S7. Effect of year on the density of infected adults (DIA). The DIA is an estimate of the number of infected questing *I. ricinus* adults per 100 m^2^ sampled by the dragging method each year. The parameter estimates used to calculate the effect sizes were taken from the model-averaged parameter estimates in Table S8. The DIA increased by 60.1% at the low elevation site, but decreased by 17.1%, 67.5%, and 82.1% at the medium, high, and top elevation sites, respectively, over the 13-year study period.


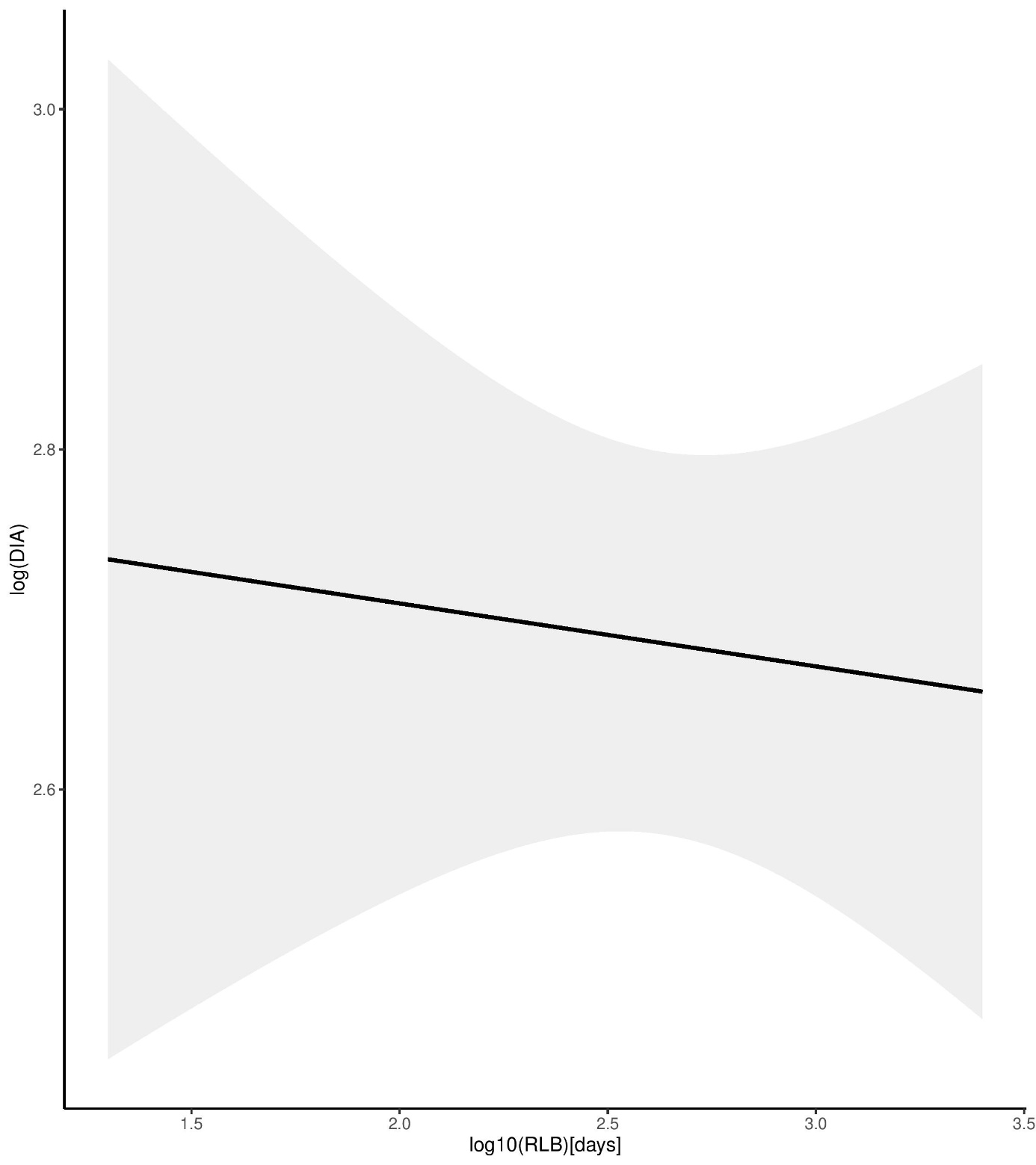
Figure S8. Effect of the reverse line blot (RLB) time lag on the density of infected adults (DIA), which is an estimate of the number of infected questing *I. ricinus* adults per 100 m^2^ sampled by the dragging method each year. The RLB time lag is the time interval between the date of collecting the ticks in the field and the date of testing the tick infection status using the RLB. The parameter estimates used to calculate the effect sizes were taken from the model-averaged parameter estimates in Table S8. Increasing the RLB time lag by one standard deviation (e.g. 925 days) increased the DIA by 11.4% at each of the four elevation sites.


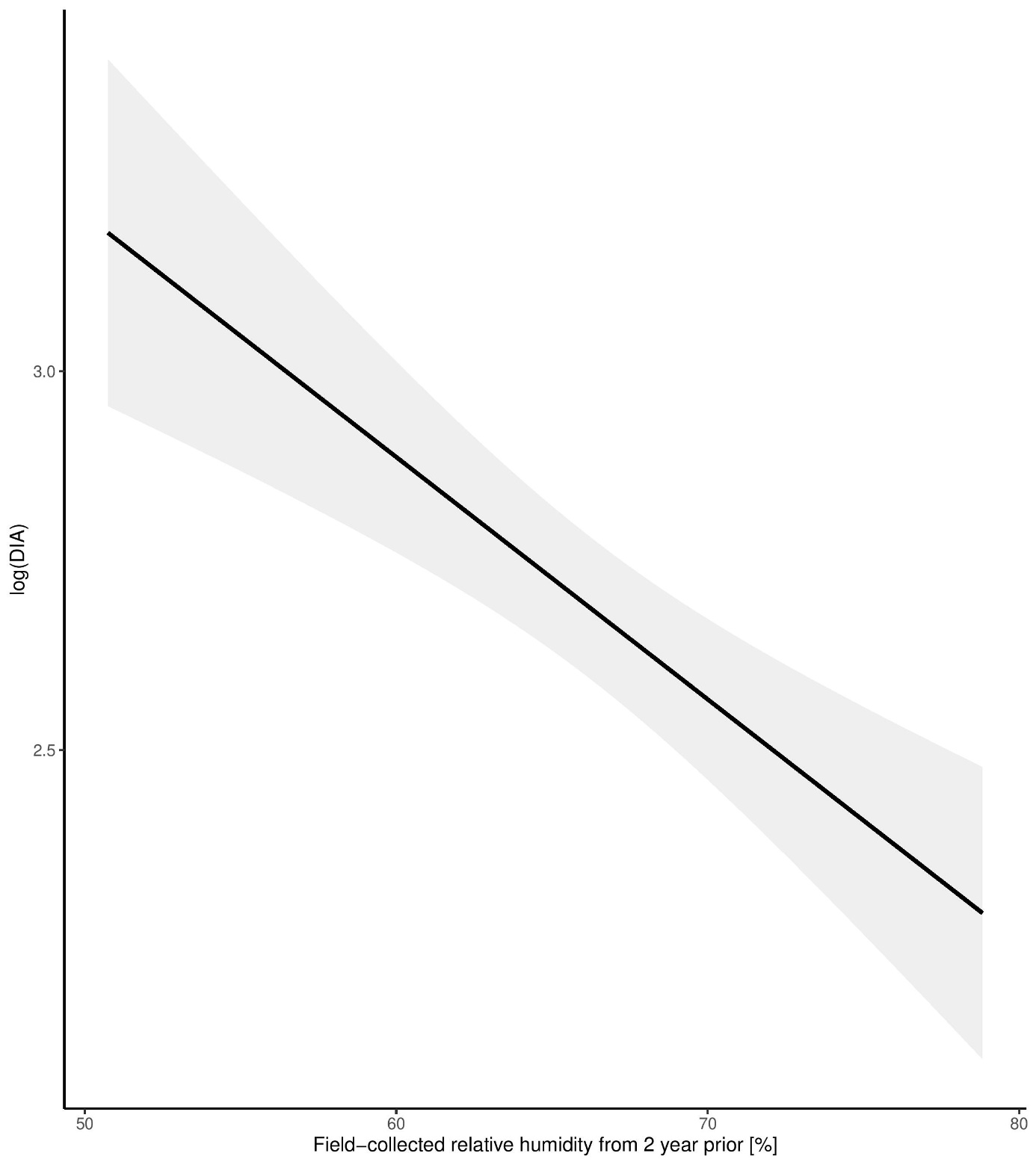
Figure S9. Effect of the field-collected relative humidity 2 years prior on the density of infected adults (DIA). The DIA is an estimate of the number of infected questing *I. ricinus* adults per 100 m^2^ sampled by the dragging method each year. The mean annual relative humidity has units of % and was measured at 50 cm above ground on the day of sampling at the field site. The parameter estimates used to calculate the effect sizes were taken from the model-averaged parameter estimates in Table S8. Increasing mean annual relative humidity 2 years prior by one standard deviation (e.g. 7.0% of relative humidity) decreased the DIA by 32.9% at each of the four elevation sites.

# Section 5 – Full statistical analysis of the nymphal infection prevalence (NIP) for the restricted 13-year period of the study (2006 – 2018)

**Model selection approach:** To identify the best model, we used a model selection approach based on the Akaike information criterion (AIC). A big conceptual advantage of model selection is that it reminds the user that there are competing models with different parameter estimates and that some of these models have more support (i.e., are better at explaining the data) than other models. Another advantage is that you can compare non-nested models containing different explanatory variables, which is not possible with a more traditional approach like stepwise multiple regression. Models were ranked according to their AIC values and the Akaike weights, which indicate the percent support, were calculated for each model. We used the Akaike weights to calculate the model-averaged parameter estimates and their 95% confidence intervals (CIs). For the generalized linear mixed effects models that analysed the NIP and AIP, we assessed the goodness of fit of the binomial distribution for the best model from the model selection table. Similarly, for the linear models that analysed the DIN and DIA, the assumptions of normally distributed residuals and equal variances were assessed for the best model from the model selection table (Additional file 1: Section 3). We used R version 4.0.3 for all statistical analyses (Team 2013). We used the lm() function in the base package to run the LMs with normal errors. We used the glmer() functions in the lme4 package to run the GLMMs with binomial errors. We used the mod.sel() function and the model.av() function in the MuMIn package to create the model selection tables and the model-averaged parameter estimates.

**Model selection analysis of the NIP:** The model selection table for the 30 best out of 232 models is presented in Table S9. For the NIP, the best two models had a combined support of 94.0% (Table S9). These two models each had 47.0% of the support (Table S9) and contained the explanatory variables year, RLB time lag, and weather station mean annual precipitation in the present year (e.g. no time lag). The only difference between these two models was that the second model contained elevation site, whereas the first model did not (Table S9).

For the individual explanatory variables, there was strong support for RLB time lag (100.0%), year (100.0%), and weather station mean annual precipitation in the present year (93.8%), moderate support for site (48.2%), and low support for weather station mean annual relative humidity in the present year (5.7%; Table S10). None of the other explanatory variables had a support > 1.0% (Table S10).

**Model-averaged parameter estimates for the NIP:** To determine the direction and statistical significance of the explanatory variables on the NIP, we present the model-averaged parameter estimates (and their 95% confidence intervals) on the logit scale (Table S11). We also back-calculated the effect sizes of the explanatory variables on the NIP on the original scale with respect to the following baseline: the site was low elevation, the year was 2006, and the covariates of RLB time lag and weather station precipitation in the present year were set to 0 (i.e., the mean values on the z-score scale).

The NIP was significantly different between the four elevation sites (Table S11). Compared to the low elevation site, the NIP was 13.4% lower at the medium (Medium – Low contrast = -0.195, 95% CI = -0.467 – 0.077), 23.6% lower at the high (High – Low contrast = -0.358, 95% CI = -0.653 – -0.063), and 9.4% lower at the top (Top – Low contrast = -0.135, 95% CI = -0.481 – 0.211) elevation sites.

Year had a negative and significant effect (slope = -0.145 per year, 95% CI = -0.181 – -0.108), indicating that the NIP was decreasing over time at Chaumont Mountain (Table S11). Over the 13-year period of the study (2006 – 2018), the NIP on the original scale decreased by 77.1% to 78.6% at the four elevation sites (Table S11).

The RLB time lag had a negative and significant effect on the NIP (Table S11; slope = -0.184 per standard deviation, 95% CI = -0.285 – -0.083). Increasing the RLB time lag by one standard deviation (e.g., 881 days) decreased the NIP on the original scale by 12.6% to 13.6% at the four elevation sites (Table S11).

The weather station mean annual precipitation in the present year had a negative and significant effect on the NIP (Table S11; slope = -0.311 per standard deviation, 95% CI = -0.447 – -0.174). Increasing the weather station mean annual precipitation in the present year by one standard deviation (e.g., 0.5 mm of precipitation) decreased the NIP on the original scale by 22.4% to 24.0% at the four elevation sites (Table S11).

In summary, the explanatory variables of year, RLB time lag, and precipitation in the same year all had significant negative effects on the NIP.

Table S9. Model selection results are shown for the generalized linear mixed effects model with binomial errors of the nymphal infection prevalence (NIP) response variable. The explanatory variables were site, year, beech masting index 2 years prior, RLB time lag, and the climate variables obtained from the weather stations and collected from the field. The models are ranked according to their Akaike Information Criterion (AIC). Shown for each model are the model rank (Rank), model structure (see below for explanation of explanatory variables), model degrees of freedom (Df), log-likelihood (logLik), Akaike information criterion (AIC), difference in the AIC value from the top model (ΔAIC), model weight (Weight1), and cumulative weight (Weight2).

| **Rank** | **Model structure** | **Df** | **logLik** | **AIC** | **ΔAIC** | **Weight1** | **Weight2** |
| --- | --- | --- | --- | --- | --- | --- | --- |
| 1 | NIP ~ Y+RLB+PR | 5 | -2360.1 | 4736.2 | 0.0 | 47.0 | 47.0 |
| 2 | NIP ~ S+Y+RLB+PR | 8 | -2363.1 | 4736.2 | 0.0 | 47.0 | 94.0 |
| 3 | NIP ~ Y+RLB+RH1 | 5 | -2365.4 | 4740.8 | 4.6 | 5.0 | 99.0 |
| 4 | NIP ~ S+Y+RLB+RH1 | 8 | -2363.9 | 4743.8 | 7.6 | 1.0 | 100.0 |
| 5 | NIP ~ Y+RLB+SD1 | 5 | -2368.3 | 4746.5 | 10.3 | 0.0 | 100.0 |
| 6 | NIP ~ S+Y+RLB+SD1 | 8 | -2366.8 | 4749.5 | 13.3 | 0.0 | 100.0 |
| 7 | NIP ~ S+Y+RLB+RH2 | 8 | -2367.7 | 4751.5 | 15.3 | 0.0 | 100.0 |
| 8 | NIP ~ Y+RLB+T2_y-1_ | 8 | -2368.0 | 4752.1 | 15.9 | 0.0 | 100.0 |
| 9 | NIP ~ Y+RLB+T1_y-2_ | 5 | -2371.1 | 4752.2 | 16.0 | 0.0 | 100.0 |
| 10 | NIP ~ S+Y+RLB+SD2_y-1_ | 8 | -2368.5 | 4753.1 | 16.9 | 0.0 | 100.0 |
| 11 | NIP ~ S+Y+RLB+PRy-1 | 8 | -2368.6 | 4753.3 | 17.1 | 0.0 | 100.0 |
| 12 | NIP ~ Y+DINy-1+RLB | 5 | -2371.9 | 4753.8 | 17.6 | 0.0 | 100.0 |
| 13 | NIP ~ Y+RLB+PRy-1 | 5 | -2371.9 | 4753.9 | 17.7 | 0.0 | 100.0 |
| 14 | NIP ~ Y+RLB+T1 | 5 | -2371.9 | 4753.9 | 17.7 | 0.0 | 100.0 |
| 15 | NIP ~ Y+RLB+T2 | 5 | -2372.0 | 4754.1 | 17.9 | 0.0 | 100.0 |
| 16 | NIP ~ S+Y+RLB | 7 | -2370.2 | 4754.4 | 18.2 | 0.0 | 100.0 |
| 17 | NIP ~ S+Y+RLB+RH1y-2 | 8 | -2369.6 | 4755.2 | 19.0 | 0.0 | 100.0 |
| 18 | NIP ~ S+Y+RLB+T1y-1 | 8 | -2369.6 | 4755.3 | 19.1 | 0.0 | 100.0 |
| 19 | NIP ~ S+Y+RLB+T1y-2 | 8 | -2369.7 | 4755.3 | 19.1 | 0.0 | 100.0 |
| 20 | NIP ~ S+Y+DINy-1+RLB | 8 | -2369.7 | 4755.4 | 19.2 | 0.0 | 100.0 |
| 21 | NIP ~ Y+RLB+SD1y_1 | 5 | -2372.7 | 4755.4 | 19.2 | 0.0 | 100.0 |
| 22 | NIP ~ S+Y+RLB+SD2 | 8 | -2369.7 | 4755.4 | 19.2 | 0.0 | 100.0 |
| 23 | NIP ~ S+Y+RLB+RH2y-1 | 8 | -2369.7 | 4755.5 | 19.3 | 0.0 | 100.0 |
| 24 | NIP ~ S+Y+RLB+T2 | 8 | -2369.9 | 4755.8 | 19.6 | 0.0 | 100.0 |
| 25 | NIP ~ S+Y+RLB+RH2y-2 | 8 | -2370.1 | 4756.2 | 20.0 | 0.0 | 100.0 |
| 26 | NIP ~ Y+RLB+SD2y-2 | 5 | -2373.1 | 4756.2 | 20.0 | 0.0 | 100.0 |
| 27 | NIP ~ S+Y+RLB+PRy-2 | 8 | -2370.1 | 4756.3 | 20.1 | 0.0 | 100.0 |
| 28 | NIP ~ Y+RLB+SD1y-2 | 5 | -2373.1 | 4756.3 | 20.1 | 0.0 | 100.0 |
| 29 | NIP ~ S+Y+RLB+SD1y-2 | 8 | -2370.1 | 4756.3 | 20.1 | 0.0 | 100.0 |
| 30 | NIP ~ Y+RLB+RH1y-1 | 5 | -2373.1 | 4756.3 | 20.1 | 0.0 | 100.0 |

Table S10. The support for each explanatory variable is shown from the AIC-based model selection table of the nymphal infection prevalence. This support is calculated as the sum of the Akaike weights for all the models in the set that include that explanatory variable.

| **Rank** | **Explanatory variable of interest** | **Support (%)** |
| --- | --- | --- |
| 1 | RLB | 100.0 |
| 2 | Year | 100.0 |
| 3 | PR | 93.8 |
| 4 | Site | 48.2 |
| 5 | RH1 | 5.7 |
| 6 | SD1 | < 1.0 |
| 7 | Beech | < 1.0 |
| 8 | DIN_y-1_ | < 1.0 |
| 9 | T1 | < 1.0 |
| 10 | T1_y-1_ | < 1.0 |
| 11 | RH1_y-1_ | < 1.0 |
| 12 | SD1_y-1_ | < 1.0 |
| 13 | PR_y-1_ | < 1.0 |
| 14 | T1_y-2_ | < 1.0 |
| 15 | RH1_y-2_ | < 1.0 |
| 16 | SD1_y-2_ | < 1.0 |
| 17 | PR_y-2_ | < 1.0 |
| 18 | T2 | < 1.0 |
| 19 | RH2 | < 1.0 |
| 20 | SD2 | < 1.0 |
| 21 | T2_y-1_ | < 1.0 |
| 22 | RH2_y-1_ | < 1.0 |
| 23 | SD2_y-1_ | < 1.0 |
| 24 | T2_y-2_ | < 1.0 |
| 25 | RH2_y-2_ | < 1.0 |
| 26 | SD2_y-2_ | < 1.0 |
| 27 | Site:Year | < 1.0 |
| 28 | Site:Beech | < 1.0 |
| 29 | Site:DINy-1 | < 1.0 |
| 30 | Site:RLB | < 1.0 |
| 31 | Site:T1 | < 1.0 |
| 32 | Site:RH1 | < 1.0 |
| 33 | Site:SD1 | < 1.0 |
| 34 | Site:PR | < 1.0 |
| 35 | Site:T1_y-1_ | < 1.0 |
| 36 | Site:RH1_y-1_ | < 1.0 |
| 37 | Site:SD1_y-1_ | < 1.0 |
| 38 | Site:PR_y-1_ | < 1.0 |
| 39 | Site:T1_y-2_ | < 1.0 |
| 40 | Site:RH1_y-2_ | < 1.0 |
| 41 | Site:SD1_y-2_ | < 1.0 |
| 42 | Site:PR_y-2_ | < 1.0 |
| 43 | Site:T2 | < 1.0 |
| 44 | Site:RH2 | < 1.0 |
| 45 | Site:SD2 | < 1.0 |
| 46 | Site:T2_y-1_ | < 1.0 |
| 47 | Site:RH2_y-1_ | < 1.0 |
| 48 | Site:SD2_y-1_ | < 1.0 |
| 49 | Site:T2_y-2_ | < 1.0 |
| 50 | Site:RH2_y-2_ | < 1.0 |
| 51 | Site:SD2_y-2_ | < 1.0 |

Table S11. Model-averaged parameter estimates are shown for the generalized linear mixed effects model with binomial errors of the nymphal infection prevalence (NIP) response variable. Shown are the parameter types, the parameter names, the parameter estimates, and the 95% confidence limits (LL = lower limit and UL = upper limit). Estimate 1 is averaged over all the models in the set. Estimate 2 is averaged over the subset of models with a cumulative support of 95%. The 95% confidence limits are for estimate 2.

| **Type** | **Name** | **Estimate 1** | **Estimate 2** | **95% LL** | **95% UL** |
| --- | --- | --- | --- | --- | --- |
| **Intercept** | **Low site** | **-0.783** | **-0.783** | **-1.161** | **-0.405** |
| Contrast 1 | Medium site | -0.094 | -0.195 | -0.467 | 0.077 |
| **Contrast 2** | **High site** | **-0.172** | **-0.358** | **-0.653** | **-0.063** |
| Contrast 3 | Top site | -0.065 | -0.135 | -0.481 | 0.211 |
| **Slope 1** | **Year** | **-0.145** | **-0.145** | **-0.181** | **-0.108** |
| Slope 2 | Beech | 0.000 | 0.002 | -0.073 | 0.077 |
| Slope 3 | DIN_y-1_ | 0.000 | 0.155 | -0.024 | 0.333 |
| **Slope 4** | **RLB** | **-0.184** | **-0.184** | **-0.285** | **-0.083** |
| Contrast 4 | T1 | 0.000 | 0.146 | -0.069 | 0.361 |
| **Contrast 5** | **RH1** | **-0.018** | **-0.311** | **-0.447** | **-0.174** |
| Contrast 6 | SD1 | 0.001 | 0.282 | 0.119 | 0.446 |
| **Contrast 7** | **PR** | **-0.318** | **-0.339** | **-0.467** | **-0.211** |
| Contrast 8 | T1_y-1_ | 0.000 | -0.055 | -0.426 | 0.316 |
| Contrast 9 | RH1_y-1_ | 0.000 | -0.080 | -0.266 | 0.106 |
| Contrast 10 | SD1_y-1_ | 0.000 | 0.098 | -0.122 | 0.319 |
| Contrast 11 | PR_y-1_ | 0.000 | -0.149 | -0.298 | 0.001 |
| Contrast 12 | T1_y-2_ | 0.000 | 0.187 | 0.013 | 0.361 |
| Contrast 13 | RH1_y-2_ | 0.000 | 0.078 | -0.135 | 0.291 |
| Contrast 14 | SD1_y-2_ | 0.000 | 0.044 | -0.208 | 0.296 |
| Contrast 15 | PR_y-2_ | 0.000 | 0.017 | -0.133 | 0.168 |
| Contrast 16 | Medium site:Year | 0.000 | -0.037 | -0.132 | 0.057 |
| Contrast 17 | High site:Year | 0.000 | -0.056 | -0.154 | 0.042 |
| Contrast 18 | Top site:Year | 0.000 | -0.034 | -0.145 | 0.076 |
| Contrast 19 | Medium site:Beech | 0.000 | -0.089 | -0.349 | 0.172 |
| Contrast 20 | High site:Beech | 0.000 | -0.059 | -0.324 | 0.206 |
| Contrast 21 | Top site:Beech | 0.000 | 0.049 | -0.226 | 0.324 |
| Contrast 22 | Medium site:DIN_y-1_ | 0.000 | 0.499 | -0.215 | 1.212 |
| Contrast 23 | High site:DIN_y-1_ | 0.000 | 0.206 | -0.425 | 0.838 |
| Contrast 24 | Top site:DIN_y-1_ | 0.000 | 0.274 | -0.350 | 0.898 |
| Contrast 25 | Medium site:RLB | 0.000 | 0.141 | -0.166 | 0.448 |
| Contrast 26 | High site: RLB | 0.000 | -0.133 | -0.405 | 0.139 |
| Contrast 27 | Top site: RLB | 0.000 | -0.015 | -0.385 | 0.354 |
| Contrast 28 | Medium site:T1 | 0.000 | 0.174 | -0.697 | 1.046 |
| Contrast 29 | High site:T1 | 0.000 | 0.052 | -0.813 | 0.916 |
| Contrast 30 | Top site:T1 | 0.000 | 0.321 | -0.563 | 1.205 |
| Contrast 31 | Medium site:RH1 | 0.000 | -0.003 | -0.581 | 0.575 |
| Contrast 32 | High site:RH1 | 0.000 | 0.140 | -0.442 | 0.722 |
| Contrast 33 | Top site:RH1 | 0.000 | 0.096 | -0.498 | 0.689 |
| Contrast 34 | Medium site:SD1 | 0.000 | -0.053 | -0.702 | 0.596 |
| Contrast 35 | High site:SD1 | 0.000 | -0.294 | -0.987 | 0.400 |
| Contrast 36 | Top site:SD1 | 0.000 | -0.133 | -0.923 | 0.656 |
| Contrast 37 | Medium site:PR | 0.000 | -0.010 | -0.505 | 0.485 |
| Contrast 38 | High site:PR | 0.000 | 0.001 | -0.501 | 0.503 |
| Contrast 39 | Top site:PR | 0.000 | 0.164 | -0.354 | 0.683 |
| Contrast 40 | Medium site:T_y-1_ | 0.000 | -0.325 | -1.177 | 0.526 |
| Contrast 41 | High site:T_y-1_ | 0.000 | -0.532 | -1.376 | 0.312 |
| Contrast 42 | Top site:T_y-1_ | 0.000 | -0.488 | -1.351 | 0.374 |
| Contrast 43 | Medium site:RH1_y-1_ | 0.000 | 0.000 | -0.593 | 0.593 |
| Contrast 44 | High site:RH1_y-1_ | 0.000 | 0.208 | -0.393 | 0.810 |
| Contrast 45 | Top site:RH1_y-1_ | 0.000 | 0.175 | -0.453 | 0.803 |
| Contrast 46 | Medium site:SD1_y-1_ | 0.000 | -0.010 | -0.666 | 0.646 |
| Contrast 47 | High site:SD1_y-1_ | 0.000 | -0.210 | -0.908 | 0.488 |
| Contrast 48 | Top site:SD1_y-1_ | 0.000 | -0.298 | -1.112 | 0.515 |
| Contrast 49 | Medium site:PR_y-1_ | 0.000 | -0.094 | -0.582 | 0.395 |
| Contrast 50 | High site:PR_y-1_ | 0.000 | -0.003 | -0.500 | 0.494 |
| Contrast 51 | Top site:PR_y-1_ | 0.000 | -0.076 | -0.595 | 0.442 |
| Contrast 52 | Medium site:T1_y-2_ | 0.000 | -0.261 | -1.154 | 0.632 |
| Contrast 53 | High site:T1_y-2_ | 0.000 | -0.264 | -1.151 | 0.623 |
| Contrast 54 | Top site:T1_y-2_ | 0.000 | -0.280 | -1.180 | 0.621 |
| Contrast 55 | Medium site:RH1_y-2_ | 0.000 | 0.193 | -0.431 | 0.816 |
| Contrast 56 | High site:RH1_y-2_ | 0.000 | 0.291 | -0.337 | 0.918 |
| Contrast 57 | Top site:RH1_y-2_ | 0.000 | 0.251 | -0.385 | 0.886 |
| Contrast 58 | Medium site:SD1_y-2_ | 0.000 | -0.352 | -1.052 | 0.348 |
| Contrast 59 | High site:SD1_y-2_ | 0.000 | -0.459 | -1.201 | 0.283 |
| Contrast 60 | Top site:SD1_y-2_ | 0.000 | -0.359 | -1.177 | 0.460 |
| Contrast 61 | Medium site:PR_y-2_ | 0.000 | 0.113 | -0.362 | 0.588 |
| Contrast 62 | High site:PR_y-2_ | 0.000 | 0.104 | -0.385 | 0.593 |
| Contrast 63 | Top site:PR_y-2_ | 0.000 | 0.059 | -0.455 | 0.574 |
| Contrast 64 | T2 | 0.000 | 0.141 | -0.036 | 0.318 |
| Contrast 65 | RH2 | 0.000 | 0.175 | 0.020 | 0.330 |
| Contrast 66 | SD2 | 0.000 | -0.061 | -0.293 | 0.171 |
| Contrast 67 | T2_y-1_ | 0.000 | -0.194 | -0.388 | 0.000 |
| Contrast 68 | RH2_y-1_ | 0.000 | 0.069 | -0.113 | 0.250 |
| Contrast 69 | SD2_y-1_ | 0.000 | -0.169 | -0.374 | 0.036 |
| Contrast 70 | T2_y-2_ | 0.000 | 0.061 | -0.158 | 0.279 |
| Contrast 71 | RH2_y-2_ | 0.000 | -0.080 | -0.277 | 0.116 |
| Contrast 72 | SD2_y-2_ | 0.000 | 0.072 | -0.175 | 0.319 |
| Contrast 73 | Medium site:T2 | 0.000 | 0.051 | -0.671 | 0.774 |
| Contrast 74 | High site:T2 | 0.000 | -0.070 | -0.808 | 0.668 |
| Contrast 75 | Top site:T2 | 0.000 | -0.072 | -0.836 | 0.692 |
| Contrast 76 | Medium site:RH2 | 0.000 | 0.035 | -0.464 | 0.533 |
| Contrast 77 | High site:RH2 | 0.000 | 0.054 | -0.516 | 0.624 |
| Contrast 78 | Top site:RH2 | 0.000 | 0.279 | -0.336 | 0.893 |
| Contrast 79 | Medium site:SD2 | 0.000 | 0.069 | -0.495 | 0.632 |
| Contrast 80 | High site:SD2 | 0.000 | 0.024 | -0.681 | 0.730 |
| Contrast 81 | Top site:SD2 | 0.000 | -0.426 | -1.372 | 0.520 |
| Contrast 82 | Medium site:T2_y-1_ | 0.000 | -0.166 | -0.867 | 0.534 |
| Contrast 83 | High site:T2_y-1_ | 0.000 | -0.527 | -1.230 | 0.176 |
| Contrast 84 | Top site:T2_y-1_ | 0.000 | -0.331 | -1.053 | 0.390 |
| Contrast 85 | Medium site:RH2_y-1_ | 0.000 | -0.060 | -0.551 | 0.430 |
| Contrast 86 | High site:RH2_y-1_ | 0.000 | -0.031 | -0.567 | 0.504 |
| Contrast 87 | Top site:RH2_y-1_ | 0.000 | 0.217 | -0.359 | 0.794 |
| Contrast 88 | Medium site:SD2_y-1_ | 0.000 | 0.090 | -0.451 | 0.631 |
| Contrast 89 | High site:SD2_y-1_ | 0.000 | 0.025 | -0.592 | 0.642 |
| Contrast 90 | Top site:SD2_y-1_ | 0.000 | -0.004 | -0.771 | 0.764 |
| Contrast 91 | Medium site:T2_y-2_ | 0.000 | 0.068 | -0.600 | 0.736 |
| Contrast 92 | High site:T2_y-2_ | 0.000 | -0.014 | -0.691 | 0.663 |
| Contrast 93 | Top site:T2_y-2_ | 0.000 | -0.298 | -0.998 | 0.402 |
| Contrast 94 | Medium site:RH2_y-2_ | 0.000 | -0.023 | -0.466 | 0.420 |
| Contrast 95 | High site:RH2_y-2_ | 0.000 | 0.079 | -0.398 | 0.555 |
| Contrast 96 | Top site:RH2_y-2_ | 0.000 | 0.260 | -0.248 | 0.768 |
| Contrast 97 | Medium site:SD2_y-2_ | 0.000 | 0.067 | -0.408 | 0.543 |
| Contrast 98 | High site:SD2_y-2_ | 0.000 | 0.012 | -0.525 | 0.549 |
| Contrast 99 | Top site:SD2_y-2_ | 0.000 | -0.146 | -0.802 | 0.510 |

# Section 6 – Full statistical analysis of the density of infected nymphs (DIN) for the restricted 13-year period of the study (2006 – 2018)

**Model selection approach:** To identify the best model, we used a model selection approach based on the Akaike information criterion (AIC). A big conceptual advantage of model selection is that it reminds the user that there are competing models with different parameter estimates and that some of these models have more support (i.e., are better at explaining the data) than other models. Another advantage is that you can compare non-nested models containing different explanatory variables, which is not possible with a more traditional approach like stepwise multiple regression. Models were ranked according to their AIC values and the Akaike weights, which indicate the percent support, were calculated for each model. We used the Akaike weights to calculate the model-averaged parameter estimates and their 95% confidence intervals (CIs). For the generalized linear mixed effects models that analysed the NIP and AIP, we assessed the goodness of fit of the binomial distribution for the best model from the model selection table. Similarly, for the linear models that analysed the DIN and DIA, the assumptions of normally distributed residuals and equal variances were assessed for the best model from the model selection table (Additional file 1: Section 3). We used R version 4.0.3 for all statistical analyses (Team 2013). We used the lm() function in the base package to run the LMs with normal errors. We used the glmer() functions in the lme4 package to run the GLMMs with binomial errors. We used the mod.sel() function and the model.av() function in the MuMIn package to create the model selection tables and the model-averaged parameter estimates.

**Model selection analysis of the DIN:** The model selection table for the best 30 out of 314 models is presented in Table S12. For the annual DIN, the top three models had a combined support of 80.0% (Table S12). The best model had 47.0% of the support (Table S12), explained 82.5% of the variation in the annual DIN, and contained the explanatory variables of elevation site (partial r^2^ = 28.3%), year (partial r^2^ = 14.8%), site:year interaction (partial r^2^ = 4.2%), beech mast score 2 years prior (partial r^2^ = 5.8%), and weather station mean annual relative humidity in the present year (partial r^2^ = 6.1%).

The support for the individual explanatory variables was as follows: site (100.0%), year (100.0%), site:year interaction (92.0%), beech mast score 2 year prior (69.1%), weather station mean annual relative humidity in the present year (50.8%), RLB time lag (31.2%), weather station mean annual precipitation in the previous year (20.0%), and weather station mean annual saturation deficit in the present year (16.1%; Table S13). None of the other explanatory variables had a support > 2.5% (Table S13).

**Model-averaged parameter estimates for the DIN:** To determine the effects of the explanatory variables on the DIN, we present the model-averaged parameter estimates on the log10-transformed scale (Table S14). We also back-calculated the effect sizes of the explanatory variables on the DIN on the original scale with respect to the following baseline: the site was low elevation, the year was 2006, the beech mast score was set to 1, and the other covariates were set to 0 (i.e., the mean values on the z-score scale).

The interaction between site and year indicated that the change in the DIN over time differed between the four elevation sites (Table S14). Over the 13-year period (2006 – 2018), the DIN decreased at the low (slope = -0.018 per year, 95% CI = -0.059 – 0.023), medium (Medium – Low contrast of the slope = -0.023, 95% CI = -0.072 – 0.027), high (High – Low contrast of the slope = -0.027, 95% CI = -0.076 – 0.023), and top (Top – Low contrast of the slope = -0.088, 95% CI = -0.138 – -0.037) elevation sites. Over the 13-year period (2006 – 2018), the DIN decreased by 38.7%, 67.2%, 70.7% and 94.6% at the low, medium, high, and top elevation sites, respectively (Table S14). Due to the significant interaction between site and year, it does not make sense to interpret the differences in intercept between the four elevation sites (Table S14).

The beech mast score 2 years prior had a positive and significant effect on the annual DIN (Table S14; slope = 0.067 per class; 95% CI = 0.029 – 0.105). Increasing the beech mast score 2 years prior from 1 (poor mast) to 5 (full mast) increased the DIN by 85.5% at each of the four elevation sites on Chaumont Mountain (Table S14). The weather station mean annual relative humidity in the present year had a negative and significant effect on the DIN (Table S14; slope = -0.166 per standard deviation, 95% CI = -0.253 – -0.079). Increasing the weather station mean annual relative humidity in the present year by one standard deviation (1.8% of relative humidity) decreased the DIN by 31.8% at each of the four elevation sites on Chaumont Mountain (Table S14).

Other models contained other explanatory variables that had the following effects on the DIN. The RLB time lag had a negative and significant effect on the DIN (Table S14; slope = -0.131 per standard deviation, 95% CI = -0.215 – -0.047). The weather station mean annual precipitation in the previous year had a negative and significant effect on the DIN (Table S14; slope = -0.108 per standard deviation, 95% CI = -0.183 – -0.033). The weather station mean annual saturation deficit in the present year had a positive and significant effect on the DIN (Table S14; slope = 0.205 per standard deviation, 95% CI = 0.079 – 0.330).

In summary, the DIN decreased over time at the four elevation sites and significantly so at the top elevation. The DIN increased significantly with tree seed production two years prior and decreased significantly with the relative humidity in the present year.

Table S12. Model selection results are shown for the linear models with normal errors of the log10-trasnformed density of infected nymphs (DIN) response variable. The explanatory variables were site, year, beech masting index 2 years prior, RLB time lag, and the climate variables obtained from the weather stations and collected from the field. The models are ranked according to their Akaike Information Criterion (AIC). Shown for each model are the model rank (Rank), model structure (see below for explanation of explanatory variables), model degrees of freedom (Df), log-likelihood (logLik), Akaike information criterion (AIC), difference in the AIC value from the top model (ΔAIC), model weight (Weight1), and cumulative weight (Weight2), and adjusted r-squared (r^2^).

| **Rank** | **Model structure** | **Df** | **logLik** | **AIC** | **ΔAIC** | **Weight1** | **Weight2** | **r^2^** |
| --- | --- | --- | --- | --- | --- | --- | --- | --- |
| 1 | DIN ~ S+Y+B+RH1+S:Y | 11 | 8.1 | 12.3 | 0.0 | 47.0 | 47.0 | 82.5 |
| 2 | DIN ~ S+Y+RLB+PR_y-1_+S:Y | 11 | 7.2 | 14.2 | 1.8 | 19.0 | 66.0 | 81.9 |
| 3 | DIN ~ S+Y+B+SD1+S:Y | 11 | 6.9 | 14.8 | 2.5 | 14.0 | 80.0 | 81.7 |
| 4 | DIN ~ S+Y+B+RH1 | 8 | 0.7 | 17.9 | 5.6 | 3.0 | 83.0 | 78.3 |
| 5 | DIN ~ S+Y+B+S:Y | 11 | 4.9 | 18.8 | 6.5 | 2.0 | 85.0 | 80.2 |
| 6 | DIN ~ Y+B+SD1 | 8 | 0.2 | 19.0 | 6.7 | 2.0 | 87.0 | 77.9 |
| 7 | DIN ~ S+Y+RLB+T1_y-1_+S:Y | 11 | 4.6 | 19.3 | 7.0 | 1.0 | 88.0 | 80.0 |
| 8 | DIN ~ S+Y+RLB+PR+S:Y | 11 | 4.1 | 20.3 | 8.0 | 1.0 | 89.0 | 79.6 |
| 9 | DIN ~ S+Y+RLB+RH1_y-1_+S:Y | 11 | 4.1 | 20.4 | 8.1 | 1.0 | 90.0 | 79.6 |
| 10 | DIN ~ S+Y+RLB+S:Y | 10 | 2.3 | 20.7 | 8.4 | 1.0 | 91.0 | 78.7 |
| 11 | DIN ~ S+Y+RLB+SD1+S:Y | 11 | 3.9 | 20.8 | 8.4 | 1.0 | 92.0 | 79.5 |
| 12 | DIN ~ S+Y+RLB+T1_y-2_ | 11 | 3.8 | 21.0 | 8.7 | 1.0 | 93.0 | 79.4 |
| 13 | DIN ~ S+Y+RLB+S:Y+S:RLB | 13 | 7.2 | 21.1 | 8.8 | 1.0 | 94.0 | 81.0 |
| 14 | DIN ~ S+Y+RLB+RH1+S:Y | 11 | 3.6 | 21.3 | 9.0 | 1.0 | 95.0 | 79.2 |
| 15 | DIN ~ S+Y+RLB+T2+S:Y | 11 | 3.6 | 21.4 | 9.1 | 0.0 | 100.0 | 79.2 |
| 16 | DIN ~ S+Y+RLB+SD1y-1+S:Y | 11 | 3.5 | 21.6 | 9.3 | 0.0 | 100.0 | 79.1 |
| 17 | DIN ~ S+Y+RLB+PRy-1 | 8 | -1.1 | 21.6 | 9.3 | 0.0 | 100.0 | 76.7 |
| 18 | DIN ~ S+Y+RLB+PRy-1 | 8 | -1.1 | 21.6 | 9.3 | 0.0 | 100.0 | 76.7 |
| 19 | DIN ~ S+Y+B+RLB | 8 | -1.4 | 22.2 | 9.9 | 0.0 | 100.0 | 76.4 |
| 20 | DIN ~ S+Y+RLB+RH2+S:Y | 11 | 3.2 | 22.2 | 9.9 | 0.0 | 100.0 | 78.9 |
| 21 | DIN ~ S+Y+B+RH2y-2+S:Y | 11 | 3.2 | 22.3 | 9.9 | 0.0 | 100.0 | 78.9 |
| 22 | DIN ~ S+Y+B+PRy-1+S:Y | 11 | 2.9 | 22.9 | 10.5 | 0.0 | 100.0 | 78.6 |
| 23 | DIN ~ S+Y+RLB+T2y-1+S:Y | 11 | 2.8 | 23.0 | 10.6 | 0.0 | 100.0 | 78.6 |
| 24 | DIN ~ S+Y+RLB+T1+S:Y | 11 | 2.7 | 23.2 | 10.8 | 0.0 | 100.0 | 78.5 |
| 25 | DIN ~ S+Y+RLB+RH1y-2+S:Y | 11 | 2.6 | 23.4 | 11.1 | 0.0 | 100.0 | 78.4 |
| 26 | DIN ~ S+Y+RLB+SD2y-2+S:Y | 11 | 2.5 | 23.6 | 11.3 | 0.0 | 100.0 | 78.3 |
| 27 | DIN ~ S+Y+B+PR+S:Y | 11 | 2.4 | 23.7 | 11.4 | 0.0 | 100.0 | 78.3 |
| 28 | DIN ~ S+Y+RLB+S:RLB | 10 | 0.8 | 23.8 | 11.5 | 0.0 | 100.0 | 77.3 |
| 29 | DIN ~ S+Y+RLB+T2y-2+S:Y | 11 | 2.4 | 23.9 | 11.5 | 0.0 | 100.0 | 78.2 |
| 30 | DIN ~ S+Y+B+T1+S:Y | 11 | 2.4 | 23.9 | 11.6 | 0.0 | 100.0 | 78.2 |

Table S13. The support for each explanatory variable is shown from the AIC-based model selection table of the density of infected nymphs. This support is calculated as the sum of the Akaike weights for all the models in the set that include that explanatory variable.

| **Rank** | **Explanatory variable of interest** | **Support (%)** |
| --- | --- | --- |
| 1 | Site | 100.0 |
| 2 | Year | 100.0 |
| 3 | Site:Year | 92.0 |
| 4 | Beech | 69.1 |
| 5 | RH1 | 50.8 |
| 6 | RLB | 31.2 |
| 7 | PR_y-1_ | 20.0 |
| 8 | SD1 | 16.1 |
| 9 | T2 | 2.5 |
| 10 | T1_y-1_ | 1.7 |
| 11 | PR | 1.2 |
| 12 | T1 | < 1.0 |
| 13 | RH1_y-1_ | < 1.0 |
| 14 | SD1_y-1_ | < 1.0 |
| 15 | T1_y-2_ | < 1.0 |
| 16 | RH1_y-2_ | < 1.0 |
| 17 | SD1_y-2_ | < 1.0 |
| 18 | PR_y-2_ | < 1.0 |
| 19 | RH2 | < 1.0 |
| 20 | SD2 | < 1.0 |
| 21 | T2_y-1_ | < 1.0 |
| 22 | RH2_y-1_ | < 1.0 |
| 23 | SD2_y-1_ | < 1.0 |
| 24 | T2_y-2_ | < 1.0 |
| 25 | RH2_y-2_ | < 1.0 |
| 26 | SD2_y-2_ | < 1.0 |
| 27 | Site:Beech | < 1.0 |
| 28 | Site:RLB | < 1.0 |
| 29 | Site:T1 | < 1.0 |
| 30 | Site:RH1 | < 1.0 |
| 31 | Site:SD1 | < 1.0 |
| 32 | Site:PR | < 1.0 |
| 33 | Site:T1_y-1_ | < 1.0 |
| 34 | Site:RH1_y-1_ | < 1.0 |
| 35 | Site:SD1_y-1_ | < 1.0 |
| 36 | Site:PR_y-1_ | < 1.0 |
| 37 | Site:T1_y-2_ | < 1.0 |
| 38 | Site:RH1_y-2_ | < 1.0 |
| 39 | Site:SD1_y-2_ | < 1.0 |
| 40 | Site:PR_y-2_ | < 1.0 |
| 41 | Site:T2 | < 1.0 |
| 42 | Site:RH2 | < 1.0 |
| 43 | Site:SD2 | < 1.0 |
| 44 | Site:T2_y-1_ | < 1.0 |
| 45 | Site:RH2_y-1_ | < 1.0 |
| 46 | Site:SD2_y-1_ | < 1.0 |
| 47 | Site:T2_y-2_ | < 1.0 |
| 48 | Site:RH2_y-2_ | < 1.0 |
| 49 | Site:SD2_y-2_ | < 1.0 |

Table S14. Model-averaged parameter estimates are shown for the linear model with normal errors of the density of infected nymphs (DIN) response variable. Shown are the parameter types, the parameter names, the parameter estimates, and the 95% confidence limits (LL = lower limit and UL = upper limit). Estimate 1 is averaged over all the models in the set. Estimate 2 is averaged over the subset of models with a cumulative support of 95%. The 95% confidence limits are for estimate 2.

| **Type** | **Name** | **Estimate 1** | **Estimate 2** | **95% LL** | **95% UL** |
| --- | --- | --- | --- | --- | --- |
| **Intercept** | **Low site** | **3.362** | **3.362** | **2.898** | **3.827** |
| Contrast 1 | Medium site | 0.043 | 0.043 | -0.442 | 0.529 |
| Contrast 2 | High site | -0.192 | -0.192 | -0.718 | 0.333 |
| Contrast 3 | Top site | -0.199 | -0.199 | -0.896 | 0.498 |
| Slope 1 | Year | -0.018 | -0.018 | -0.059 | 0.023 |
| **Slope 2** | **Beech** | **0.046** | **0.067** | **0.029** | **0.105** |
| **Slope 3** | **RLB** | **-0.041** | **-0.131** | **-0.215** | **-0.047** |
| Contrast 4 | T1 | 0.000 | 0.111 | -0.088 | 0.311 |
| **Contrast 5** | **RH1** | **-0.084** | **-0.166** | **-0.253** | **-0.079** |
| **Contrast 6** | **SD1** | **0.033** | **0.205** | **0.079** | **0.330** |
| Contrast 7 | PR | -0.001 | -0.077 | -0.166 | 0.013 |
| Contrast 8 | T1_y-1_ | -0.003 | -0.160 | -0.335 | 0.015 |
| Contrast 9 | RH1_y-1_ | -0.001 | -0.075 | -0.168 | 0.019 |
| Contrast 10 | SD1_y-1_ | 0.000 | 0.082 | -0.042 | 0.206 |
| **Contrast 11** | **PR_y-1_** | **-0.022** | **-0.108** | **-0.183** | **-0.033** |
| Contrast 12 | T1_y-2_ | 0.001 | 0.120 | -0.050 | 0.290 |
| Contrast 13 | RH1_y-2_ | 0.000 | 0.050 | -0.062 | 0.163 |
| Contrast 14 | SD1_y-2_ | 0.000 | -0.017 | -0.147 | 0.114 |
| Contrast 15 | PR_y-2_ | 0.000 | -0.008 | -0.090 | 0.074 |
| Contrast 16 | Medium site:Year | -0.021 | -0.023 | -0.072 | 0.027 |
| Contrast 17 | High site:Year | -0.025 | -0.027 | -0.076 | 0.023 |
| **Contrast 18** | **Top site:Year** | **-0.081** | **-0.088** | **-0.138** | **-0.037** |
| Contrast 19 | Medium site:Beech | 0.000 | -0.029 | -0.154 | 0.095 |
| Contrast 20 | High site:Beech | 0.000 | 0.016 | -0.108 | 0.141 |
| Contrast 21 | Top site:Beech | 0.000 | -0.011 | -0.136 | 0.113 |
| Contrast 22 | Medium site:RLB | 0.000 | 0.049 | -0.161 | 0.260 |
| Contrast 23 | High site: RLB | 0.000 | 0.045 | -0.162 | 0.252 |
| Contrast 24 | Top site: RLB | 0.002 | 0.250 | 0.047 | 0.452 |
| Contrast 25 | Medium site:T1 | 0.000 | 0.054 | -0.458 | 0.567 |
| Contrast 26 | High site:T1 | 0.000 | 0.052 | -0.451 | 0.556 |
| Contrast 27 | Top site:T1 | 0.000 | -0.283 | -0.777 | 0.211 |
| Contrast 28 | Medium site:RH1 | 0.000 | 0.061 | -0.270 | 0.391 |
| Contrast 29 | High site:RH1 | 0.000 | 0.081 | -0.248 | 0.409 |
| Contrast 30 | Top site:RH1 | 0.000 | 0.179 | -0.142 | 0.499 |
| Contrast 31 | Medium site:SD1 | 0.000 | -0.076 | -0.435 | 0.283 |
| Contrast 32 | High site:SD1 | 0.000 | -0.115 | -0.489 | 0.259 |
| Contrast 33 | Top site:SD1 | 0.000 | -0.405 | -0.800 | -0.010 |
| Contrast 34 | Medium site:PR | 0.000 | 0.049 | -0.229 | 0.326 |
| Contrast 35 | High site:PR | 0.000 | -0.003 | -0.278 | 0.273 |
| Contrast 36 | Top site:PR | 0.000 | 0.222 | -0.054 | 0.498 |
| Contrast 37 | Medium site:T_y-1_ | 0.000 | -0.258 | -0.716 | 0.200 |
| Contrast 38 | High site:T_y-1_ | 0.000 | -0.379 | -0.827 | 0.068 |
| Contrast 39 | Top site:T_y-1_ | 0.000 | -0.621 | -1.058 | -0.185 |
| Contrast 40 | Medium site:RH1_y-1_ | 0.000 | 0.031 | -0.317 | 0.379 |
| Contrast 41 | High site:RH1_y-1_ | 0.000 | 0.099 | -0.246 | 0.445 |
| Contrast 42 | Top site:RH1_y-1_ | 0.000 | 0.147 | -0.189 | 0.484 |
| Contrast 43 | Medium site:SD1_y-1_ | 0.000 | -0.042 | -0.430 | 0.346 |
| Contrast 44 | High site:SD1_y-1_ | 0.000 | -0.111 | -0.514 | 0.291 |
| Contrast 45 | Top site:SD1_y-1_ | 0.000 | -0.348 | -0.764 | 0.068 |
| Contrast 46 | Medium site:PR_y-1_ | 0.000 | -0.060 | -0.341 | 0.220 |
| Contrast 47 | High site:PR_y-1_ | 0.000 | -0.008 | -0.288 | 0.272 |
| Contrast 48 | Top site:PR_y-1_ | 0.000 | 0.130 | -0.148 | 0.409 |
| Contrast 49 | Medium site:T1_y-2_ | 0.000 | -0.226 | -0.757 | 0.305 |
| Contrast 50 | High site:T1_y-2_ | 0.000 | -0.050 | -0.567 | 0.468 |
| Contrast 51 | Top site:T1_y-2_ | 0.000 | -0.371 | -0.875 | 0.132 |
| Contrast 52 | Medium site:RH1_y-2_ | 0.000 | 0.031 | -0.344 | 0.406 |
| Contrast 53 | High site:RH1_y-2_ | 0.000 | 0.061 | -0.306 | 0.427 |
| Contrast 54 | Top site:RH1_y-2_ | 0.000 | -0.038 | -0.389 | 0.313 |
| Contrast 55 | Medium site:SD1_y-2_ | 0.000 | -0.144 | -0.568 | 0.280 |
| Contrast 56 | High site:SD1_y-2_ | 0.000 | -0.118 | -0.551 | 0.315 |
| Contrast 57 | Top site:SD1_y-2_ | 0.000 | -0.189 | -0.627 | 0.249 |
| Contrast 58 | Medium site:PR_y-2_ | 0.000 | 0.034 | -0.251 | 0.320 |
| Contrast 59 | High site:PR_y-2_ | 0.000 | 0.020 | -0.267 | 0.308 |
| Contrast 60 | Top site:PR_y-2_ | 0.000 | 0.044 | -0.244 | 0.332 |
| Contrast 61 | T2 | 0.004 | 0.147 | 0.017 | 0.277 |
| Contrast 62 | RH2 | 0.000 | 0.059 | -0.058 | 0.176 |
| Contrast 63 | SD2 | 0.000 | 0.017 | -0.132 | 0.166 |
| Contrast 64 | T2_y-1_ | 0.000 | -0.050 | -0.167 | 0.066 |
| Contrast 65 | RH2_y-1_ | 0.000 | -0.026 | -0.156 | 0.104 |
| Contrast 66 | SD2_y-1_ | 0.000 | 0.002 | -0.131 | 0.136 |
| Contrast 67 | T2_y-2_ | 0.000 | -0.018 | -0.142 | 0.106 |
| Contrast 68 | RH2_y-2_ | 0.000 | -0.075 | -0.219 | 0.069 |
| Contrast 69 | SD2_y-2_ | 0.000 | -0.025 | -0.207 | 0.156 |
| Contrast 70 | Medium site:T2 | 0.000 | 0.006 | -0.413 | 0.426 |
| Contrast 71 | High site:T2 | 0.000 | 0.005 | -0.420 | 0.431 |
| Contrast 72 | Top site:T2 | 0.000 | -0.269 | -0.690 | 0.153 |
| Contrast 73 | Medium site:RH2 | 0.000 | -0.046 | -0.306 | 0.214 |
| Contrast 74 | High site:RH2 | 0.000 | -0.029 | -0.322 | 0.265 |
| Contrast 75 | Top site:RH2 | 0.000 | 0.316 | 0.018 | 0.614 |
| Contrast 76 | Medium site:SD2 | 0.000 | 0.076 | -0.228 | 0.379 |
| Contrast 77 | High site:SD2 | 0.000 | 0.073 | -0.305 | 0.451 |
| Contrast 78 | Top site:SD2 | 0.000 | -0.484 | -0.953 | -0.015 |
| Contrast 79 | Medium site:T2_y-1_ | 0.000 | -0.070 | -0.493 | 0.353 |
| Contrast 80 | High site:T2_y-1_ | 0.000 | -0.242 | -0.663 | 0.180 |
| Contrast 81 | Top site:T2_y-1_ | 0.000 | -0.191 | -0.617 | 0.235 |
| Contrast 82 | Medium site:RH2_y-1_ | 0.000 | -0.009 | -0.286 | 0.267 |
| Contrast 83 | High site:RH2_y-1_ | 0.000 | -0.042 | -0.339 | 0.255 |
| Contrast 84 | Top site:RH2_y-1_ | 0.000 | 0.201 | -0.106 | 0.508 |
| Contrast 85 | Medium site:SD2_y-1_ | 0.000 | 0.029 | -0.288 | 0.347 |
| Contrast 86 | High site:SD2_y-1_ | 0.000 | 0.025 | -0.331 | 0.381 |
| Contrast 87 | Top site:SD2_y-1_ | 0.000 | -0.062 | -0.487 | 0.362 |
| Contrast 88 | Medium site:T2_y-2_ | 0.000 | -0.042 | -0.492 | 0.407 |
| Contrast 89 | High site:T2_y-2_ | 0.000 | 0.047 | -0.399 | 0.493 |
| Contrast 90 | Top site:T2_y-2_ | 0.000 | 0.105 | -0.338 | 0.548 |
| Contrast 91 | Medium site:RH2_y-2_ | 0.000 | 0.054 | -0.211 | 0.319 |
| Contrast 92 | High site:RH2_y-2_ | 0.000 | 0.017 | -0.265 | 0.299 |
| Contrast 93 | Top site:RH2_y-2_ | 0.000 | 0.070 | -0.218 | 0.359 |
| Contrast 94 | Medium site:SD2_y-2_ | 0.000 | -0.046 | -0.349 | 0.258 |
| Contrast 95 | High site:SD2_y-2_ | 0.000 | 0.024 | -0.311 | 0.360 |
| Contrast 96 | Top site:SD2_y-2_ | 0.000 | 0.121 | -0.263 | 0.504 |

REFERENCES

Bregnard, C., O. Rais, and M. J. Voordouw. 2020. Climate and tree seed production predict the abundance of the European Lyme disease vector over a 15-year period. Parasit Vectors **13**:408.

Brugger, K., M. Walter, L. Chitimia-Dobler, G. Dobler, and F. Rubel. 2018. Forecasting next season’s *Ixodes ricinus* nymphal density: the example of southern Germany 2018. Experimental and Applied Acarology **75**:281-288.

Eisen, R. J., L. Eisen, M. B. Castro, and R. S. Lane. 2003. Environmentally related variability in risk of exposure to Lyme disease spirochetes in northern California: effect of climatic conditions and habitat type. Environmental Entomology **32**:1010-1018.

Ostfeld, R. S., C. D. Canham, K. Oggenfuss, R. J. Winchcombe, and F. Keesing. 2006. Climate, deer, rodents, and acorns as determinants of variation in Lyme-disease risk. PLoS Biology **4**:e145.

Perret, J. L., E. Guigoz, O. Rais, and L. Gern. 2000. Influence of saturation deficit and temperature on *Ixodes ricinus* tick questing activity in a Lyme borreliosis-endemic area (Switzerland). Parasitology Research **86**:554-557.

Team, R. C. 2013. R: A language and environment for statistical computing.
